# ﻿The arboreal snail genus *Amphidromus* Albers, 1850 (Eupulmonata, Camaenidae) of Southeast Asia: 1. Molecular systematics of some Vietnamese species and related species from Cambodia, Indonesia, and Laos

**DOI:** 10.3897/zookeys.1196.112146

**Published:** 2024-03-22

**Authors:** Parin Jirapatrasilp, Chih-Wei Huang, Chirasak Sutcharit, Chi-Tse Lee

**Affiliations:** 1 Animal Systematics Research Unit, Department of Biology, Faculty of Science, Chulalongkorn University, Bangkok, Thailand Chulalongkorn University Bangkok Thailand; 2 Leibniz Institute for the Analysis of Biodiversity Change, Martin-Luther-King-Platz 3, Hamburg, Germany Leibniz Institute for the Analysis of Biodiversity Change Hamburg Germany; 3 School of Life Science, National Taiwan Normal University, Taipei, Taiwan National Taiwan Normal University Taipei Taiwan; 4 Department of Life Sciences, National Chung Hsing University, Taichung, Taiwan National Chung Hsing University Taichung Taiwan

**Keywords:** Biodiversity, Helicoidei, shell polymorphism, Stylommatophora, taxonomy

## Abstract

This paper reassesses the taxonomy and systematics of 11 arboreal snail species in the genus *Amphidromus* from Vietnam, Cambodia, Indonesia and Laos (*A.bozhii* Wang, 2019, *A.buelowi* Fruhstorfer, 1905, *A.costifer* Smith, 1893, *A.haematostoma* Möllendorff, 1898, *A.ingens* Möllendorff, 1900, *A.madelineae* Thach, 2020, *A.metabletus* Möllendorff, 1900, *A.pankowskianus* Thach, 2020, *A.placostylus* Möllendorff, 1900, *A.roseolabiatus* Fulton, 1896, and *A.thachi* Huber, 2015). The taxonomic validity of each species is supported by a phylogenetic analysis of mitochondrial COI and 16S rRNA gene fragments from 17 ingroup taxa. *Amphidromusbuelowi* was found to comprise two populations from two distant localities, one from Mount Singgalang, West Sumatra, Indonesia and the other from southern Vietnam. The samples from southern Vietnam were previously described as *A.asper* Haas, 1934 and *A.franzhuberi* Thach, 2016, but they are now treated as junior synonyms of *A.buelowi* in this study. In addition, two species from Vietnam are described as new to science, viz. *A.asperoides* Jirapatrasilp & Lee, **sp. nov.** and *A.ingensoides* Jirapatrasilp & Lee, **sp. nov.**, each of which is conchologically comparable to *A.buelowi* and *A.ingens*, respectively.

## ﻿﻿Introduction

Since the comprehensive synoptic catalogue of the Southeast Asian arboreal snail genus *Amphidromus* Albers, 1850 by Laidlaw and Solem (1961), most papers on this genus involve the descriptions of new species. As a result, more than 150 species were newly introduced (MolluscaBase 2023). Some studies also revisited the taxonomic status of some species (e.g., Sutcharit et al. 2021; Verbinnen and Segers 2021), while others revised the taxonomy of *Amphidromus* in particular regions (Solem 1965; Sutcharit and Panha 2006; Inkhavilay et al. 2017; Poppe 2020), whereas still fewer papers focussed on molecular phylogenetic relationships (Sutcharit et al. 2007; Lee et al. 2022).

This paper is the first of a series that aims to revise the taxonomy and systematics of *Amphidromus* species from Southeast Asia, following the taxonomic reassessment of *A.cruentatus* (Morelet, 1875) in Lee et al. (2022). We revise some Vietnamese species and related species from Cambodia, Indonesia, and Laos that are phylogenetically related or that are conchologically similar to *A.cruentatus*. These include 11 nominal species: *A.bozhii* Wang, 2019, *A.buelowi* Fruhstorfer, 1905, *A.costifer* Smith, 1893, *A.haematostoma* Möllendorff, 1898, *A.ingens* Möllendorff, 1900, *A.madelineae* Thach, 2020, *A.metabletus* Möllendorff, 1900, *A.pankowskianus* Thach, 2020, *A.placostylus* Möllendorff, 1900, *A.roseolabiatus* Fulton, 1896, and *A.thachi* Huber, 2015. However, the original descriptions and subsequent treatments to delimit these species were primarily based on shell characteristics, which are extremely variable in the genus *Amphidromus* (Haniel 1921; Lee et al. 2022). Therefore, other lines of evidence, especially DNA sequence data, are crucial to define species boundaries more accurately and reassess the taxonomic status of *Amphidromus* species.

Against this background, new specimens of these 11 nominal species were collected in order to study their shell and anatomical characters, compare them with the available type material of species known from the study area, and to infer their phylogenetic relationships using DNA sequence data.

## ﻿﻿Materials and methods

### ﻿﻿Specimen preparation and preliminary species identification

**Figure 1.**
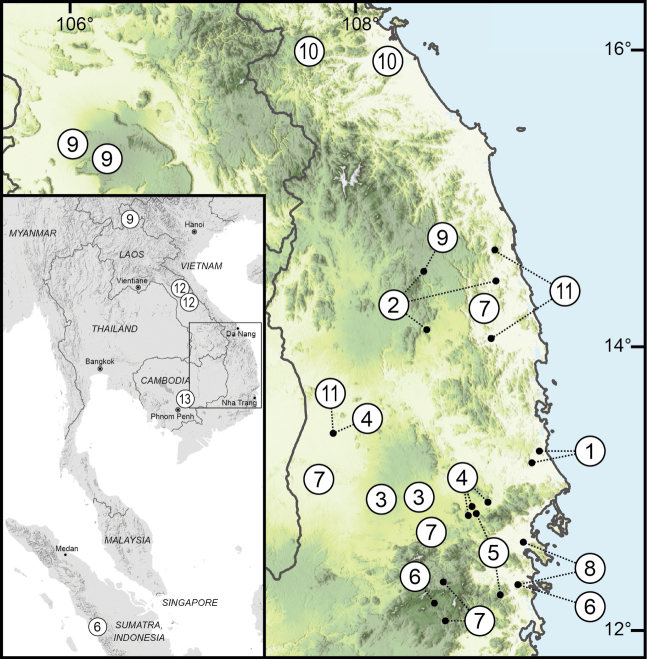
Distribution map of *Amphidromus* samples recognised in this study. 1. *Amphidromusbozhii*; 2. *Amphidromusplacostylus*; 3. *Amphidromusasperoides* sp. nov.; 4. *Amphidromusingens*; 5. *Amphidromusingensoides* sp. nov.; 6. *Amphidromusbuelowi*; 7. *Amphidromusthachi*; 8. *Amphidromusmetabletus*; 9. *Amphidromushaematostoma*; 10. *Amphidromusmadelineae*; 11. *Amphidromuscostifer*; 12. *Amphidromuspankowskianus*; 13. *Amphidromusroseolabiatus*. Species numbers correspond to those in Fig. 2 and Tables 1, 2. The map was produced using QGIS (3.16.0) with SRTM Downloader plugin (https://github.com/hdus/SRTM-Downloader), retrieving SRTM data from NASA Earth Data server (https://urs.earthdata.nasa.gov/).

Empty shells and living specimens were collected from several localities in Cambodia, Indonesia, Laos, and Vietnam (Fig. 1, Table 1). A total of 278 specimens was collected, and all specimens are deposited in the National Museum of Natural Science of Taiwan, Taichung, unless otherwise stated. Live specimens were photographed and fixed in 70% (v/v) ethanol for anatomical examination and 95% (v/v) for DNA analysis. The genitalia of 3–5 specimens per species were examined under a stereomicroscope, and one or two genitalia from each species were selected for photography. The radula of one specimen per species was examined with a scanning electron microscope (SEM; JEOL, JSM-5410 LV). At each collecting site, the specimens were collected within an area of approximately 100 m^2^. Shell measurements are based on adult specimens only.

**Table 1.** List of specimens used in this study with species name, locality details, voucher and GenBank accession numbers. Species numbers correspond to those in Figs 1, 2, and Table 2.

Number	Species	Preliminary species identification in this study	Specimen codes	Voucher numbers	Locality	No. of specimen and chirality	Figure	GenBank accession numbers		References
								COI	16S rRNA	
1	*Amphidromusbozhii* Wang, 2019	*A.bozhii*	XI0 to XI9	NMNS-8764-004 to NMNS-8764-013	Phu Hoa District, Phu Yen Province, Vietnam	10D	8D–E	XI0–XI8: OR977987–OR977995	XI0–XI2: OR964283– OR964285 XI5: OR964286	This study
		*A.bozhii*	XJ1 to XJ8	NMNS-8764-014 to NMNS-8764-021	Tuy Hoa District, Phu Yen Province, Vietnam	8S	8F	XJ1–XJ8: OR977996–OR978003	–	
2	*Amphidromusplacostylus* Möllendorff, 1900	*A.placostylus*	VAM0 to VAM4	NMNS-8764-213 to NMNS-8764-217	Dak Po District, Gia Lai Province, Vietnam	4D + 1S	11C–D	VAM1–VAM4: OR978004– OR978007	VAM1–VAM4: OR964287– OR964290	This study
		*A.placostylus*	VKAA1 to VKAA4	NMNS-8764-218 to NMNS-8764-221	Kbang, Gia Lai Province, Vietnam	3D + 1S	11E–F	VKAA2–VKAA4: OR978008– OR978010	VKAA2–VKAA4: OR964291– OR964293	
		*A.placostylus*	VKBB0 to VKBB9	NMNS-8764-222 to NMNS-8764-231	Hoai An, An Lao, Binh Dinh Province, Vietnam	4D + 6S	11G–I	VKBB0: OR978011 VKBB5: OR978012 VKBB9: OR978013	VKBB0: OR964294 VKBB5: OR964295 VKBB9: OR964296	
		*A.placostylus*	VKBN	NMNS-8764-232	Binh Dinh Province, Vietnam	1D	–	–	–	
		*A.placostylus*	VMEO1 to VMEO21	NMNS-8764-233 to NMNS-8764-253	Hoai An district, Binh Dinh Province, Vietnam	7D + 14S	–	–	–	
3	*Amphidromusasperoides* Jirapatrasilp & Lee sp. nov.	*Amphidromus* sp. 1	D2-1 to D2-4	NMNS-8764-001 to NMNS-8764-003, NHMUK 20230613	Ea Tu village, Buon Ma Thuat city, Dak Lak Province, Vietnam	4D	8A–C	OR978014– OR978017	OR964297– OR964300	This study
		*Amphidromus* sp. 1	VTAU1 to VTAU20	NMNS-8764-192 to NMNS-8764-211	Krong Pak, Dak Lak Province, Vietnam	20D	–	–	–	
4	*Amphidromusingens* Möllendorff, 1900	*A.ingens*	G3-1 to G3-5	NMNS-8764-082 to NMNS-8764-086	M’drak District, Dak Lak Province, Vietnam	4D + 1S	3D–E	G3-1: OR978018	–	This study
		*A.ingens*	R50	NMNS-8764-087	Ea M’doal Ward, M’drak District, Dak Lak Province, Vietnam	1D	3F	OR978019	OR964301	
		*A.ingens*	U20 to U24	NMNS-8764-088 to NMNS-8764-092	Krong A Ward, M’drak District, Dak Lak Province, Vietnam	4D + 1S	3G	U20–U23: OR978020– OR978023	OR964302– OR964306	
		*A.ingens*	YD1 to YD8, YE1 to YE4	NMNS-8764-093 to NMNS-8764-104	Ea Sup District, Dak Lak Province, Vietnam	7D + 5S	3H–I	YD1–YD8: OR978024– OR978031 YE1–YE4: OR978032– OR978035	YD1–YD2: OR964307– OR964308 YD5–8: OR964309– OR964312 YE1–YE4: OR964313– OR964316	
5	*Amphidromusingensoides* Jirapatrasilp & Lee sp. nov.	*Amphidromus* sp. 2	G4, P6	NHMUK 20230614, NMNS-8764-105	CuMta Ward, M’drak District, Dak Lak Province, Vietnam	1D + 1S	8G–H	OR978036– OR978037	OR964317– OR964318	This study
		*Amphidromus* sp. 2	U10, U11	NMNS-8764-106, NMNS-8764-107	Hon Ba, Khanh Son District, Khanh Hoa Province, Vietnam	1D + 1S	8I–J	OR978038– OR978039	OR964319– OR964320	
6	*Amphidromusbuelowi* Fruhstorfer, 1905	*A.buelowi*	SUK1 to SUK4	NMNS-8764-022 to NMNS-8764-025	Mount Singgalang, Sepuluh Koto, Tanah Datar Regency, West Sumatra, Indonesia	4D	15E	SUK2–SUK3: OR978040– OR978041	SUK2–SUK3: OR964321– OR964322	This study
		*A.asper*	VCF, VCI7	NMNS-8764-026, NMNS-8764-027	Lang-Biang plateau, Lac Duong District, Lam Dong Province, Vietnam	2D	15G	VCF: OR978042	VCF: OR964323	
		*A.franzhuberi*	VCG, VCI1 to VCI6	NMNS-8764-028 to NMNS-8764-034	Nha Trang, Khanh Hoa Province, Vietnam	6D + 1S	15H–I	VCG: OR978043 VCI1–VCI3: OR978044–OR978046	VCG: OR964324 VCI1–VCI3: OR964325– OR964327	
7	*Amphidromusthachi* Huber, 2015	*A.thachi*	VBQ1, VBQ2	NMNS-8764-264, NMNS-8764-265	Lac Duong District, Lam Dong Province, Vietnam	2D	17G–H	OR978048– OR978049	OR964329– OR964330	This study
		*A.thachi*	VBI1 to VBI4	NMNS-8764-266 to NMNS-8764-269	Vinh Thanh, Binh Dinh Province, Vietnam	3D + 1S	17D	VBI1: OR978047	VBI1: OR964328	
		*A.thachi*	VCD1, VCD2	NMNS-8764-270, NMNS-8764-271	Buon Don District, Dak Lak Province, Vietnam	1D + 1S	17E	OR978050– OR978051	OR964331– OR964332	
		*A.thachi*	VMAM	NMNS-8764-272	Da Lat, Lam Dong Province, Vietnam	1S	17F	OR978052	OR964333	
		*A.thachi*	XM1, XM2	NMNS-8764-273, NMNS-8764-274	Krong Bong, Dak Lak Province, Vietnam	2D	–	OR978053– OR978054	OR964334– OR964335	
8	*Amphidromusmetabletus* Möllendorff, 1900	*A.metabletus*	P3 to P5, XE1 to XE5	NMNS-8764-123 to NMNS-8764-130	Nha Trang, Khanh Hoa Province, Vietnam	4D + 4S	21C–F	P3–P5: OR978055– OR978057 XE1–XE5: OR978064– OR978068	P3–P5: OR964336– OR964338 XE1–XE5: OR964344–OR964348	This study
		*A.metabletus*	VMELa1 to VMELa6, VMELb1 to VMELb6, VMELc1, VMELd1 to VMELd3, VMELe1 to VMELe3	NMNS-8764-131 to NMNS-8764-149	Ninh Hoa, Khanh Hoa Province, Vietnam	15D + 4S	21G–L	VMELa6: OR978058 VMELb6: OR978059 VMELc1: OR978060 VMELd1: OR978061 VMELd3: OR978062 VMELe1: OR978063	VMELa6: OR964339 VMELb6: OR964340 VMELc1: OR964341 VMELd1: OR964342 VMELe1: OR964343	
9	*Amphidromushaematostoma* Möllendorff, 1898	*A.haematostoma*	X91 to X94	NMNS-8764-053 to NMNS-8764-056	Samphanh District, Phongsali Province, Laos	4S	24D	–	X92–X94: OR964349– OR964351	This study
		*A.haematostoma*	ZK0 to ZK9, ZK9a to ZJ9j	NMNS-8764-057 to NMNS-8764-076	Ba Chien, Pakse District, Champasak Province, Laos	20S	24E–F	ZK6–ZK7: OR978073– OR978074 ZK9: OR978075	ZK6–ZK7: OR964352– OR964353	
		*A.haematostoma*	AM36	ex. Maassen collection	Boloven Plateau, Paksong District, Champasak Province, Laos	1S	–	OR978069	–	
		*A.haematostoma*	VMDO1 to VMDO5	NMNS-8764-077 to NMNS-8764-081	Kbang District, Gia Lai Province, Vietnam	5S	24G	VMDO1: OR978070 VMDO4: OR978071 VMDO5: OR978072	–	
10	*Amphidromusmadelineae* Thach, 2020	*A.madelineae*	VBO1 to VBO5	NMNS-8764-108 to NMNS-8764-112	Duy Xuyen District, Quang Nam Province, Vietnam	5S	24I–J	VBO2: OR978076 VBO5: OR978077	VBO2: OR964354 VBO5: OR964355	This study
		*A.madelineae*	VKBG0 to VKBG9	NMNS-8764-113 to NMNS-8764-122	Za Hung, Dong Giang District, Quang Nam Province, Vietnam	10S	24K–M	VKBG1: OR978078 VKBG5: OR978079 VKBG9: OR978080	VKBG1: OR964356 VKBG5: OR964357 VKBG9: OR964358	
11	*Amphidromuscostifer* Smith, 1893	*A.costifer*	YW0 to YW9	NMNS-8764-035 to NMNS-8764-044	Tay Son District, Binh Dinh Province, Vietnam	10D	27D–E	–	YW0–YW8: OR964366– OR964374	This study
		*A.costifer*	YF1 to YF6	NMNS-8764-045 to NMNS-8764-050	Ea Sup District, Dak Lak Province, Vietnam	6D	27F–G	YF4: OR978083	YF2–YF6: OR964361– OR964365	
		*A.nguyenkhoai*	VKBE1, VKBE2	NMNS-8764-051, NMNS-8764-052	An Lao District, Binh Dinh Province, Vietnam	2D	27H–I	OR978081– OR978082	OR964359– OR964360	
12	*Amphidromuspankowskianus* Thach, 2020	*A.pankowskianus*	CAF1 to CAF3	NMNS-8764-150 to NMNS-8764-152	Khammouan Province, Laos, near Minh Hoa District, Quang Binh Province, Vietnam	2D + 1S	30K–L	CAF1–CAF2: OR978084– OR978085	CAF1: OR964375	This study
		*A.pankowskianus*	VTAR1 to VTAR40	NMNS-8764-153 to NMNS-8764-191, NMNS-8764-212	Lak Sao, Khamkeut District, Bolikhamsai Province, Laos	23D + 17S	30H–J	VTAR02: OR978086 VTAR06: OR978087 VTAR08–VTAR09: OR978088–OR978089 VTAR11: OR978090 VTAR15: OR978091	–	
13	*Amphidromusroseolabiatus* Fulton, 1896	*A.roseolabiatus*	CAB0 to CAB9	NMNS-8764-254 to NMNS-8764-263	Kambong Siem District, Kampong Cham Province, Cambodia	4D + 6S	30D–F	CAB0: OR978092 CAB2–CAB3: OR978093– OR978094 CAB7: OR978095	CAB0: OR964376 CAB2–CAB3: OR964377– OR964378 CAB7: OR964379	This study
14	*Amphidromuscruentatus* (Morelet, 1875)		X71 to X79, X81 to X88	NMNS-8476-001 to NMNS-8476-009, NMNS-8476-034 to NMNS-8476-041	Samphanh District, Phongsali Province, Laos	17S	–	OL352241–OL352248, OL352249–OL352255	X71: OL352062 X73–X79: OL352063–OL352069 X81–X88: OL352070–OL352077	Lee et al. (2022)
			ZY3, ZY4, ZY7	NMNS-8476-054, NMNS-8476-055, NMNS-8476-058	Chu Prong District, Gia Lai Province, Vietnam	3S	–	OL352256–OL352258	ZY3–ZY4: OL352078–OL352079 ZY7: OL352080	
15	*Amphidromuscontrarius* (Müller, 1774)		AM C.468733	–	6.5 km N of Los Palos, Lautem District, Timor-Leste	1S	–	KP085341	KP085031	Köhler and Criscione (2015)
16	*Amphidromusperversus* (Linnaeus, 1758)		AM19	CUMZ 4291	Bali Island, Indonesia	1S	–	MW649970	MW652850	Jirapatrasilp et al. (2022)
17	*Amphidromussinistralis* (Reeve, 1849)		AM38	ex. Maassen collection	Sulawesi, Indonesia	1S	–	OR978096	–	This study
Outgroup										
18	*Camaenacicatricosa* (Müller, 1774)		FJIQBC18503	–	Guiping, Guangxi, China	–	–	KU061276	KU586474	Ding et al. (2016)
19	*Camaenapoyuensis* Zhou, Wang & Ding, 2016		FJIQBC18484	–	Poyue town, Bama, Hechi, Guangxi, China	–	–	KU061273	KU586468	

Preliminary morphospecies identifications were based on (1) the shell characters used in the original descriptions and other relevant literature, such as Zilch (1953), Sutcharit et al. (2015), and Thach (2016, 2017, 2018, 2020a, 2021), (2) the accordance between the collecting localities and the type locality, and (3) comparisons with type specimens and/or reference collections from several natural history museums. The type localities are mentioned in the wording and language of the original descriptions. If possible, the current names and/or regional names of the type localities are provided in square brackets.

### ﻿﻿Molecular phylogenetic analyses

The acquisition of new DNA data of both mitochondrial COI and 16S rRNA, and molecular phylogenetic analyses including the calculation of intra- and interspecific *p*-distances and constructions of phylogenetic trees and haplotype networks, follow Jirapatrasilp et al. (2022) and Lee et al. (2022). New sequences were obtained from a total of 127 specimens from 14 *Amphidromus* species. In addition, sequences of *A.perversus* (Linnaeus, 1758) (type species of *Amphidromus*), *A.contrarius* (Müller, 1774) (type species of the subgenus Syndromus), and *A.cruentatus* retrieved from GenBank (Köhler and Criscione 2015; Jirapatrasilp et al. 2022; Lee et al. 2022) were included, resulting in a total of 17 *Amphidromus* species in the dataset. Sequences of *Camaenacicatricosa* (Müller, 1774) and *C.poyuensis* Zhou, Wang & Ding, 2016 from GenBank (Ding et al. 2016) were used as outgroup (Table 1).

The sequence alignments of each gene fragment were performed separately using MAFFT (v. 7, see https://mafft.cbrc.jp/alignment/server/index.html), with default options (Katoh et al. 2017). The concatenated dataset was prepared in Kakusan4 (v. 4.0.2016.11.04, see https://www.fifthdimension.jp/products/kakusan/; Tanabe 2011) with the best-fitting model adjustment for Bayesian inference (BI) analyses. The BI analysis was performed with the best-fitting models of each gene fragment and each codon position of COI (generalised time reversible (GTR) + gamma (G) for the third codon position of COI and Hasegawa–Kishono–Yano (HKY) + G for 16S rRNA and the remaining codon positions of COI) using MrBayes on XSEDE (v.3.2.6, see http://nbisweden.github.io/MrBayes/; Ronquist et al. 2012) in the CIPRES Science Gateway (see https://www.phylo.org/; Miller et al. 2010). Two independent analyses were run simultaneously and consisted of four chains of five million generations, sampling every 500 generations and discarding the first 50% of samples as burn-in.

In addition, a maximum likelihood (ML) tree was constructed using the IQ-TREE webserver (see http://iqtree.cibiv.univie.ac.at), with integrated ModelFinder function (Nguyen et al. 2015; Trifinopoulos et al. 2016; Kalyaanamoorthy et al. 2017). Branch support was estimated using 1000 ultra-fast bootstrap replicates (Hoang et al. 2018), the Shimodaira and Hasegawa-approximate likelihood-ratio (SH-aLRT) test and the approximate Bayes (aBayes) test (Anisimova et al. 2011). A clade was considered to be well supported if the ultra-fast bootstrap support (BS) values were ≥ 95%, aBayes support values ≥ 0.95, SH-aLRT support values ≥ 80% and Bayesian posterior probability values (PP) were ≥ 0.95 (San Mauro and Agorreta 2010; Anisimova et al. 2011; Hoang et al. 2018).

Uncorrected pairwise genetic distances (*p*-distances) among different *Amphidromus* species were calculated in MEGA (v. 7.0, see https://www.megasoftware.net/) using pairwise deletion (Kumar et al. 2016). Haplotype networks were constructed using the minimum spanning network method (Bandelt et al. 1999) as implemented in the program PopART (v. 1.7.2, see http://popart.otago.ac.nz/index.shtml; Leigh and Bryant 2015).

### ﻿﻿Species validation

Preliminary morphospecies identifications were validated by the reciprocal monophyly of each morphospecies in the phylogeny constructed from the concatenated COI + 16S dataset. We adopted the interspecific COI genetic distance of 4%, which has been associated with the optimum intra/interspecific threshold value for stylommatophoran land snails (Davison et al. 2009), as the threshold to validate the reciprocal monophyly of the preliminary morphospecies.

### ﻿﻿Abbreviations

The abbreviations **D** (dextral) and **S** (sinistral) are used in conjunction with numbers of specimens in the material examined sections of each species. Abbreviations for the gential organs in the figure captions follow those defined by Solem (1983) and Sutcharit and Panha (2006).

### ﻿﻿Institutional abbreviations

**ANSP** Academy of Natural Sciences of Philadelphia, Drexel University, Philadelphia

**CUMZ** Chulalongkorn University Museum of Zoology, Bangkok

**MNHN** Muséum national ďHistoire naturelle, Paris

**NHMUK** when citing specimen lots deposited in the Natural History Museum, London

**NMNS** National Museum of Natural Science of Taiwan, Taichung

**RBINS** Royal Belgian Institute of Natural Sciences, Brussels

**RMNH** Naturalis Biodiversity Center, Rijksmuseum van Natuurlijke Historie, Leiden

**SMF** Senckenberg Forschungsinstitut und Naturmuseum, Frankfurt am Main

## ﻿﻿Results

The COI dataset of *Amphidromus* in this study comprised 130 sequences with lengths between 556 and 658 bp, including 284 variable and 265 parsimony-informative sites, from an alignment length of 658 bp. The variation in the COI sequence lengths is due to incomplete sequences at both ends in some sequences. The 16S rRNA dataset comprised 118 sequences with lengths between 343 and 394 bp. The 16S rRNA alignment including gaps was 414 bp, including 136 variable and 125 parsimony-informative sites.

**Figure 2.**
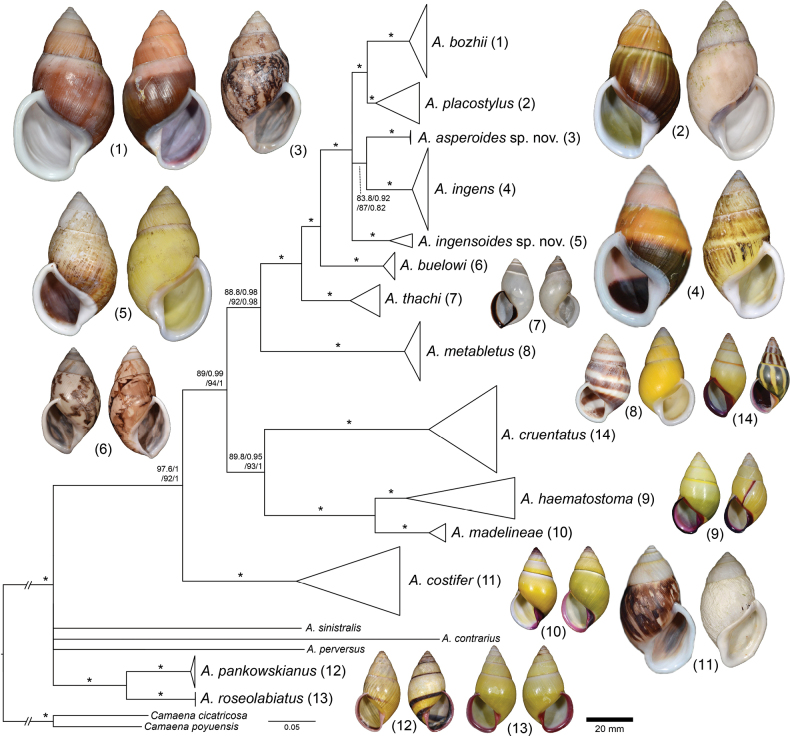
Bayesian phylogeny of *Amphidromus* spp. based on mitochondrial COI and 16S genes. Nodal support values are given as SH-aLRT/aBayes/ultra-fast bootstrap (IQ-TREE, ML)/posterior probability (MrBayes, BI). An asterisk on each branch indicates a clade with all well-supported values (SH-aLRT ≥ 80%, aBayes ≥ 0.95, BS ≥ 95%, PP ≥ 0.95). Species numbers correspond to those in Fig. 1 and Tables 1, 2.

The ML and BI phylogenetic analyses based on the concatenated datasets yielded consistent topologies (Fig. 2, showing BI topology). The preliminary morphospecies identification in this study yielded a total of 16 *Amphidromus* morphospecies (Table 1), of which 11 showed a well-supported reciprocal monophyly (SH-aLRT ≥ 80%, aBayes ≥ 0.95, BS ≥ 95%, PP ≥ 0.95) (Fig. 2), supporting their recognition as valid species. In addition, *Amphidromus* sp. 1 and sp. 2 also showed well-supported reciprocal monophyly and were characterised by a distinct shell morphology. Therefore, these latter two taxa were described as new species to science (*A.asperoides* sp. nov. and *A.ingensoides* sp. nov., respectively). Specimens previously identified as *A.asper* Haas, 1934 and *A.franzhuberi* Thach, 2016 belonged to the same clade as *A.buelowi*, and *A.nguyenkhoai* Thach, 2020 belonged to the same clade as *A.costifer*.

The DNA sequence data show that the phylogenetic relationships among the species did not mirror their geographical ties. *Amphidromuscontrarius*, *A.perversus*, and *A.sinistralis* each did not belong to the same clades of the other taxa, and the relationships of these three species with other species remain unresolved. *Amphidromusroseolabiatus* and *A.pankowskianus* were retrieved together as sister clades forming a distinct well-supported clade (SH-aLRT ≥ 80%, aBayes ≥ 0.95, BS ≥ 95%, PP ≥ 0.95) separate from the clade with the remaining taxa. These latter were grouped in a well-supported clade, with *A.costifer* as a sister taxon to all other species in this clade. *Amphidromuscruentatus*, *A.haematostoma*, and *A.madelineae* were closely related in that *A.cruentatus* was sister to the clade *A.haematostoma* + *A.madelineae*. The remaining taxa belonged to a well-supported clade, where *A.bozhii*, *A.ingens*, *A.placostylus*, as well as the two new species belonged to the same well-supported subclade. *Amphidromusbozhii* was sister to *A.placostylus*, and *A.asperoides* sp. nov. was sister to *A.ingens*.

The COI *p*-distances ranged from 0 to 10.03% (average 2.78 ± 3.04%) within species and from 9.61 to 24.16% (average 18.30 ± 3.35%) between species (Table 2). All interspecific pairwise distances exceed 9%, and 92.6% of them (126 out of 136) exceed 12%. Pairwise distances lower than 12% were observed among *A.bozhii*, *A.ingens*, *A.placostylus*, *A.asperoides* sp. nov., and *A.ingensoides* sp. nov. Intraspecific distances typically fall below or hover around 5%. Notable exceptions are *A.haematostoma* at 10.03% and *A.costifer* at 7.84%.

**Table 2.** Percentage of uncorrected pairwise interspecific distances for the partial COI (above the diagonal) and 16S rRNA (below the diagonal) gene fragments among the *Amphidromus* species in this study. Intraspecific distances for COI/16S rRNA are shown on the diagonal (bold). Species numbers correspond to those in Figs 1, 2, and Table 1. The numbers of sequences used to calculate the distances of each respective gene fragment are given as *n* = COI,16S in the first column.

Species	1	2	3	4	5	6	7	8	9	10	11	12	13	14	15	16	17
1. *A.bozhii* (*n* = 17, 4)	**0.60/ 0.13**	9.61	10.33	10.82	9.99	13.29	13.04	17.30	19.19	18.25	18.88	18.87	17.94	21.40	22.36	18.97	18.85
2. *A.placostylus* (*n* = 10, 10)	4.32	**5.47/ 3.14**	9.83	10.51	10.53	12.79	13.04	16.57	19.02	18.44	17.89	19.60	18.11	21.09	22.16	19.74	19.75
3. *A.asperoides* sp. nov. (*n* = 4, 4)	3.31	4.68	**0/0**	10.32	10.69	13.13	12.69	16.72	18.47	18.02	17.12	20.33	18.78	20.76	22.41	18.45	18.29
4. *A.ingens* (*n* = 18, 16)	2.76	4.23	3.02	**1.37/ 0.52**	10.20	12.23	12.74	15.91	19.12	18.38	17.06	20.01	18.26	20.65	20.71	17.71	20.01
5. *A.ingensoides* sp. nov. (*n* = 4, 4)	4.25	5.14	4.61	4.19	**3.32/ 1.45**	13.51	12.80	16.27	19.16	19.48	17.63	19.98	19.20	21.76	21.62	18.18	19.23
6. *A.buelowi* (*n* = 7, 7)	5.00	5.60	4.61	4.61	4.89	**1.25/ 0.19**	13.40	17.05	18.72	18.79	18.47	20.63	18.32	21.01	21.78	18.75	19.04
7. *A.thachi* (*n* = 8, 8)	7.41	8.21	6.22	7.19	6.90	6.36	**2.21/ 1.07**	16.32	19.81	19.43	16.81	19.67	18.31	20.81	21.03	18.05	19.28
8. *A.metabletus* (*n* = 14, 13)	11.08	12.11	11.67	12.04	10.84	11.40	10.68	**1.28/ 0.58**	20.40	19.43	16.63	20.47	18.53	20.56	21.53	18.30	19.25
9. *A.haematostoma* (*n* = 7, 5)	10.53	10.80	10.10	9.59	9.66	9.38	10.52	12.59	**10.03/ 1.99**	13.93	20.61	22.44	21.43	20.76	23.28	21.39	21.74
10. *A.madelineae* (*n* = 5, 5)	9.71	9.89	9.17	8.10	8.78	9.26	9.66	11.63	6.04	**2.19/ 0.33**	17.81	21.12	19.61	20.26	22.90	21.62	20.60
11. *A.costifer* (*n* = 3, 16)	15.45	15.56	14.85	15.33	14.91	13.44	13.69	14.41	16.00	16.02	**7.84/ 3.39**	19.53	19.31	20.38	21.73	20.23	18.90
12. *A.pankowskianus* (*n* = 8, 1)	13.72	14.18	14.20	13.44	13.61	12.50	14.31	13.78	14.01	13.15	14.37	**0.19/NA**	13.02	20.49	21.57	19.59	20.38
13. *A.roseolabiatus* (*n* = 4, 4)	14.28	14.21	13.91	14.09	15.10	13.23	13.03	15.51	14.56	13.08	15.31	6.14	**0/0**	20.16	21.93	18.97	19.08
14. *A.cruentatus* (*n* = 18, 19)	10.17	9.93	10.82	9.71	9.90	10.26	11.11	12.04	11.37	12.17	15.39	14.21	15.63	**3.15/ 1.17**	24.16	20.13	21.37
15. *A.contrarius* (*n* = 1, 1)	15.62	16.12	15.90	16.04	15.56	15.41	15.57	16.74	16.41	15.16	14.91	14.29	14.91	16.11	**NA/NA**	21.65	21.95
16. *A.perversus* (*n* = 1, 1)	13.75	14.08	14.00	13.44	14.51	14.70	14.93	16.18	15.89	13.09	13.86	13.61	13.09	15.46	13.45	**NA/NA**	18.29
17. *A.sinistralis* (*n* = 1, 0)	NA	NA	NA	NA	NA	NA	NA	NA	NA	NA	NA	NA	NA	NA	NA	NA	**NA/NA**

Comparable patterns were observed for 16S, the *p*-distances of which ranged from 0 to 3.39% (average 1.07 ± 1.14%) within species and from 2.76–16.74% (average 11.68 ± 3.79%) between species (Table 2). All interspecific pairwise distances exceed 3%, except between *A.bozhii* and *A.ingens* (2.76%), and 90% of the interspecific *p*-distances (108 out of 120) exceed 5%. Intraspecific distances typically fall below or hover around 3%.

### ﻿﻿Systematics

﻿**Family Camaenidae Pilsbry, 1895**

#### ﻿﻿Genus Amphidromus

Taxon classificationAnimaliaStylommatophoraCamaenidae

Albers, 1850

E2447A08-6AB4-59DB-8AEA-24A40028A8BB

Amphidromus Albers, 1850: 138. Martens in Albers 1860: 184. Fulton 1896: 66, 94.

##### Type species.

*Helixperversus* Linnaeus, 1758 by subsequent designation of E. von Martens in Albers (1860).

#### ﻿﻿ Amphidromus ingens

Taxon classificationAnimaliaStylommatophoraCamaenidae

Möllendorff, 1900

9306C6D6-F206-550B-A921-97BD95C518F4

**Figure 3.**
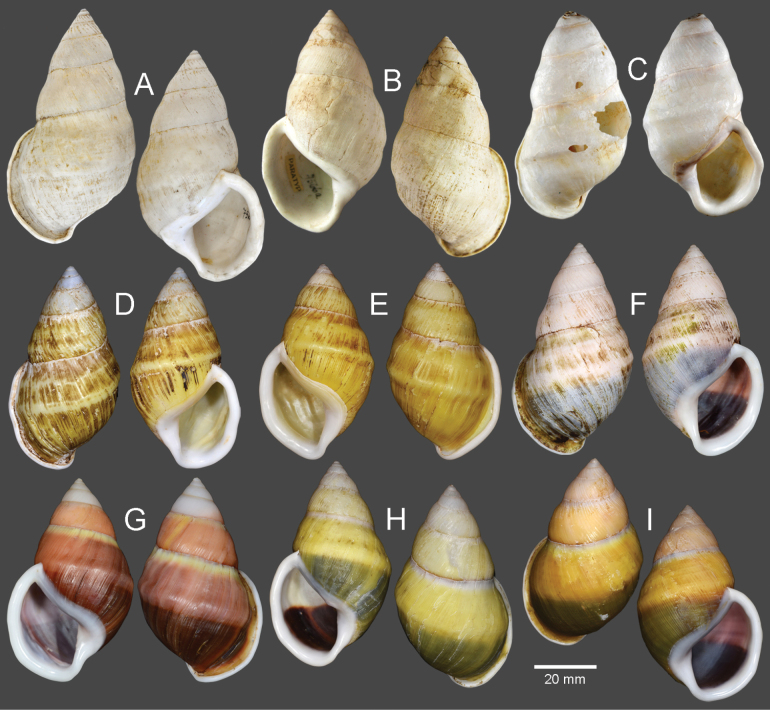
Shells of *Amphidromusingens* Möllendorff, 1900 **A** lectotype of “*Amphidromusingens*” (SMF 7565) **B** paralectotype of “*Amphidromusingens*” (SMF 7566) **C** holotype of “*Amphidromusnaggsi*” (RMNH.5003908) **D, E** specimens from M’drak, Dak Lak, Vietnam (NMNS-8764-082, NMNS-8764-084) **F** specimen from Ea M’doal, M’drak, Dak Lak, Vietnam (NMNS-8764-087) **G** specimen from Krong A, M’drak, Dak Lak, Vietnam (NMNS-8764-088) **H, I** specimens from Ea Sup, Dak Lak, Vietnam (NMNS-8764-093, NMNS-8764-101). Credit: J. Goud, RMNH (**C**).

**Figure 4.**
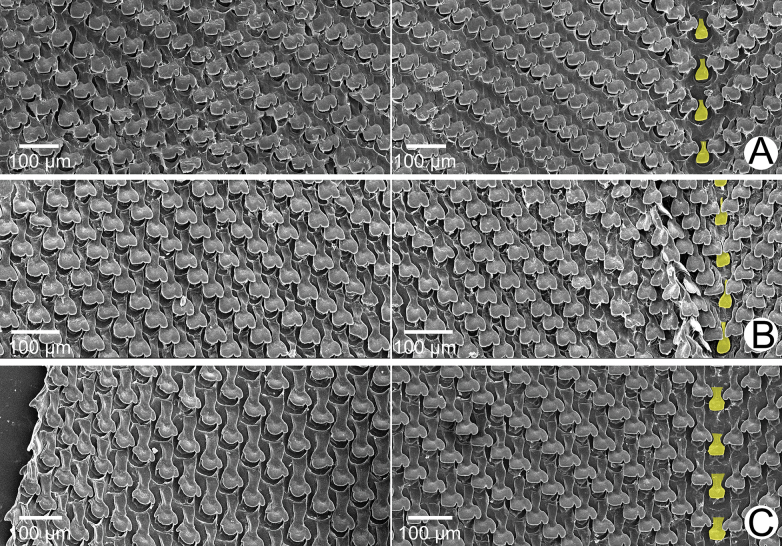
SEM images of the radula of *Amphidromus* spp **A***Amphidromusingens* Möllendorff, 1900 from Ea Sup, Dak Lak, Vietnam (NMNS-8764-100) **B***Amphidromusasperoides* sp. nov. from Ea Tu, Buon Ma Thuat city, Dak Lak, Vietnam (NMNS-8764-001) **C***Amphidromusbozhii* Wang, 2019 from Tuy Hoa, Phu Yen, Vietnam (NMNS-8764-016). Central teeth are marked in yellow. The left and right images show the outer and inner sections of each radula, respectively.

**Figure 5.**
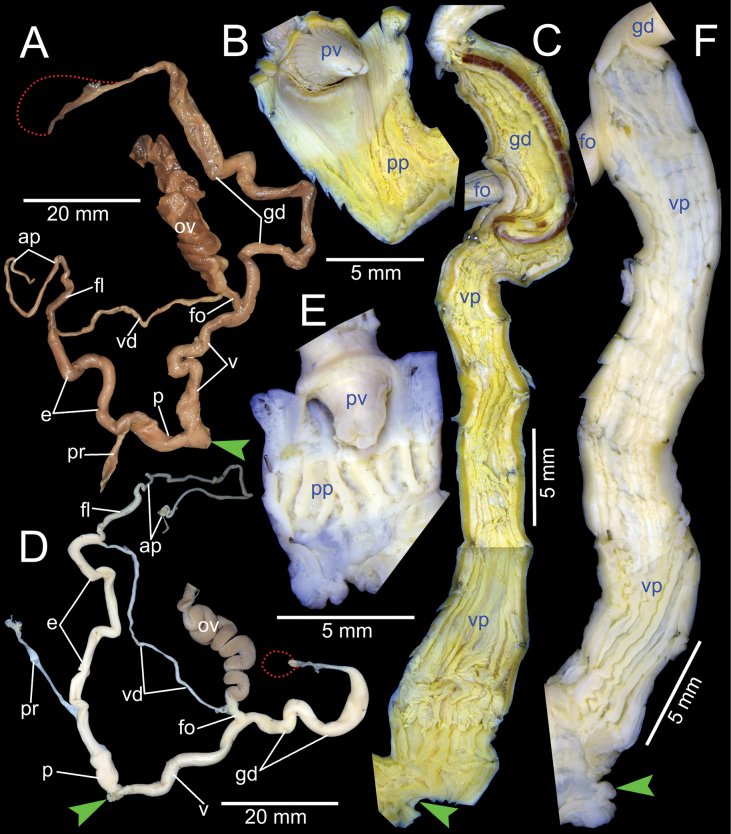
Genitalia of *Amphidromus* spp **A–C***Amphidromusingens* Möllendorff, 1900 from Ea Sup, Dak Lak, Vietnam (NMNS-8764-100), showing **A** general view of genitalia **B** interior structures of penis **C** interior structures of vagina chamber and gametolytic duct **D–F***Amphidromusasperoides* sp. nov. from Ea Tu, Buon Ma Thuat city, Dak Lak, Vietnam (NMNS-8764-001), showing **D** general view of genitalia **E** interior structures of penis **F** interior structures of vagina chamber. Red dots indicate the shape of the missing gametolytic sac. Green arrows indicate the genital openings. Abbreviations: ap, appendix; e, epiphallus; fl, ﬂagellum; fo, free oviduct; gd, gametolytic duct; ov, oviduct; p, penis; pp, penial pilaster; pr, penial retractor muscle; pv, penial verge; v, vagina; vd, vas deferens; vp, vaginal pilaster

**Figure 6.**
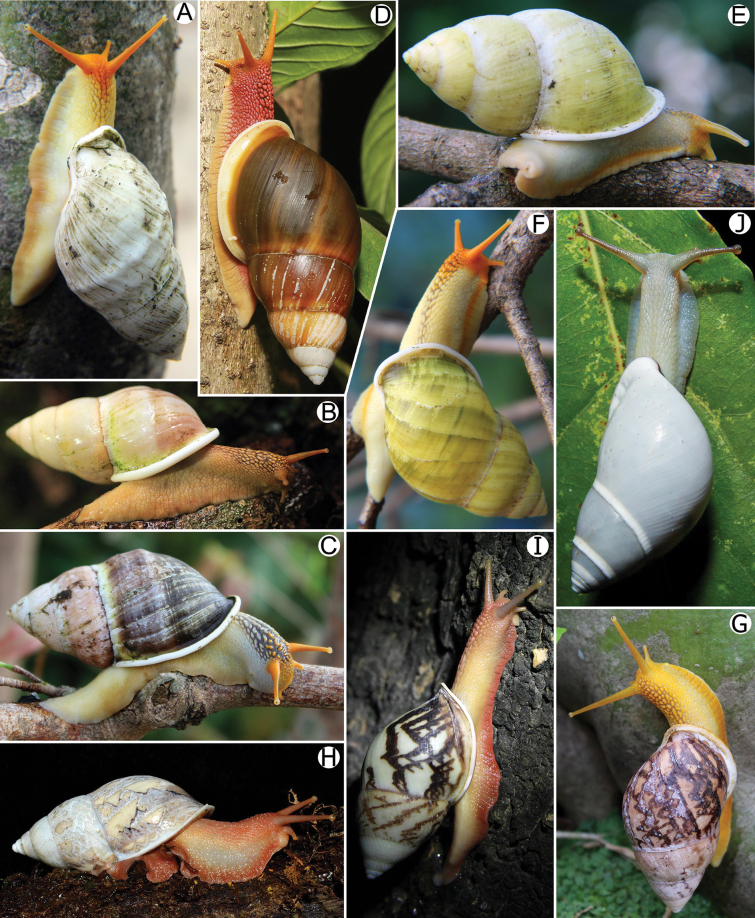
Living *Amphidromus* spp **A–C***Amphidromusingens* Möllendorff, 1900 from Dak Lak, Vietnam **D***Amphidromusplacostylus* Möllendorff, 1900 from Hoai An, An Lao, Binh Dinh, Vietnam **E, F***Amphidromusingensoides* sp. nov. from Hon Ba, Khanh Son, Khanh Hoa, Vietnam **G***Amphidromusasperoides* sp. nov. from Ea Tu, Buon Ma Thuat city, Dak Lak, Vietnam **H, I***Amphidromusbuelowi* Fruhstorfer, 1905 from Lang-Biang plateau, Lac Duong, Lam Dong, Vietnam **J***Amphidromusthachi* Huber, 2015 from Krong Bong, Dak Lak, Vietnam.

**Figure 7.**
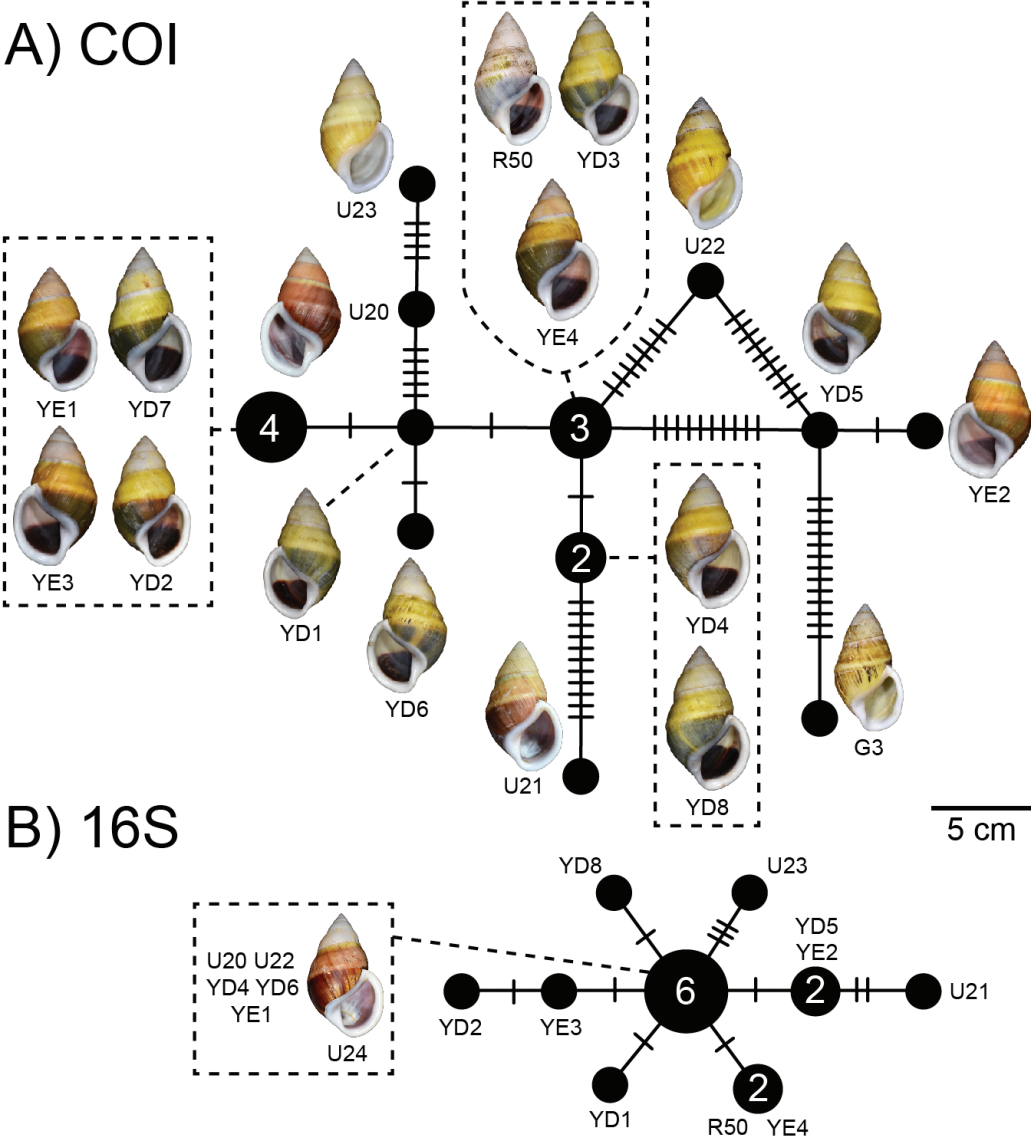
Mitochondrial haplotype minimum spanning networks of *Amphidromusingens* Möllendorff, 1900 **A** COI and **B** 16S rRNA. The size of each circle corresponds to the frequency of that haplotype, also shown as the number in that circle. The bars on the branches indicate the number of mutational steps between haplotypes. Specimen codes correspond to those in Table 1.

Figs 3 , 4A , 5A–C , 6A–C , 7

Amphidromus ingens Möllendorff, 1900b: 23–24. Type locality: Berg “Mutter und Kind”, Annam [Vietnam]. Pilsbry 1900: 175–176. Fischer and Dautzenberg 1904: 406. Laidlaw and Solem 1961: 529, 629. Richardson 1985: 21. Thach 2005: 235, pl. 73, fig. 22. Schileyko 2011: 50. Sutcharit et al. 2021: fig. 1g.
Amphidromus (Amphidromus) ingens. Zilch 1953: 135, pl. 23, fig. 25.
Amphidromus naggsi Thach & Huber, 2014: 35–37, figs 1–13, 15. Type locality: Don Duong district, Lam Dong Province, South Vietnam. Páll-Gergely et al. 2020: 53. Thach et al. 2020: 185, 187, pl. 1, fig. 6a. Thach 2020a: 70, fig. 881 left. Thach 2021: 70. syn. nov.

##### Material examined.

Vietnam: Dextral, ***lectotype*** of “*Amphidromusingens*”, SMF 7565/1 (Fig. 3A); 2D + 2S, paralectotypes of “*Amphidromusingens*”, SMF 7566/4 (Fig. 3B). Vietnam: Dextral, ***holotype*** of “*Amphidromusnaggsi*”, RMNH.5003908 (Fig. 3C).

##### Other material examined.

Vietnam: 4D + 1S specimens, M’drak District, Dak Lak Province, NMNS-8764-082–NMNS-8764-086 (Fig. 3D, E); 1D specimen, Ea M’doal ward, M’drak District, Dak Lak Province, NMNS-8764-087 (Fig. 3F); 4D + 1S specimens, Krong A ward, M’drak District, Dak Lak Province, NMNS-8764-088–NMNS-8764-092 (Fig. 3G); 7D + 5S specimens, Ea Sup District, Dak Lak Province, NMNS-8764-093–NMNS-8764-104 (Fig. 3H, I).

##### Diagnosis.

Shell large conical and chirally dimorphic (sinistral and dextral coiling). Shell surface with coarse growth lines; last whorl with subsutural depression area and more or less prominent keel on periphery. Genitalia with appendix.

##### Differential diagnosis.

*Amphidromusingens* is unique among all reported Vietnamese species (Schileyko 2011) in having a last whorl with subsutural depression area and more or less prominent keel on periphery. *Amphidromusbozhii* is similar in most of the shell form and sculpture, but the shell sculpture of *A.bozhii* has a very weak spiral depression area and sometimes with or without keel, and the shell colour is generally rose-pink to dark colour, with last whorl stained with dark brown colour below periphery and ~ 1/2 of upper periphery. On the other hand, *A.ingens* has a monochrome (whitish, yellowish, tinted pink) shell, often stained with dark brown to blackish below periphery. *Amphidromusingens* is also recognised by a distinct clade in the molecular phylogeny (Fig. 2), with the closest *p*-distance to *A.ingensoides* sp. nov. in COI (10.2%) and *A.bozhii* in 16S (2.76%) (Table 2).

##### Description.

***Shell*** large (height 62.3–74.6 mm, width 38.5–42.5 mm), chirally dimorphic, solid, and ovate conical shape. Spire long conical to elongate conical, apex acute without black spot on tip. Whorls 5–7 convex; suture wide and depressed; last whorl rounded to slightly angulated. Periostracum brownish to thin corneous; varix usually absent. Shell surface generally with irregular and coarse growth lines; below sutural with broad subsutural depression area, and with blunt or low to prominent keel on periphery. Shell colour highly variable: monochrome (whitish, yellowish, tinted pink) to stained with dark brown to blackish below periphery. Parietal callus thickened and white, dilated at umbilical area. Aperture broadly ovate; inner side of outer wall with white, yellow or dark brown to blackish colour. Peristome thickened, expanded, and reflexed but not attached to last whorl; lip whitish. Columella white, straight, or little twisted. Umbilicus imperforate.

***Radula*.** Teeth arranged in anteriorly pointed V-shaped rows. Central tooth monocuspid and spatulate with truncated cusp. Lateral teeth bicuspid; endocone small, slightly curved, with wide notch and dull cusp; ectocone large with truncated to slightly curved cusp. Lateral teeth gradually transformed to asymmetric tricuspid marginal teeth (Fig. 4A).

***Genital organs*.** Atrium relatively short. Penis slender, conical, and short ~ 1/3 of vaginal length. Penial retractor muscle thickened and inserting on epiphallus close to penis. Epiphallus long, slender tube, slightly narrower than penis. Flagellum short ~ 1/2 of epiphallus and terminating in slightly enlarged folded coil. Appendix short, slender tube, approximately as long as epiphallus, and ~ 2× longer than flagellum. Vas deferens slender tube passing from free oviduct and terminating at epiphallus-flagellum junction (Fig. 5A). Internal wall of penis corrugated, exhibiting series of thickened and swollen longitudinal penial pilasters forming fringe around penial wall, and with nearly smooth to weak folds around base of penial verge. Penial verge short conical with nearly smooth surface, and with opening on the tip (Fig. 5B).

Vagina slender, long cylindrical, and ~ 3× longer than penis. Gametolytic duct enlarged cylindrical tube then abruptly tapering to slender tube terminally and connected to gametolytic sac (missing during dissecting). Free oviduct short; oviduct compact and enlarged to form lobule alveoli (Fig. 5A). Internal wall of vagina possessing corrugated ridges near genital orifice; ridges becoming thinner and smooth surfaced longitudinal vaginal pilasters, swollen with irregularly shaped and deep crenelations close to free oviduct opening. Spermatophore (in part) dark brown stuck inside gametolytic duct (Fig. 5C).

***Living specimens*** generally with pale brown to yellowish body covered with reticulated skin. Foot broad and long with uniform pale brownish to yellowish colour to posterior tail. Dorsal side of anterior body usually with stripe of darkly reticulated skin; head area at base and just behind upper tentacle with orange patch. Upper tentacles drumstick shaped, orange to paler and with dark eyespots on tentacular tips; lower tentacles short and pale orange in colour (Fig. 6A–C).

##### Haplotype network.

There was a total of 12 COI haplotypes (Fig. 7A) and nine 16S haplotypes (Fig. 7B) of *A.ingens* in this study, and the highest numbers of mutational steps in the COI and 16S minimum spanning networks are 13 and three, respectively.

##### Distribution.

The distribution range of the species covers Dak Lak and Lam Dong provinces, Vietnam.

##### Remarks.

Thach and Huber (2014) introduced *A.naggsi*, which is described to differ from *A.ingens* in its wrinkled outer surface, the presence of 2–3 broad spiral channels on the body whorl, the more prominent sculpture on the penultimate whorl, and a more elongate aperture. However, upon examining the type specimens of both *A.ingens* and *A.naggsi*, these diagnostic characters were also present in the lectotype and paralectotypes of *A.ingens*, and the holotype of *A.naggsi* agrees well with all the type specimens of *A.ingens* in terms of shell shape, shell surface sculpture, peristome, and apertural shape. Thus, *A.naggsi* is regarded herein as a junior subjective synonym of *A.ingens*.

The shell colour generally varies from whitish (typical) to yellowish to rose-pink colour (Fig. 3). In our examined specimens, many are stained with dark brown colour below periphery and some are stained nearly entirely on the last and penultimate whorl. The shell sculpture generally has two depression areas, one upper periphery and one below suture, and the conspicuous keel to weak keel is generally present on periphery.

#### ﻿﻿ Amphidromus asperoides

Taxon classificationAnimaliaStylommatophoraCamaenidae

Jirapatrasilp & Lee sp. nov.

C90FD229-3422-5657-AB4D-AB3D92B8106F

https://zoobank.org/BB0A2CF5-B568-4CA7-AE3F-59C38CC082E5

**Figure 8.**
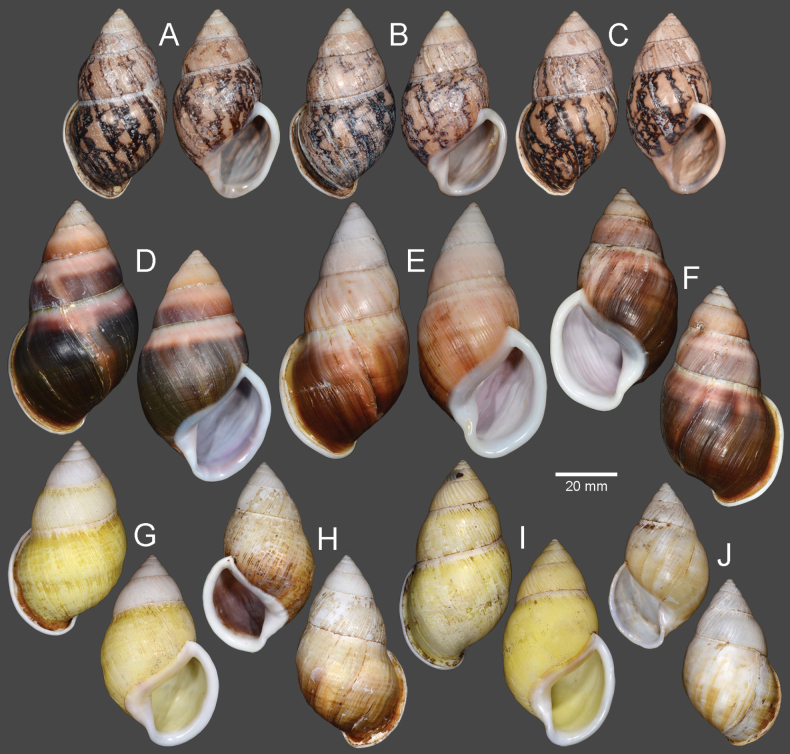
Shells of *Amphidromus* spp **A–C***Amphidromusasperoides* sp. nov. from Ea Tu, Buon Ma Thuat city, Dak Lak, Vietnam **A** holotype (NMNS-8764-001) **B, C** paratypes (NMNS-8764-002 and NHMUK 20230613) **D–F***Amphidromusbozhii* Wang, 2019 **D, E** specimens from Phu Hoa, Phu Yen, Vietnam (NMNS-8764-009, NMNS-8764-013) **F** specimen from Tuy Hoa, Phu Yen Vietnam (NMNS-8764-014) **G–J***Amphidromusingensoides* sp. nov **G** holotype from Cu’Mta, Mdrak, Dak Lak, Vietnam (NMNS-8764-106) **H** paratype from Cu’Mta, Mdrak, Dak Lak, Vietnam (NHMUK 20230614) **I, J** paratypes from Hon Ba, Khanh Son, Khanh Hoa, Vietnam (NMNS-8764-107, NMNS-8764-106).

Figs 4B , 5D–F , 6G , 8A–C

Amphidromus asper [non Haas]. Thach 2017: 37, pl. 34, figs 432, 433.

##### Diagnosis.

Shell large conical and dextral. Shell colour with dark triangular blotches connected with dark zigzag radial streaks. Aperture ovate and rounded anteriorly, columella straight. Genitalia with appendix.

##### Differential diagnosis.

The new species differs from the similar species *A.buelowi* in being exclusively dextral, having a straight columella, and lacking an apertural notch and umbilical hump. In contrast, *A.buelowi* is chirally dimorphic, and possesses a distinct twisted columella plait, a prominent umbilical hump encircled columellar area, and an apertural notch projecting anteriorly. In addition, on the soft body of living snail, *A.asperoides* sp. nov. has a uniform brownish yellow to pale brown of the entire body, while *A.buelowi* exhibits a reddish orange body. This new species is also recognised as a distinct clade in the molecular phylogeny (Fig. 2), with the closest *p*-distance to *A.placostylus* in COI (9.83%) and *A.ingens* in 16S (3.02%) (Table 2).

##### Etymology.

The specific epithet *asperoides* is from *asper*, and the suffix ‘–*oideus*’, meaning ‘like or resembling’. This name refers to the resemblance in shell morphology of the new species to the nominal species *A.asper*, which is now treated as a junior synonym of *A.buelowi*.

##### Type material.

***Holotype*.** Vietnam: dextral, shell height 61.7 mm, shell width 34.9 mm, with 7 whorls, 15 July 2016, coll. A. N. Pham (NMNS-8764-001, Fig. 8A). ***Paratypes*.** Vietnam: 2D specimens, same collecting data as holotype (NMNS-8764-001–NMNS-8764-003, Fig. 8B); 1D specimen, same collecting data as holotype (NHMUK 20230613, Fig. 8C).

##### Type locality.

Vietnam: Ea Tu village, Buon Ma Thuat city, Dak Lak Province, 12°42'24.4"N, 108°07'25.3"E.

##### Other material.

Vietnam: 20D specimens, Krong Pak, Dak Lak Province, NMNS-8764-192–NMNS-8764-211, 6 Oct. 2022, coll. V. V. Hoang.

##### Description.

***Shell*** large (height 54.6–61.7 mm, width 31.3–34.9 mm), dextral, solid, and ovate conical shape. Spire long conical with white or pale colour; apex acute without black spot on tip. Whorls 6–7 convex; suture wide and depressed; last whorl ovate. Periostracum thin corneous; varices generally present. Shell surface generally with coarse growth lines. Shell ground colour pale pink, decorated with dark triangular blotches connected with dark zigzag radial streaks. Parietal callus thickened, slightly opaque, white and much thinner in central area. Aperture ovate; without (or very weak) anterior notch and umbilical hump; inner side of outer wall whitish colour; peristome thickened, expanded, and reflexed but not attached to last whorl; lip whitish. Columella white, straight, or weakly twisted. Umbilicus imperforate.

***Radula*.** Teeth arranged in anteriorly pointed V-shaped rows. Central tooth monocuspid and spatulate with truncated cusp. Lateral teeth bicuspid; endocone small, with shallow notch and blunt cusp; ectocone large with curved cusp. Lateral teeth gradually transformed to asymmetric tricuspid marginal teeth (Fig. 4B).

***Genital organs*.** Atrium relatively short. Penis slender, conical, and short ~ 1/4 of vaginal length. Penial retractor muscle thickened, long and inserting on epiphallus near penis. Epiphallus very long ~ 2× longer than vagina, and slender tube. Flagellum short, extending from epiphallus and terminating in slightly enlarged tube. Appendix short and slender tube, 4×longer than flagellum and approximately as long as epiphallus. Vas deferens slender tube passing from free oviduct and terminating at epiphallus-flagellum junction (Fig. 5D). Internal wall of penis corrugated, exhibiting series of thickened and swollen longitudinal penial pilasters forming fringe around penial wall, and with nearly smooth to weak folds around base of penial verge. Penial verge short conical with nearly smooth surface, and with opening on the tip (Fig. 5E).

Vagina long, slender, cylindrical, and ~ 2× longer than penis. Gametolytic duct enlarged cylindrical tube then abruptly tapering to slender tube terminally and connected to gametolytic sac (missing during dissection). Free oviduct short; oviduct compact, enlarged to form lobule alveoli (Fig. 5D). Internal wall of vagina possessing corrugated ridges near genital orifice; ridges becoming thinner and smooth surfaced longitudinal vaginal pilasters, swollen with irregularly shaped shallow crenelations close to free oviduct opening (Fig. 5F).

***Living specimens*** with soft body morphology generally similar to *A.ingens*. Animals with uniform brownish yellow to pale brown of the entire body including foot, upper and lower tentacles (Fig. 6G).

##### Distribution.

This species is known from Dak Lak Province, Vietnam.

##### Remarks.

This new species had been previously identified as *A.asper* in Thach (2017). However, based on the difference in shell size and apertural characteristics to the holotype of *A.asper*, those specimens featured in Thach (2017) should be regarded as *A.asperoides* sp. nov. See also under the remarks of *A.buelowi*.

#### ﻿﻿ Amphidromus bozhii

Taxon classificationAnimaliaStylommatophoraCamaenidae

Wang, 2019

D278E27D-1E49-5BBC-9413-1F612643B99A

**Figure 9.**
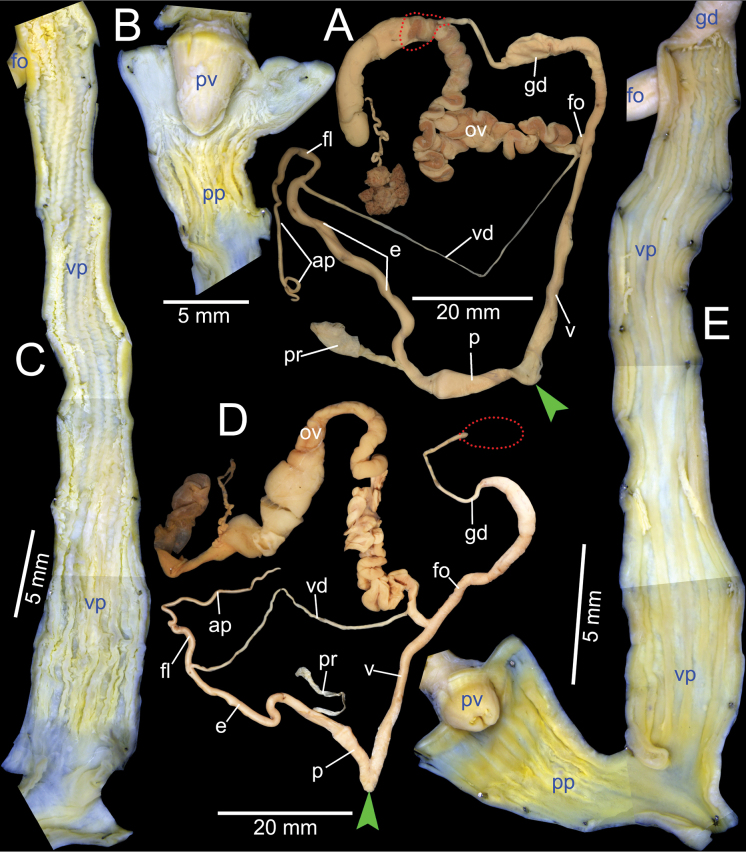
Genitalia of *Amphidromus* spp **A–C***Amphidromusbozhii* Wang, 2019 from Tuy Hoa, Phu Yen, Vietnam (NMNS-8764-016), showing **A** general view of genitalia **B** interior structures of penis **C** Interior structures of vagina chamber **D, E***Amphidromusplacostylus* Möllendorff, 1900 from Dak Po, Gia Lai, Vietnam (NMNS-8764-217), showing **D** general view of genitalia **E** interior structures of penis and vagina chamber. Red dots indicate the shape of the missing gametolytic sac. Green arrows indicate the genital openings. Abbreviations: ap, appendix; e, epiphallus; fl, ﬂagellum; fo, free oviduct; gd, gametolytic duct; ov, oviduct; p, penis; pp, penial pilaster; pr, penial retractor muscle; pv, penial verge; v, vagina; vd, vas deferens; vp, vaginal pilaster

**Figure 10.**
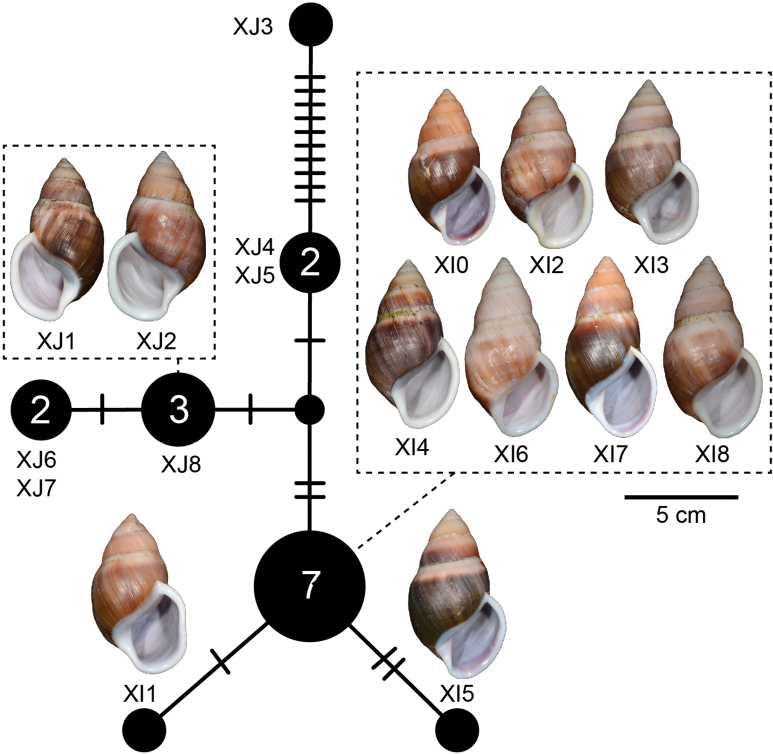
Mitochondrial COI haplotype minimum spanning networks of *Amphidromusbozhii* Wang, 2019. The size of each circle corresponds to the frequency of that haplotype, also shown as the number in that circle. The bars on the branches indicate the number of mutational steps between haplotypes. Specimen codes correspond to those in Table 1.

Figs 4C , 8D–F , 9A–C , 10

Amphidromus bozhii Wang, 2019: 300–301, pl. 3, figs a, b. Type locality: Phu Yen Province, Vietnam.

##### Material examined.

Vietnam: 10D specimens, Phu Hoa District, Phu Yen Province, NMNS-8764-004–NMNS-8764-013 (Fig. 8D, E); 8S specimens, Tuy Hoa District, Phu Yen Province, NMNS-8764-014–NMNS-8764-021 (Fig. 8F).

##### Diagnosis.

Shell large conical and chirally dimorphic. Shell surface with coarse growth lines; last whorl nearly absent of spiral depression area and keel. Genitalia with appendix.

##### Differential diagnosis.

This species is similar to *A.ingens* in most of the shell form and sculpture. The distinguishing characters are the shell colour which is generally rose-pink to dark colour. The last whorl is stained with dark brown colour below periphery and ~ 1/2 of upper periphery. The shell sculpture has a very weak spiral depression area and sometimes with or without keel. This species looks like an intermediate form between *A.ingens* and *A.placostylus*. *Amphidromusbozhii* is also recognised by a distinct clade in the molecular phylogeny (Fig. 2), with the closest *p*-distance to *A.placostylus* in COI (9.61%) and *A.ingens* in 16S (2.76%) (Table 2).

##### Description.

***Shell*** large (height 69.1–82.9 mm, width 38.3–42.0 mm), chirally dimorphic, solid, and ovate conical shape. Spire elongate conical with pale colour; apex acute without black spot on tip. Whorls 5–7 convex; suture wide and depressed; last whorl ovate. Periostracum brownish to thin corneous. Shell surface generally with irregular and coarse growth lines; very weak to nearly absent of spiral depression area and keel. Shell colour generally rose-pink to stained with dark brown colour below and ~ 1/2 of upper periphery. Parietal callus thickened, white, and dilated at umbilical area. Aperture broadly ovate; inner side of outer wall with yellow or pale brown colour; peristome thickened, expanded, and reflexed but not attached to last whorl; lip whitish. Columella white, straight, or little twisted. Umbilicus imperforate.

***Radula*.** Teeth arranged in anteriorly pointed V-shaped rows. Central tooth monocuspid and short-spatulate with truncated cusp. Lateral teeth bicuspid; endocone small, with wide notch and slightly curved and dull cusp; ectocone large with truncated to blunt cusp. Lateral teeth gradually transformed to asymmetric tricuspid marginal teeth. Outermost teeth with small and curved cusp on endocone and ectocone; mesocone large, with curved cusps (Fig. 4C).

***Genital organs*.** Atrium very short. Penis slender, conical, and short ~ 1/2 of vaginal length. Penial retractor muscle thickened and inserting on epiphallus close to penis. Epiphallus long and slender tube. Flagellum short, extending from epiphallus and terminating in slightly enlarged tube. Appendix short, slender tube, approximately as long as flagellum, and ~ 1/3 of epiphallus length. Vas deferens slender tube passing from free oviduct and terminating at epiphallus-flagellum junction (Fig. 9A). Internal wall of penis corrugated, exhibiting series of swollen longitudinal penial pilasters forming fringe around penial wall, and with nearly smooth to weak folds around base of penial verge. Penial verge conical with nearly smooth surface (Fig. 9B).

Vagina slender, long cylindrical, ~ 2× longer than penis. Gametolytic duct enlarged cylindrical tube then abruptly tapering to slender tube terminally and connected to gametolytic sac (missing during dissection). Free oviduct short; oviduct compact, enlarged to form lobule alveoli (Fig. 9A). Internal wall of vagina possesses strong corrugated ridges near genital orifice, ridges become weaker corrugated vaginal pilasters, and swollen with irregularly shaped deep crenelations close to free oviduct opening (Fig. 9C).

##### Haplotype network.

There were seven COI haplotypes of *A.bozhii* in this study, and the highest number of mutational steps in the COI minimum spanning network is ten (Fig. 10).

##### Distribution.

This species is found in Phu Yen Province, Vietnam.

#### ﻿﻿ Amphidromus placostylus

Taxon classificationAnimaliaStylommatophoraCamaenidae

Möllendorff, 1900

EB923616-0B0C-5426-A152-84E664E7EEC3

**Figure 11.**
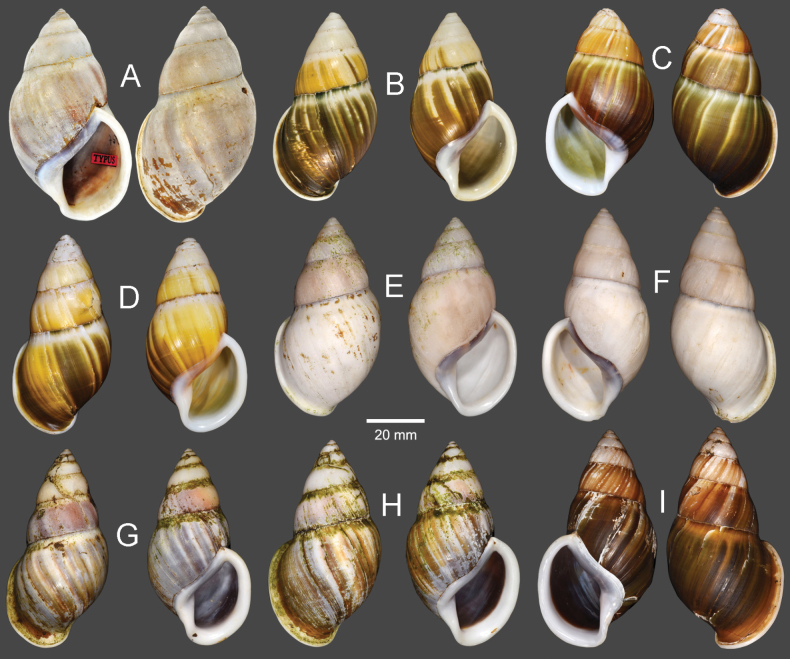
Shells of *Amphidromusplacostylus* Möllendorff, 1900 **A** lectotype of “*Amphidromusplacostylus*” (SMF 7593) **B** holotype of “*Amphidromusjohnstanisici*” (MNHN-IM-2000-33218) **C, D** specimens from Dak Po, Gia Lai, Vietnam (NMNS-8764-213, NMNS-8764-215) **E, F** specimens from Kbang, Gia Lai, Vietnam (NMNS-8764-219, NMNS-8764-221) **G–I** specimens from Hoai An, An Lao, Binh Dinh, Vietnam (NMNS-8764-222, NMNS-8764-227, NMNS-8764-231). Credit: M Caballer, MNHN (**B**).

**Figure 12.**
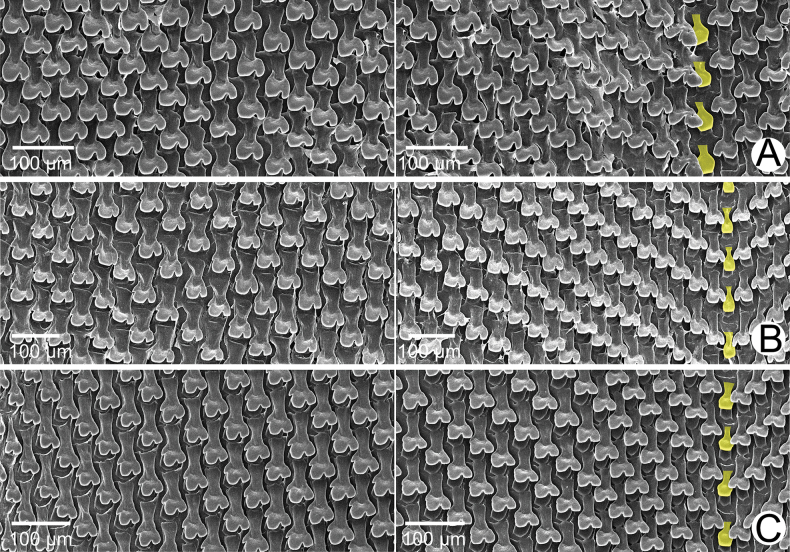
SEM images of the radula **A***Amphidromusplacostylus* Möllendorff, 1900 from Dak Po, Gia Lai, Vietnam (NMNS-8764-217) **B***Amphidromusbuelowi* Fruhstorfer, 1905 from Mount Singgalang, Sepuluh Koto, Tanah Datar Regency, West Sumatra, Indonesia (NMNS-8764-024) **C***Amphidromusthachi* Huber, 2015 from Buon Don, Dak Lak, Vietnam (NMNS-8764-271). Central teeth are marked in yellow. The left and right images show the outer and inner sections of each radula, respectively.

**Figure 13.**
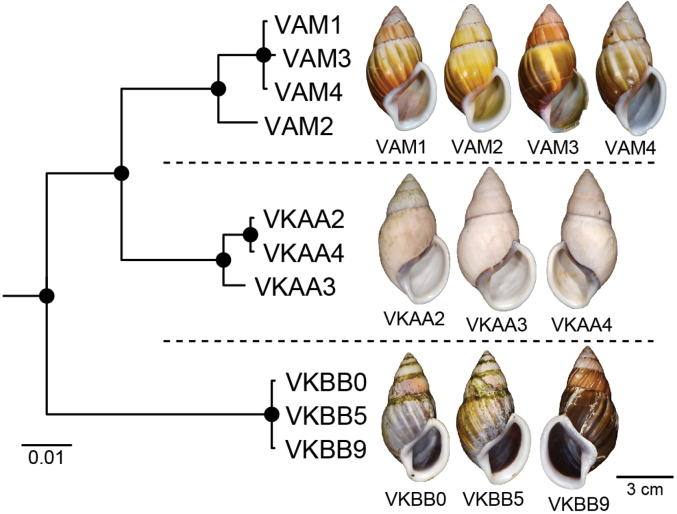
Bayesian phylogeny of *Amphidromusplacostylus* Möllendorff, 1900 based on mitochondrial COI and 16S genes. Nodal support values are given as SH-aLRT/aBayes/ultra-fast bootstrap (IQ-TREE, ML)/posterior probability (MrBayes, BI). An asterisk on each branch indicates a clade with all well-supported values (SH-aLRT ≥ 80%, aBayes ≥ 0.95, BS ≥ 95%, PP ≥ 0.95).

Figs 6D , 9D, E , 11 , 12A , 13

Amphidromus placostylus Möllendorff, 1900a: 132. Type locality: Phuc-son [Phuc Son Commune, Tan Yen District, Bac Giang Province, Vietnam]. Pilsbry 1900: 178. Fischer and Dautzenberg 1904: 406. Laidlaw and Solem 1961: 529, 649–650. Richardson 1985: 38. Schileyko 2011: 51. Sutcharit et al. 2021: fig. 1f.
Amphidromus (Amphidromus) placostylus. Zilch 1953: 138, pl. 25, fig. 41.
Amphidromus johnstanisici Thach & Huber in Thach, 2017: 41, pl. 53, figs 657–663. Type locality: Kbang District, Gia Lai Province, Central Vietnam. Thach 2021: 65. syn. nov.

##### Material examined.

Vietnam: Dextral, ***lectotype*** of “*Amphidromusplacostylus*”, SMF 7593 (Fig. 11A); dextral, ***holotype*** of “*Amphidromusjohnstanisici*”, MNHN-IM-2000-33218 (Fig. 11B).

##### Other material examined.

Vietnam: 4D + 1S specimens, Dak Po District, Gia Lai Province, NMNS-8764-213–NMNS-8764-217 (Fig. 11C, D); 3D + 1S specimens, Kbang, Gia Lai Province, NMNS-8764-218–NMNS-8764-221 (Fig. 11E, F); 4D + 6S specimens, Hoai An, An Lao, Binh Dinh Province, NMNS-8764-222–NMNS-8764-231 (Fig. 11G–I); 1D specimen, Binh Dinh Province, NMNS-8764-232; 7D + 14S specimens, Hoai An district, Binh Dinh Province, NMNS-8764-233–NMNS-8764-253.

##### Diagnosis.

Shell large and chirally dimorphic. Periostracum thick corneous with greenish brown radial streaks. Shell surface generally smooth. Genitalia with appendix.

##### Differential diagnosis.

*Amphidromusplacostylus* is similar to *A.schomburgki* (Pfeiffer, 1860) in having greenish to greenish brown radial streaks on periostracum, but *A.placostylus* has a larger shell (height up to nearly 80 mm) with a whitish apertural lip, and *A.schomburgki* exhibits a relatively smaller shell (height up to 58 mm) with a purplish apertural lip. *Amphidromusplacostylus* is also similar to *A.cambojiensis* (Reeve, 1860) in having a relatively large shell and ovate to elongate conical shape, but *A.placostylus* possesses a thick greenish periostracum, uniform whitish shell ground colour, and whitish to dark brown inner side of outer wall. In comparison, *A.cambojiensis* possesses a thin corneous periostracum, with irregular brown to dark brown radial streaks on the shell ground colour, and a bright purplish pink or violet colour on the inner side of outer wall. *Amphidromusplacostylus* is also recognised by a distinct clade in the molecular phylogeny (Fig. 2), with the closest *p*-distance to *A.bozhii* in COI (9.61%) and *A.ingens* in 16S (4.23%) (Table 2).

##### Description.

***Shell*** large (height 64.6–79.5 mm, width 37.4–42.4 mm), chirally dimorphic, solid, and ovate to elongate conical shape. Spire long conical with white colour; apex acute without black spot on tip. Whorls 6–7 convex; suture wide and depressed; last whorl ovate. Periostracum thick corneous or with oblique greenish to greenish brown radial streaks; varix usually absent. Shell surface generally smooth. Shell ground colour monochrome whitish or with dark brownish streaks (without periostracum). Parietal callus thickened and white. Aperture broadly ovate and inner side of outer wall with whitish to dark brown colour; peristome thickened, expanded, and reflexed but not attached to last whorl; lip whitish. Columella white, straight, or little twisted. Umbilicus imperforate.

***Radula*.** Teeth arranged in anteriorly pointed V-shaped rows. Central tooth monocuspid and trapezoid-spatulate with truncated cusp. Lateral teeth bicuspid; endocone small, with wide notch and truncated to slightly curved cusp; ectocone large with curved to dull cusp. Lateral teeth gradually transformed to asymmetric tricuspid marginal teeth (Fig. 12A).

***Genital organs*.** Atrium relatively short. Penis slender, conical, and short ~ 1/2 of vaginal length. Penial retractor muscle thin, long, inserting on epiphallus close to penis. Epiphallus long, slender tube. Flagellum long, extending from epiphallus and weakly coiled at its end. Appendix short, slender tube, 2× longer than flagellum, and approximately as long as epiphallus. Vas deferens slender tube passing from free oviduct and terminating at epiphallus-flagellum junction (Fig. 9D). Internal wall of penis corrugated, exhibiting series of weak longitudinal penial pilasters nearly entire inner penis wall. Penial verge short conical, nearly smooth surface and with opening on the tip (Fig. 9E).

Vagina slender, long cylindrical, and ~ 2× longer than penis. Gametolytic duct enlarged cylindrical tube then abruptly tapering to slender tube terminally and connected to gametolytic sac (missing during dissection). Free oviduct short; oviduct compact, enlarged to form lobule alveoli (Fig. 9D). Internal wall of vagina possessing corrugated smooth surface ridges on nearly its entire inner wall; ridges becoming thinner vaginal pilasters in middle, and with little irregular shaped and crenelations close to free oviduct opening (Fig. 9E).

***Living specimens*** with soft body morphology generally similar to *A.ingens*. Animals with dark reddish body covered with reticulated skin. Foot broad and long with uniform pale brown colour at foot margin. Head with reddish colour same as body. Upper and lower tentacles with reddish to orange in colour (Fig. 6D).

##### Distribution.

The distribution range of the species covers Bac Giang, Binh Dinh and Gia Lai provinces, Vietnam.

##### Remarks.

As the original description did not explicitly designate a type or state that the description of this species was based on a single specimen (nor could this be inferred), the designation of a holotype by Zilch (1953) in fact constitutes a lectotype designation (ICZN 1999: Art. 74.6).

This species is known only from a single worn-out lectotype, and the remaining periostracum is only traceable behind the apertural lip. Later, Thach and Huber in Thach (2017) introduced *A.johnstanisici*, which is described to differ from *A.placostylus* by the presence of prominent subsutural bands, larger aperture, more voluminous body whorl with dark brown colour, and parietal wall not bordered by a black band. However, both type materials of *A.johnstanisici* and *A.placostylus*, and all the specimens examined herein, especially ones from the type locality of *A.johnstanisici*, possess both subsutural bands and a black band that borders the parietal wall to some extent. These specimens and the holotype of *A.johnstanisici* also match well with the lectotype of *A.placostylus* in shell and apertural shape, and the periostracum colour. Thus, *A.johnstanisici* is regarded herein as a junior subjective synonym of *A.placostylus*. The periostracum colour can vary from greenish to greenish brown in the younger adult specimens (with thinner apertural lip), while the aged adult specimens (with thicker apertural lip) tend to have yellowish brown to eroded periostracum.

This species also exhibits a prominent population genetic structure, where specimens from the same collecting locality form its own clade (Fig. 13). The COI intraspecific distance among all *A.placostylus* specimens is 5.47%, which is the third highest distance of all *Amphidromus* species in this study. This value is higher than the optimum intra/interspecific threshold value of 4% for stylommatophoran land snails (Davison et al. 2009). In addition, the 16S intraspecific distance among all *A.placostylus* specimens is 3.14%, which is the second highest distance of all *Amphidromus* species in this study. Although each clade constitutes the specimens with the same inner shell colour (Fig. 13), all specimens still have other congruent shell morphology as stated above. We thus refrain from treating each pool of samples from the same collecting locality as a distinct taxon, before more specimens from each locality are critically examined.

#### ﻿﻿ Amphidromus ingensoides

Taxon classificationAnimaliaStylommatophoraCamaenidae

Jirapatrasilp & Lee sp. nov.

4332AAE8-EEE1-5D79-B5C3-173AFB4287D2

https://zoobank.org/BB594FC7-4E23-432C-AAA9-FE4C27BBB633

**Figure 14.**
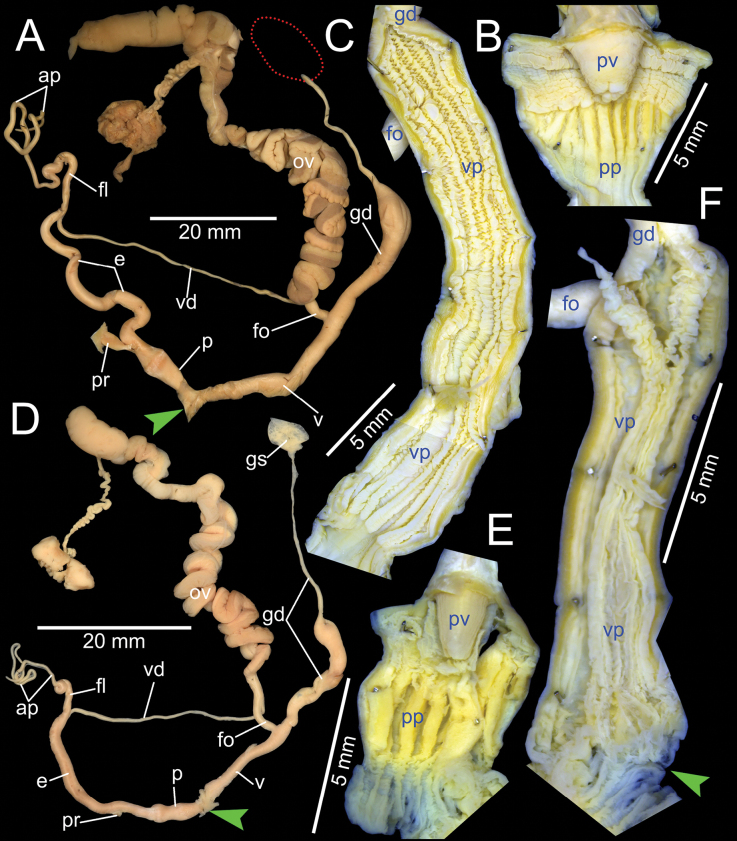
Genitalia of *Amphidromus* spp **A–C***Amphidromusingensoides* sp. nov. from Hon Ba, Khanh Son, Khanh Hoa, Vietnam (NMNS-8764-107), showing **A** general view of genitalia **B** interior structures of penis **C** interior structures of vagina chamber **D–F***Amphidromusbuelowi* Fruhstorfer, 1905 from Nha Trang, Khanh Hoa, Vietnam (NMNS-8764-031), showing **D** general view of genitalia **E** interior structures of penis **F** interior structures of vagina chamber. Red dots indicate the shape of the missing gametolytic sac. Green arrows indicate the genital openings. Abbreviations: ap, appendix; e, epiphallus; fl, ﬂagellum; fo, free oviduct; gd, gametolytic duct; gs, gametolytic sac; ov, oviduct; p, penis; pp, penial pilaster; pr, penial retractor muscle; pv, penial verge; v, vagina; vd, vas deferens; vp, vaginal pilaster

Figs 6E, F , 8G–J , 14A–C

##### Diagnosis.

Shell large and chirally dimorphic. Shell surface with coarse growth lines crossed by weak spiral ridges. Genitalia with appendix.

##### Differential diagnosis.

The new species differs from the closely related *A.ingens* and *A.bozhii* in having a generally rounded last whorl, and coarse growth lines crossed with weak spiral ridges. In comparison, the two latter species having a depression area below suture and prominent blunt or keeled on periphery of the last whorl, and having only irregular growth lines on the shell surface. In addition, this new species is recognised by a distinct clade in the molecular phylogeny (Fig. 2), with the closest *p*-distance to *A.bozhii* in COI (9.99%) and *A.ingens* in 16S (4.19%) (Table 2).

##### Etymology.

The specific epithet *ingensoides* is from *ingens*, and the suffix –*oideus*, meaning ‘like or resembling’. This name refers to the resemblance in shell morphology of the new species to the nominal species *A.ingens*.

##### Type material.

***Holotype*.** Vietnam: dextral, shell height 62.1 mm, shell width 36.9 mm, with 6½ whorls, 13 Dec. 2016, coll. A. N. Pham (NMNS-8764-105, Fig. 8G). ***Paratypes*.** Vietnam: 1S specimen (NHMUK 20230614, Fig. 8H) from the type locality, 19 Sep. 2016, coll. A. N. Pham; 1D + 1S specimens, Hon Ba, Khanh Son District, Khanh Hoa Province, NMNS-8764-106, NMNS-8764-107, 31 Mar. 2017, coll. A. N. Pham (Fig. 8I, J).

##### Type locality.

Vietnam: Cu’Mta ward, Mdrak District, Dak Lak Province, 12°42'22.9"N, 108°45'13.9"E.

##### Description.

***Shell*** large (height 54.3–67.0 mm, width 32.8–36.8 mm), chirally dimorphic, solid, and ovate conical shape. Spire long conical to elongate conical, apex acute without black spot on tip. Whorls 5–7 convex; suture wide and depressed; last whorl well rounded to slightly angulated. Periostracum brownish to thin corneous; varix usually absent. Shell surface generally with coarse and irregular growth lines crossed by weak spiral ridges. Shell colour variable: monochrome (whitish, yellowish, tinted pink) to stained with dark brown to blackish below periphery. Parietal callus thickened and white, dilated at umbilical area. Aperture broadly ovate; inner side of outer wall with yellow or dark brown to blackish colour. Peristome thickened, expanded and reflexed but not attached to last whorl, lip whitish. Columella white, straight, or little twisted. Umbilicus imperforate.

***Genital organs*.** Atrium relatively short. Penis slender, conical, and short ~ 1/3 of vaginal length. Penial retractor muscle thickened, short and inserting on epiphallus close to penis. Epiphallus long, slender tube, coiled and twisted upon itself. Flagellum long, extending from epiphallus and terminating in slightly enlarged folded coil. Appendix short, slender tube, ~ 2× longer than flagellum, and approximately as long as epiphallus. Vas deferens slender tube passing from free oviduct and terminating at epiphallus-flagellum junction (Fig. 14A). Internal wall of penis corrugated, exhibiting series of prominent and swollen longitudinal penial pilasters forming fringe around penial wall, and with strong roughly surface around base of penial verge. Penial verge short conical with weak roughly surface, and with opening at the tip (Fig. 14B).

Vagina slender, long cylindrical, and ~ 3× longer than penis. Gametolytic duct enlarged cylindrical tube then abruptly tapering to slender tube terminally and connected to gametolytic sac (missing during dissection). Free oviduct short; oviduct compact and enlarged to form lobule alveoli (Fig. 14A). Internal wall of vagina possessing corrugated and deep crenelated ridges on nearly its entire vagina wall; ridges slightly smooth surface near genital orifice then becoming prominent vaginal pilasters in middle and close to free oviduct opening (Fig. 14C).

***Living specimens*** with soft body morphology generally similar to *A.ingens*. Animals with pale yellowish body covered with reticulated skin, anterior body usually with dark reticulated strip dorsally. Foot broad and long, and with narrow and orange colour stripe above foot margin. Head with orange patch covering tentacles. Upper and lower tentacles orange to paler in colour (Fig. 6E, F).

##### Distribution.

This species is found in Dak Lak and Khanh Hoa provinces, Vietnam.

##### Remarks.

As a small number of specimens were dissected, this new species seems to have a vagina shorter than penis + epiphallus length, while *A.ingens* and *A.bozhii* have a vagina almost as long as penis + epiphallus. In addition, *A.ingensoides* sp. nov. possesses a longer appendix than the geographically closer species *A.ingens* from M’drak District, Dak Lak Province.

#### ﻿﻿ Amphidromus buelowi

Taxon classificationAnimaliaStylommatophoraCamaenidae

Fruhstorfer, 1905

6083D4A5-3159-5FA7-9F2C-8B8BD37A108B

**Figure 15.**
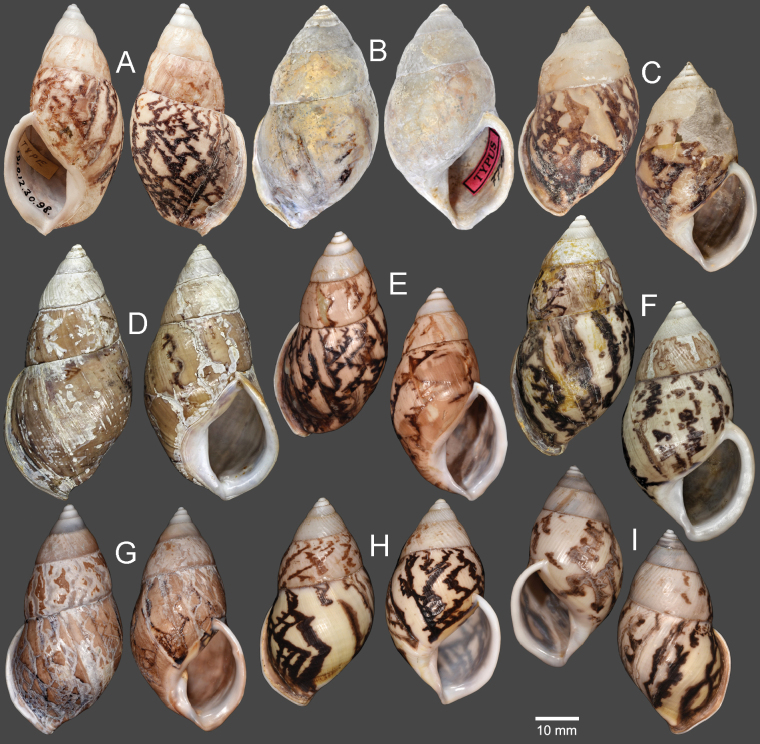
Shells of *Amphidromusbuelowi* Fruhstorfer, 1905 **A** lectotype of “*Amphidromusbuelowi*” (NHMUK 1910.12.30.98) **B** holotype of “*Amphidromusasper*” (SMF 7762) **C** holotype of “*Amphidromusfranzhuberi*” (MNHN-IM-2000-31892) **D** specimen from Padang Sökeli, Singalang, Indonesia (RBINS I.G. 10591) **E** specimen from Mount Singgalang, Sepuluh Koto, Tanah Datar Regency, West Sumatra, Indonesia (NMNS-8764-025) **F** specimen from Lang-Biang, Annam, Vietnam (RBINS I.G. 10591) **G** specimen from Lang-Biang plateau, Lac Duong, Lam Dong, Vietnam (NMNS-8764-027) **H, I** specimen from Nha Trang, Khanh Hoa, Vietnam (NMNS-8764-030, NMNS-8764-031). Credit: H. Taylor, NHM (**A**), M. Caballer, MNHN (**C**), RBINS (**D, F**).

**Figure 16.**
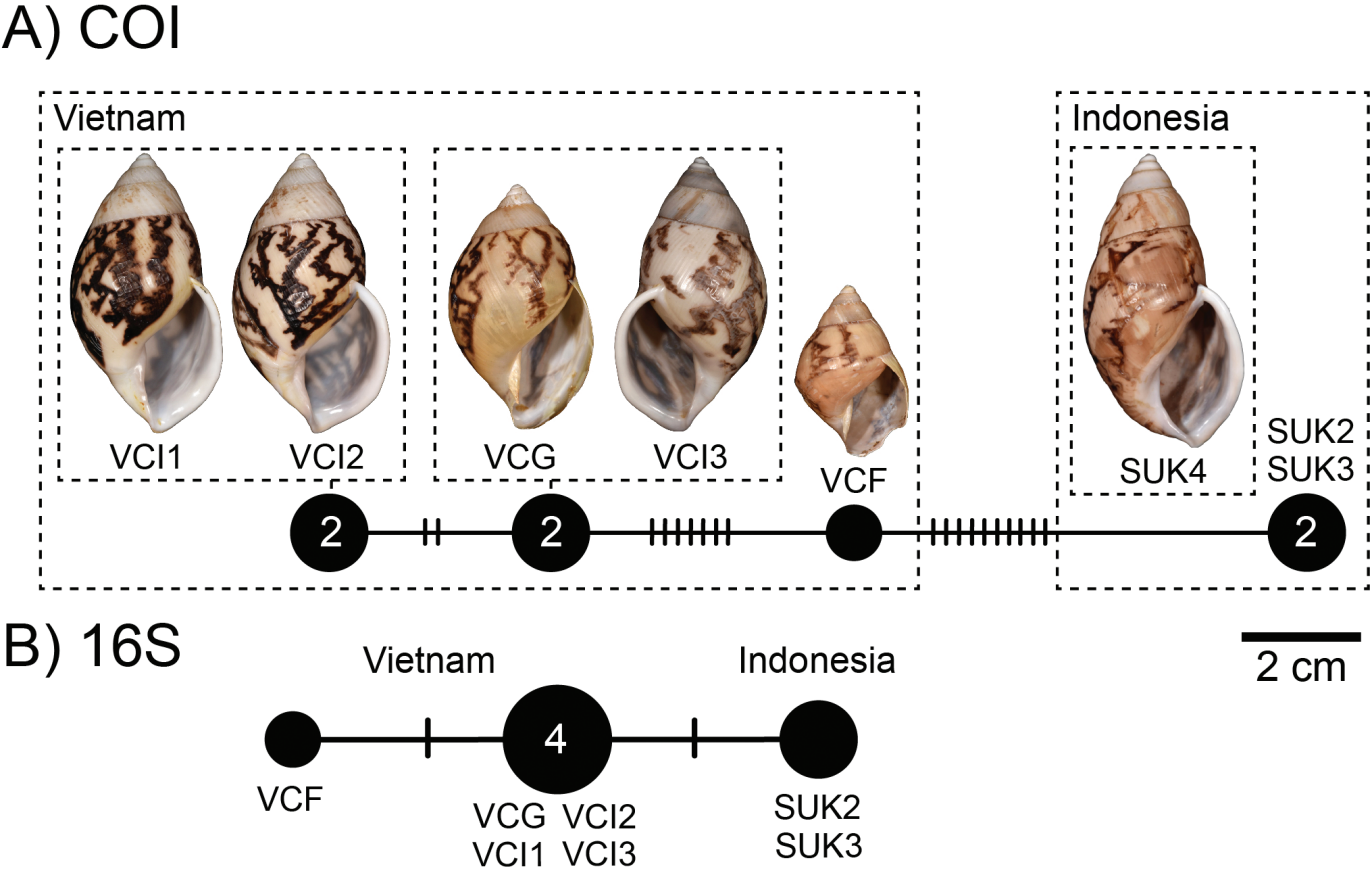
Mitochondrial haplotype minimum spanning networks of *Amphidromusbuelowi* Fruhstorfer, 1905 **A** COI and **B** 16S rRNA. The size of each circle corresponds to the frequency of that haplotype, also shown as the number in that circle. The bars on the branches indicate the number of mutational steps between haplotypes. Specimen codes correspond to those in Table 1.

Figs 6H, I , 12B , 14D–F , 15 , 16

Amphidromus (Goniodromus) bülowi Fruhstorfer, 1905: 83–84, pl. 1, fig. 2. Type locality: West-Sumatra. Rolle 1908: 67. Laidlaw and Solem 1961: 587, 606, fig. 37.
Amphidromusbülowi. Dautzenberg and Fischer 1906: 365–366, pl. 8, figs 10–12. Degner 1928: 360. Benthem Jutting 1959: 165.
Amphidromus (Goniodromus) asper Haas, 1934: 96, figs 11, 12. Type locality: Süd-Annam, 120 km von der Küste, auf dem Wege zum Plateau von Lang-Bian, zw. 600–1000 m [South Annam, 120 km from the coast, on the way to the plateau of Lang-Bian, between 600–1000 m]. Laidlaw and Solem 1961: 588, 601. Zilch 1953: 138, pl. 25, fig. 44. syn. nov.
Amphidromus asper . Schileyko 2011: 49. Páll-Gergely et al. 2020: 49, 51, fig. 15. Thach 2020a: pl. 76, fig. 893 right.
Amphidromus bulowi [sic]. Huber 2015: figs 9, 10. Sutcharit et al. 2015: 61, fig. 4e.
Amphidromus (Goniodromus) bulowi bulowi [sic]. Parsons and Abbas 2016: 240–242, figs 4 bottom, 5, 6a, b, d, 7.
Amphidromus franzhuberi Thach, 2016: 64–65, fig. 42; pl. 23, figs 315–319. Type locality: along the border of Nha Trang outskirts and Khanh Vinh District, Khanh Hoa Province (Central Vietnam). Páll-Gergely et al. 2020: 50, fig. 14. Thach 2020a: 58, pl. 76, fig. 893 left. Thach 2021: 60 syn. nov.
Amphidromus buelowi . Páll-Gergely et al. 2020: fig. 16.

##### Material examined.

Indonesia: Sinistral, ***lectotype*** of “*Amphidromusbuelowi*”, NHMUK 1910.12.30.98 (Fig. 15A). Vietnam: Dextral, ***holotype*** of “*Amphidromusasper*”, SMF 7762 (Fig. 15B). Dextral, ***holotype*** of “*Amphidromusfranzhuberi*”, MNHN-IM-2000-31892 (Fig. 15C).

##### Other material examined.

Indonesia: 2D specimens, Padang Sökeli, Singalang, RBINS I.G. 10591/1–2 (Fig. 15D); 4D specimens, Mount Singgalang, Sepuluh Koto, Tanah Datar Regency, West Sumatra, NMNS-8764-022–NMNS-8764-025 (Fig. 15E).

Vietnam: 2D specimens, Lang-Biang, Annam, RBINS I.G. 10591/3–4 (Fig. 15F); 2D specimens, Lang-Biang plateau, Lac Duong District, Lam Dong Province, NMNS-8764-026, NMNS-8764-027 (Fig. 15G); 6D + 1S specimens, Nha Trang, Khanh Hoa Province, NMNS-8764-028–NMNS-8764-034 (Fig. 15H, I).

##### Diagnosis.

Shell large and chirally dimorphic. Shell colour with irregularly zigzag of dark radial streaks, and dark triangular blotches. Aperture elliptical ovate with more or less prominent anterior notch and umbilical hump; twisted columella plait. Genitalia with appendix.

##### Differential diagnosis.

*Amphidromusbuelowi* differs from the similar species *A.asperoides* sp. nov. in having a distinct twisted columella plait, a prominent umbilical hump encircling columellar area, and an apertural notch projecting anteriorly. In contrast, *A.asperoides* sp. nov. possesses a straight columella, and without apertural notch and umbilical hump. In addition, on the soft body of living snail, the entire body of *A.buelowi* is reddish orange, while *A.asperoides* sp. nov. exhibits a uniform brownish yellow to pale brown body. *Amphidromusbuelowi* is also recognised by a distinct clade in the molecular phylogeny (Fig. 2), with the closest *p*-distance to *A.ingens* in COI (12.23%) and *A.asperoides* sp. nov. and *A.ingens* in 16S (4.61%) (Table 2).

##### Description.

***Shell*** large (height 45.3–51.1 mm, width 26.2–26.6 mm), chirally dimorphic, solid, and ovate conical. Spire conical with white or pale colour; apex acute without black spot on tip. Whorls 6–7 little convex to smooth; suture wide and shallow; last whorl well rounded to slightly elongated and with more or less prominent umbilical hump. Periostracum thin corneous; varices generally present. Shell ground colour pale yellowish, decorated with irregular zigzag of dark radial streaks, and dark triangular blotches connected with dark streaks. Parietal callus thickened, white and much thinner in central area. Aperture elliptical ovate; with more or less anterior notch; inner side of outer wall whitish colour; peristome thickened, expanded, and reflexed but not attached to last whorl; lip whitish. Columella white, straight and with distinct twisted plait. Umbilicus imperforate.

***Radula*.** Teeth arranged in anteriorly pointed V-shaped rows. Central tooth monocuspid and slightly elongate-spatulate teeth with truncated cusp. Lateral teeth bicuspid; endocone curved with wide notch and blunt cusp; ectocone large with truncated cusp. Lateral teeth gradually transformed to asymmetric tricuspid marginal teeth. Outermost teeth with small and curved cusp on ectocone, and endocone and mesocone with curved cusps (Fig. 12B).

***Genital organs*.** Atrium relatively short. Penis slender, conical, and nearly as long as vagina. Penial retractor muscle inserting on epiphallus close to penis. Epiphallus long, slender tube, and almost same diameter as penis. Flagellum short, extending from epiphallus and terminating in slightly enlarged folded coil. Appendix short, thin tube, 3× longer than flagellum, and approximately as long as epiphallus. Vas deferens slender tube passing from free oviduct and terminating at epiphallus-flagellum junction (Fig. 14D). Internal wall of penis corrugated, exhibiting series of thickened and swollen longitudinal penial pilasters forming fringe around penial wall, and with weaker folds around base of penial verge. Penial verge short conical with nearly smooth surface (Fig. 14E).

Vagina slender, long cylindrical, and ~ 2× longer than penis. Gametolytic duct enlarged cylindrical tube then abruptly tapering to long, slender tube terminally, connected to elongate gametolytic sac. Free oviduct short; oviduct compact, enlarged to form lobule alveoli (Fig. 14D). Internal wall of vagina possessing corrugated ridges near genital orifice; ridges becoming thinner and smooth longitudinal vaginal pilasters in middle, swollen with irregularly shaped deep crenelations close to free oviduct opening (Fig. 14F).

***Living specimens*** with soft body morphology generally similar to *A.ingens*. Animals with reddish orange body covered with reticulated skin. Lateral of body vary from yellowish (in younger specimen) to dark reddish orange colour (older specimens). Foot broad and long with reddish orange colour near foot sole margin. Head and dorsal of anterior body with reddish orange to dark colour. Upper tentacles pale reddish orange to brownish; lower tentacles short and paler in colour (Fig. 6H, I).

##### Haplotype network.

There was a total of four COI haplotypes (Fig. 16A) and three 16S haplotypes (Fig. 16B) of *A.buelowi* in this study, and the highest numbers of mutational steps in the COI and 16S minimum spanning networks are ten and one, respectively.

##### Distribution.

The species has a widely disjunct distribution: one in Mount Singgalang, West Sumatra, Indonesia, and some localities in Khanh Hoa and Lam Dong provinces, South Vietnam.

##### Remarks.

This species was originally described by Fruhstorfer (1905) from four chirally dimorphic specimens from West Sumatra. Fruhstorfer (1905) also indicated that there was a similar species collected on the way to the Lang-Bian plateau, ~ 120 km inland from the coast in southern Vietnam. He sent one specimen from this locality to O.F. von Möllendorff, who did not describe or taxonomically treat this specimen any further. Later, Haas (1934), recognising that there were some differences in shell characters to *A.buelowi*, described this particular specimen (now deposited in SMF) as a new species, *A.asper*. Thach (2016) also described a similar species, *A.franzhuberi* from the border of Nha Trang, Vietnam, which is described to differ from *A.buelowi* in having a broader shell shape, more swollen body whorls, a less excavated base, a more inflated spire, a rounded anterior end of the outer lip, and monomorphic dextrality (just from four type series). However, Thach (2016) did not compare with *A.asper* from the nearby area. In this study, the samples from Nha Trang exhibit dimorphic chirality (the specimen lot containing both sinistral and dextral shell coiling; Fig. 15H, I), and upon examining the type specimens of *A.asper* and *A.franzhuberi*, they agree well with the type specimen of *A.buelowi* in having the common diagnostic traits of a distinct twisted columella plait, a prominent umbilical hump, and a distinct apertural notch. The molecular phylogeny also revealed that all specimens from Mount Singgalang, West Sumatra, Indonesia, and Lang-Biang plateau and Nha Trang, Vietnam belong to the same clade. The mutational steps between Indonesian and Vietnamese specimens are only ten and one in the COI and 16S haplotype networks, respectively (Fig. 16). Based on the phylogenetic analyses and the common morphological diagnostics, we therefore treat *A.asper* and *A.franzhuberi* as junior subjective synonyms of *A.buelowi*.

**Figure 17.**
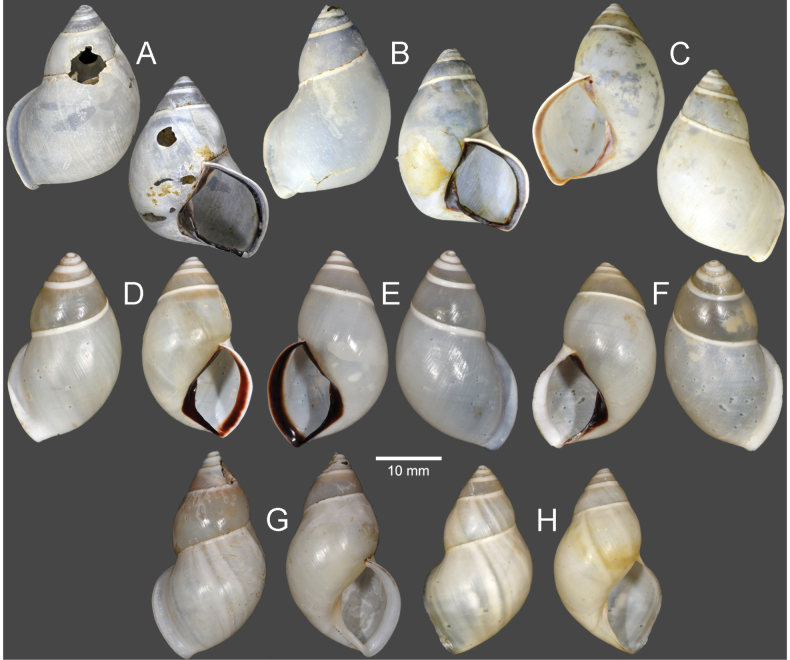
Shells of *Amphidromusthachi* Huber, 2015 **A** holotype of “*Amphidromusthachi*” (RBINS MT.3381) **B, C** specimens from fin de la route de Hon Ba (chalets de Yersin), Commune de Suoi Cat, Province de Khanh Hoa, Vietnam (MNHN- IM-214-6873) **D** specimen from Vinh Thanh, Binh Dinh, Vietnam (NMNS-8764-267) **E** Specimen from Buon Don, Dak Lak, Vietnam (NMNS-8764-271) **F** specimen from Da Lat, Lam Dong, Vietnam (NMNS-8764-272) **G, H** specimens from Lac Duong, Lam Dong, Vietnam (NMNS-8764-265, NMNS-8764-264). Credit: T. Backeljau and S. Yves, RBINS (**A**), B. Páll-Gergely (**B, C**).

Bülow (1905) introduced the monotypic subgenus Goniodromus to include *A.buelowi*, based on a less ovate aperture with an apertural notch projecting anteriorly. Later, Laidlaw and Solem (1961), although with doubt, listed *Goniodromus* as one of the three subgenera of *Amphidromus*, and included two more species, *A.asper* and *A.mirandus* Bavay & Dautzenberg, 1912. Another species, *A.thachi*, also possesses an aperture with prominent anterior notch (Fig. 17). However, these three species, *A.buelowi* (and its synonyms *A.asper* and *A.franzhuberi*), *A.thachi*, and *A.mirandus* did not together form a clade (Fig. 2; C-TL, unpublished data), revealing that an apertural anterior notch is not a shared derived character. Thus, the subgenus Goniodromus is regarded herein as a junior subjective synonym of the subgenus Amphidromus.

#### ﻿﻿ Amphidromus thachi

Taxon classificationAnimaliaStylommatophoraCamaenidae

Huber, 2015

4B2B8570-8EE9-53DD-A458-4E3FA857FD70

**Figure 18.**
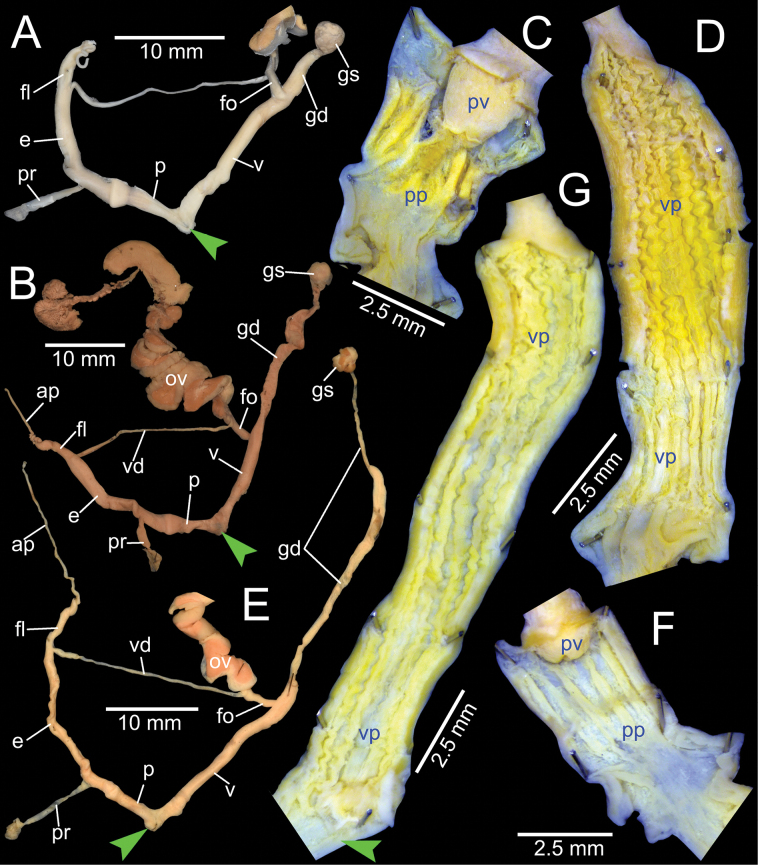
Genitalia of *Amphidromus* spp **A–D***Amphidromusthachi* Huber, 2015 **A** general view of genitalia of specimen from Krong Bong, Dak Lak, Vietnam (NMNS-8764-274) **B–D** specimen from Buon Don, Dak Lak, Vietnam (NMNS-8764-271), showing **B** general view of genitalia **C** interior structures of penis **D** interior structures of vagina chamber **E–G***Amphidromusmetabletus* Möllendorff, 1900 from Nha Trang, Khanh Hoa, Vietnam (NMNS-8764-130), showing **E** general view of genitalia **F** interior structures of penis **G** interior structures of vagina chamber. Green arrows indicate the genital openings. Abbreviations: ap, appendix; e, epiphallus; fl, ﬂagellum; fo, free oviduct; gd, gametolytic duct; gs, gametolytic sac; ov, oviduct; p, penis; pp, penial pilaster; pr, penial retractor muscle; pv, penial verge; v, vagina; vd, vas deferens; vp, vaginal pilaster.

**Figure 19.**
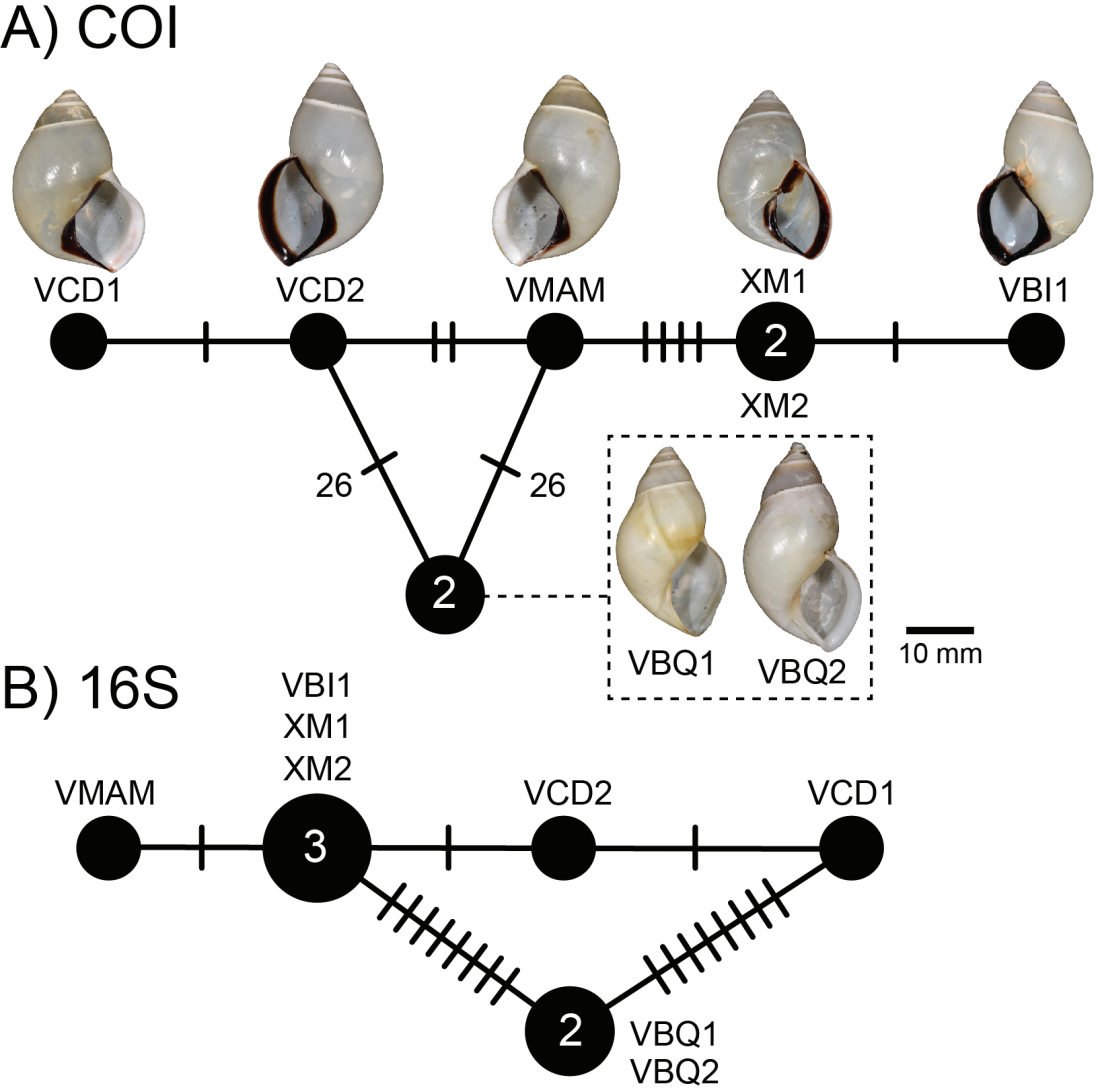
Mitochondrial haplotype minimum spanning networks of *Amphidromusthachi* Huber, 2015 **A** COI and **B** 16S rRNA. The size of each circle corresponds to the frequency of that haplotype, also shown as the number in that circle. The bars on the branches indicate the number of mutational steps between haplotypes. Specimen codes correspond to those in Table 1.

Figs 6J , 12C , 17 , 18A–D , 19

Amphidromus thachi Huber, 2015: 29–30, figs 1–8. Type locality: outskirts of Nha Trang area, about 30 km southeast of Nha Trang city (Cam Lam District, Khanh Hoa Province, central Vietnam), at some distance from the village and the National Road No 1A. Thach 2017: 47–48, pl. 53, fig. 668. Thach 2018: pl. 70, figs 838, 839. Thach 2021: 79.
Amphidromus thachi krisi Thach, 2018: 63–64, pl. 70, figs 833–837. Type locality: Lac Duong District, Lam Dong Province, South Vietnam. Thach 2021: 79.

##### Material examined.

Vietnam: Dextral, ***holotype*** of “*Amphidromusthachi*”, RBINS MT.3381 (Fig. 17A).

##### Other material examined.

Vietnam: 1D + 1S specimens, fin de la route de Hon Ba (chalets de Yersin), Commune de Suoi Cat, Province de Khanh Hoa, Vietnam, MNHN- IM-214-6873 (Fig. 17B, C); 1D specimen, réserve de Hon Ba, près du chalet de Yersin, Commune de Suoi Cat, Province de Khanh Hoa, Vietnam, MNHN- IM-214-6874; 3D + 1S specimens, Vinh Thanh town, Binh Dinh Province, NMNS-8764-266–NMNS-8764-269 (Fig. 17D); 1D + 1S specimens, Buon Don District, Dak Lak Province, NMNS-8764-270, NMNS-8764-271 (Fig. 17E); 1S specimen, Da Lat city, Lam Dong Province, NMNS-8764-272 (Fig. 17F); 2D specimens, Krong Bong, Dak Lak Province, NMNS-8764-273, NMNS-8764-274; 2D specimens, Lac Duong District, Lam Dong Province, NMNS-8764-264, NMNS-8764-265 (Fig. 17G, H).

##### Diagnosis.

Shell medium and chirally dimorphic. Aperture obliquely elliptical with prominent anterior notch; columella bending anteriorly. Parietal callus, lip and columella whitish or with dark brown. Genitalia with appendix.

##### Differential diagnosis.

*Amphidromusthachi* is unique compared to all Vietnamese species reported by Schileyko (2011) in having a distinct shell shape, possessing an obliquely elliptical aperture with a prominent anterior notch, a columella bending anteriorly, and whitish or dark brown parietal callus, lip and columella. This type of shell form is similar to that of *Pseudopartula* Pfeiffer, 1856 (Benthem Jutting 1950). *Amphidromusthachi* is also recognised by a distinct clade in the molecular phylogeny (Fig. 2), with the closest *p*-distance to *A.asperoides* sp. nov. in both COI (12.69%) and 16S (6.22%) (Table 2).

##### Description.

***Shell*** medium (height 25.0–30.0 mm, width 17.0–18.5 mm), chirally dimorphic, thin to slightly thickened, and conical. Spire short conical with white or pale colouration; apex acute without black spot on tip. Whorls 6–7 little convex to smooth; suture wide and shallow; last whorl well rounded to slightly elongated and with less prominent umbilical hump. Periostracum thin corneous; varices absent. Shell colour uniform whitish to pale cream; subsutural band opaque white. Parietal callus thickened, whitish and translucent or dark to dark brown. Aperture elliptical to obliquely elliptical with prominent anterior notch; inner side of outer wall whitish; peristome thickened, slightly expanded not reflected; lip whitish or with dark to dark brown. Columella whitish or dark, shortly straight then bending anteriorly. Umbilicus imperforate.

***Radula*.** Teeth arranged in anteriorly pointed V-shaped rows. Central tooth monocuspid and spatulate with truncated cusp. Lateral teeth bicuspid; endocone slightly smaller than ectocone, curved, with wide notch and dull cusp; ectocone large with curved to dull cusp. Lateral teeth gradually transformed to asymmetric tricuspid marginal teeth. Outermost teeth with small and curved cusp on ectocone; endocone and mesocone with curved cusps (Fig. 12C).

***Genital organs*.** Atrium relatively short. Penis slender, conical, and short, ~ 1/2 of vaginal length. Penial retractor muscle thickened and inserting on epiphallus close to penis. Epiphallus long, slender tube, almost same diameter as penis. Flagellum short, extending from epiphallus and terminating in weakly coiled. Appendix short, slender tube, similar length with flagellum, and ~ 1/2 of epiphallus length. Vas deferens slender tube passing from free oviduct and terminating at epiphallus-flagellum junction (Fig. 18A, B). Internal wall of penis corrugated, exhibiting series of thickened and smooth surfaced longitudinal penial pilasters forming fringe around penial wall, and with nearly smooth wall around base of penial verge. Penial verge short conical with smooth surface (Fig. 18C).

Vagina slender, cylindrical, and ~ 2× longer than penis. Gametolytic organ relatively short than other congeners: gametolytic duct shorter to slightly longer than vagina, cylindrical tube, then tapering to short, slender tube terminally; gametolytic sac globular shape. Free oviduct short; oviduct compact, enlarged to form lobule alveoli (Fig. 18A, B). Internal wall of vagina possessing smooth longitudinal ridges near genital orifice; ridges becoming stronger and corrugated vaginal pilasters with swollen, irregular shaped and deep crenelations (Fig. 18D).

***Living specimens*** with soft body morphology generally similar to *A.ingens*. Animals with whitish to creamy body covered with reticulated skin. Foot broad and long with uniform whitish to creamy colouration to posterior tail. Head with whitish or sometimes with yellowish colour. Upper tentacles drumstick-shaped, greyish to brownish, with dark eyespots on tentacular tips; lower tentacles short and greyish in colour (Fig. 6J).

##### Haplotype network.

There was a total of six COI haplotypes (Fig. 19A) and five 16S haplotypes (Fig. 19B) of *A.thachi* in this study, and the highest numbers of mutational steps in the COI and 16S minimum spanning networks are 26 and eight, respectively.

##### Distribution.

The distribution range of this species covers Binh Dinh, Dak Lak, Khanh Hoa, and Lam Dong provinces, Vietnam.

##### Remarks.

This species was originally described by Huber (2015) from outskirts of Nha Trang, Vietnam. Later, Thach (2018) described another subspecies from Lac Duong, Lam Dong, Vietnam as *A.thachikrisi*, which was different from the nominotypical subspecies in having a totally white lip. Based on this study, the specimens having a totally white lip from Lac Duong, Lam Dong constitutes a distinct clade from the remaining specimens with totally or partially dark lip, and the mutational steps between these two morphs with different lip colours are 26 and eight in the COI and 16S haplotype networks, respectively (Fig. 19). More specimens from wider distribution range will be needed to assess the taxonomic status of these *A.thachi* subspecies.

Two dissected specimens were found to have different lengths of the gametolytic duct. The specimen XM2 from Krong Bong, Dak Lak, Vietnam has a shorter gametolytic duct (Fig. 18A) than the specimen VCD2 from Buon Don, Dak Lak, Vietnam (Fig. 18B).

#### ﻿﻿ Amphidromus metabletus

Taxon classificationAnimaliaStylommatophoraCamaenidae

Möllendorff, 1900

AD2C18DB-FB27-5695-937E-87362BBF4FC2

**Figure 20.**
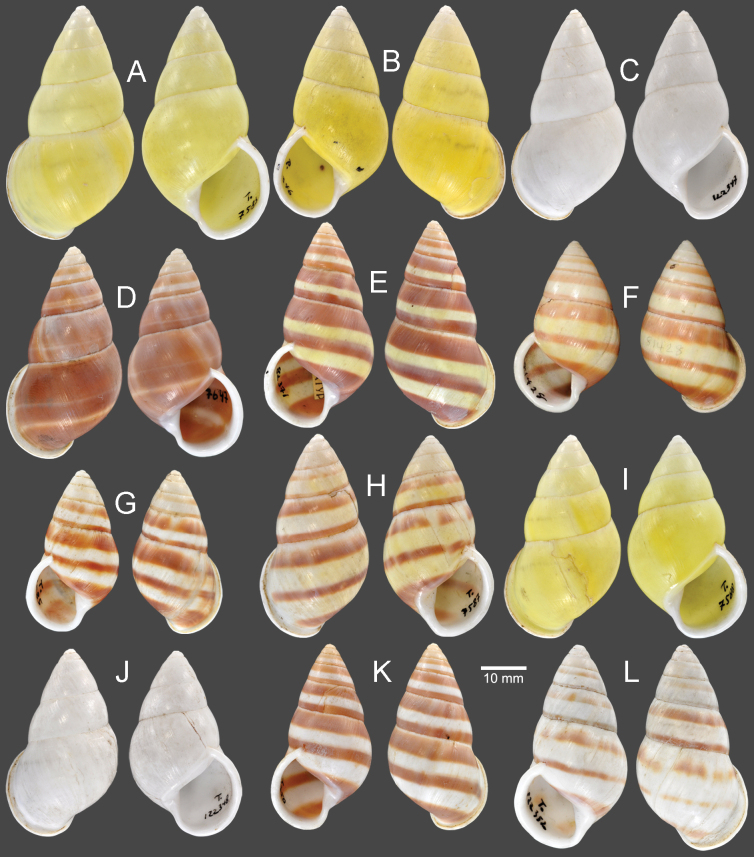
Shells of *Amphidromusmetabletus* Möllendorff, 1900 **A** lectotype of “*Amphidromusmetabletus*” (SMF 7583) **B–F** paralectotypes of “*Amphidromusmetabletus*” **B**SMF 122346 **C**SMF 122347 **D**SMF 7647 **E**SMF 82371 **F**ANSP 81428 **G** lectotype of “*Amphidromusmetabletusinsularis*” (SMF 7585) **H** lectotype of “Amphidromusmetabletuspachychilus ” forma tritaeniata (SMF 7587) **I** paralectotype of “Amphidromusmetabletuspachychilus ” forma flava (SMF 7588) **J** paralectotype of “Amphidromusmetabletuspachychilus ” forma alba (SMF 122348) **K** paralectotype of “Amphidromusmetabletuspachychilus ” forma trizona (SMF 122350) **L** paralectotype of “Amphidromusmetabletuspachychilus ” forma interrupta (SMF 122352). Credit: ANSP (**F**).

**Figure 21.**
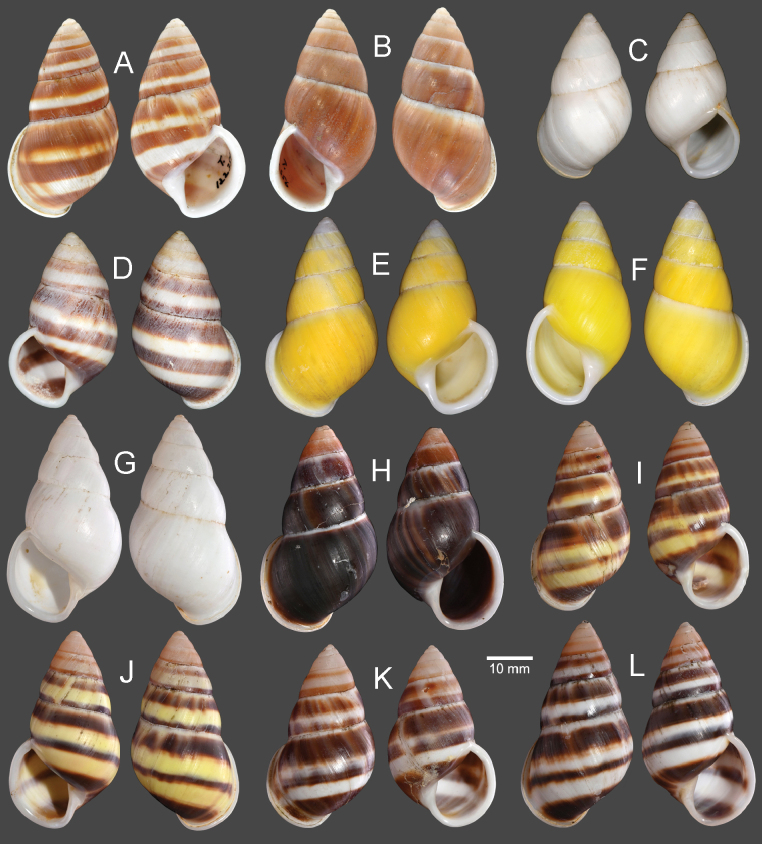
Shells of *Amphidromusmetabletus* Möllendorff, 1900 **A** paralectotype of “Amphidromusmetabletuspachychilus ” forma confluens (SMF 122354) **B** paralectotype of “Amphidromusmetabletuspachychilus ” forma fusca (SMF 122356) **C–F** specimens from Nha Trang, Khanh Hoa, Vietnam (NMNS-8764-123, NMNS-8764-125, NMNS-8764-127, NMNS-8764-129) **G–L** specimens from Ninh Hoa, Khanh Hoa, Vietnam (NMNS-8764-136, NMNS-8764-143, NMNS-8764-144, NMNS-8764-146, NMNS-8764-147, NMNS-8764-149).

**Figure 22.**
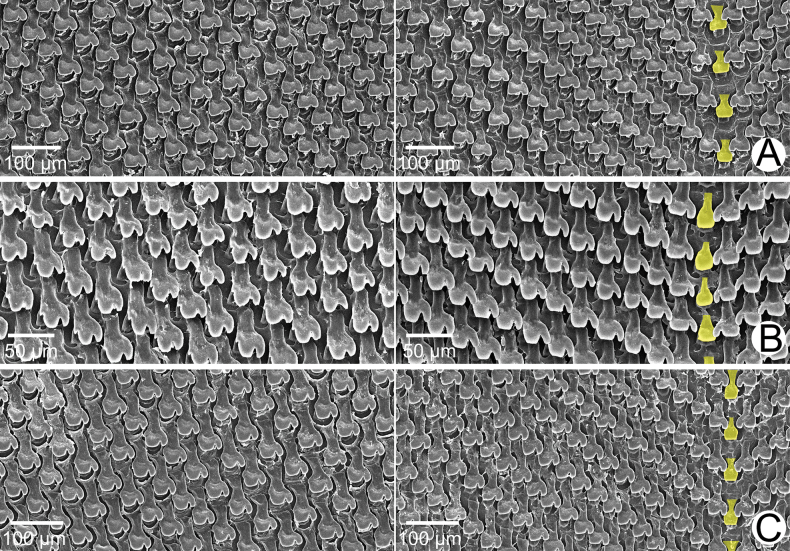
SEM images of the radula **A***Amphidromusmetabletus* Möllendorff, 1900 from Nha Trang, Khanh Hoa, Vietnam (NMNS-8764-130) **B***Amphidromusmadelineae* Thach, 2020 from Duy Xuyen, Quang Nam, Vietnam (NMNS-8764-110) **C***Amphidromuscostifer* Smith, 1893 from Ea Sup, Dak Lak, Vietnam (NMNS-8764-048). Central teeth are marked in yellow. The left and right images show the outer and inner sections of each radula, respectively.

**Figure 23.**
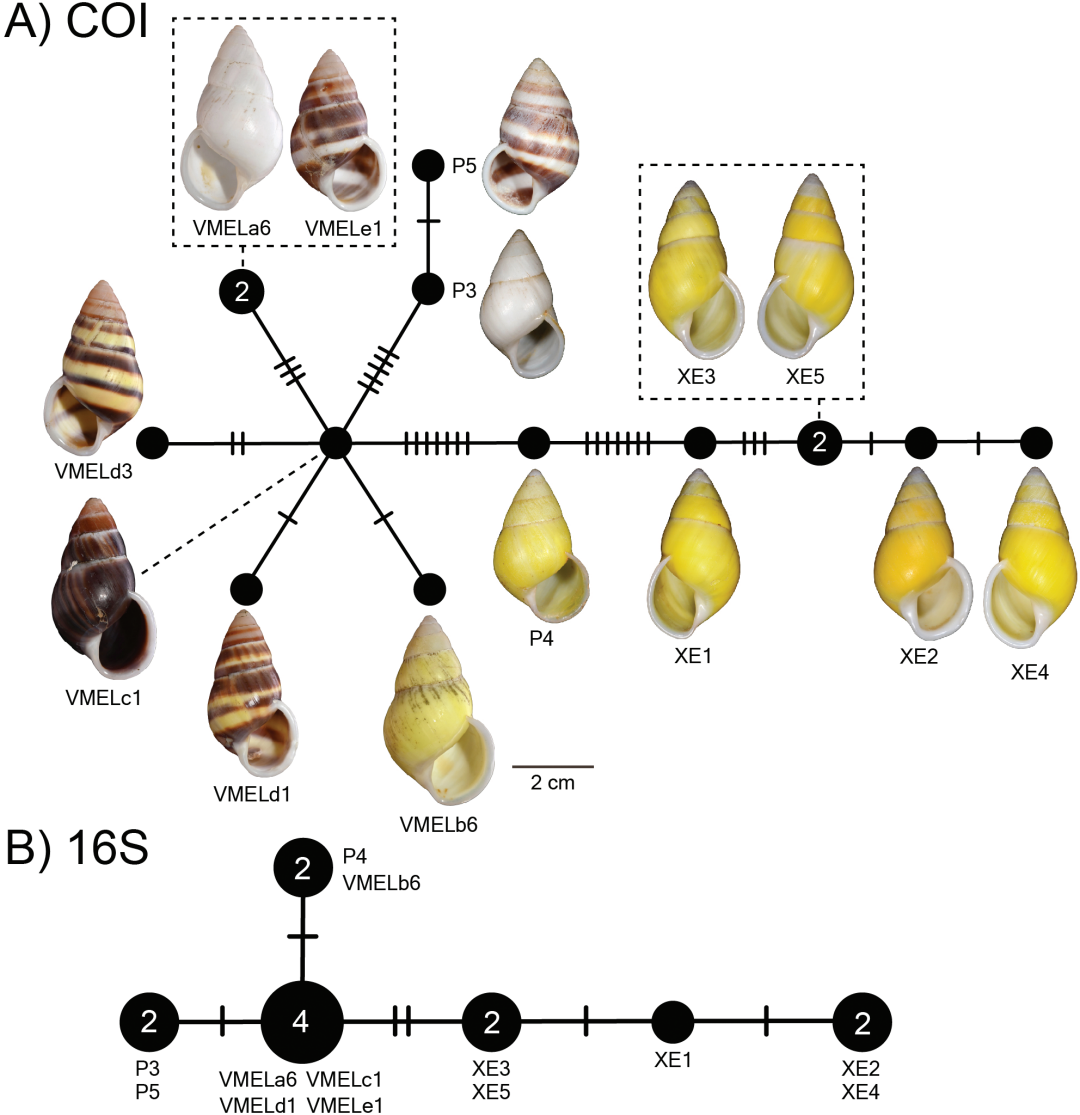
Mitochondrial haplotype minimum spanning networks of *Amphidromusmetabletus* Möllendorff, 1900 **A** COI and **B** 16S rRNA. The size of each circle corresponds to the frequency of that haplotype, also shown as the number in that circle. The bars on the branches indicate the number of mutational steps between haplotypes. Specimen codes correspond to those in Table 1.

Figs 18E–G , 20 , 21 , 22A , 23

Amphidromus metabletus Möllendorff, 1900b: 22–23. Type locality: Berg “Mutter und Kind”, Annam [Vietnam]. Pilsbry 1900: 174–175. Möllendorff 1901: 48–49. Pilsbry 1901: 168–169, pl. 49, figs 1–4. Laidlaw and Solem 1961: 528, 640. Solem 1966: 102. Richardson 1985: 29. Thach 2005: 236. Schileyko 2011: 50.
Amphidromus metabletus pachychilus Möllendorff, 1901: 49. Type locality: Nha-trang, Süd-Annam [Nha Trang, Khanh Hoa Province, Vietnam]. Laidlaw and Solem 1961: 649. Richardson 1985: 30. Thach 2005: 236, pl. 73, figs 8, 13, 14, 18–21. Schileyko 2011: 50.
Amphidromus metabletus insularis Möllendorff, 1901: 49–50. Type locality: Insel Bai-min bei Nha-trang. Laidlaw and Solem 1961: 629–630. Richardson 1985: 30. Schileyko 2011: 50.
Amphidromus metableta [sic]. Fischer and Dautzenberg 1904: 406.
Amphidromus metableta pachychilus [sic]. Fischer and Dautzenberg 1904: 406.
Amphidromus metableta insularis [sic]. Fischer and Dautzenberg 1904: 406.
Amphidromus (Amphidromus) metabletus metabletus. Zilch 1953: 137, pl. 24, fig. 30.
Amphidromus (Amphidromus) metabletusinsularis. Zilch 1953: 137, pl. 24, fig. 31.
Amphidromus (Amphidromus) metabletus pachychilus. Zilch 1953: 137, pl. 24, figs 32–36; pl. 25, figs 37, 38.

##### Material examined.

Vietnam: Dextral, ***lectotype*** of “*Amphidromusmetabletus*”, SMF 7583/1 (Fig. 20A); 1S paralectotype of “*Amphidromusmetabletus*”, SMF 122346/1 (Fig. 20B); 2D + 1S paralectotypes of “*Amphidromusmetabletus*”, SMF 122347/3 (Fig. 20C); 2D + 1S paralectotypes of “*Amphidromusmetabletus*”, SMF 7647/3 (Fig. 20D); 1D + 1S paralectotypes of “*Amphidromusmetabletus*”, SMF 82371/2 (Fig. 20E); 1S, paralectotype of “*Amphidromusmetabletus*”, ANSP 81428 (Fig. 20F). Sinistral, ***lectotype*** of “*Amphidromusmetabletusinsularis*”, SMF 7585/1 (Fig. 20G). Dextral, ***lectotype*** of “Amphidromusmetabletuspachychilus ” forma tritaeniata, SMF 7587/1 (Fig. 20H); 1D, paralectotype of “Amphidromusmetabletuspachychilus ” forma flava, SMF 7588/1 (Fig. 20I); 1D, paralectotype of “Amphidromusmetabletuspachychilus ” forma alba, SMF 122348/1 (Fig. 20J); 1S, paralectotype of “Amphidromusmetabletuspachychilus ” forma trizona, SMF 122350/1 (Fig. 20K); 1S, paralectotype of “Amphidromusmetabletuspachychilus ” forma interrupta, SMF 122352/1 (Fig. 20L); 1D, paralectotype of “Amphidromusmetabletuspachychilus ” forma confluens, SMF 122354/1 (Fig. 21A); 1S, paralectotype of “Amphidromusmetabletuspachychilus ” forma fusca, SMF 122356/1 (Fig. 21B).

##### Other material examined.

Vietnam: 4D + 4S specimens, Nha Trang city, Khanh Hoa Province, NMNS-8764-123–NMNS-8764-130 (Fig. 21C–F); 15D + 4S specimens, Ninh Hoa, Khanh Hoa Province, NMNS-8764-131–NMNS-8764-149 (Fig. 21G–L).

##### Diagnosis.

Shell medium to large, elongate conical, and chirally dimorphic. Spire elongate conical; aperture ovate. Genitalia with appendix.

##### Differential diagnosis.

The monochromic form of the chirally dimorphic *A.metabletus* is similar to *A.cochinchinensis* (Pfeiffer, 1857) in having a monochrome whitish yellow shell, but *A.cochinchinensis* is distinct in having a very little expanded lip, elongate last whorl, and elliptical aperture (Sutcharit et al. 2015). The banded form is similar to the chirally dimorphic *Aegistohadradautzenbergi* (Fulton, 1899), but *A.metabletus* has a shell ground colour varying from whitish, yellowish, to reddish brown, and the shell is without an umbilical hump, while *Ae.dautzenbergi* has a ground colour varying from whitish to yellowish and tinted pink, and the shell sometimes possesses an umbilical hump. *Aegistohadradautzenbergi* also has a thinner shell, a more ovate last whorl with an expanded lip that is not thickened or reflected, a thin parietal callus and a straight columella, whereas *A.metabletus* has a thicker shell, a rounder last whorl with an expanded and usually reflected lip, a thick parietal callus and a curved columella. Moreover, the genitalia of *A.metabletus* lack a dart complex, while it is present in all *Aegistohadra* species (Jirapatrasilp et al. 2022). *Amphidromusmetabletus* is also recognised by a distinct clade in the molecular phylogeny (Fig. 2), with the closest *p*-distance to *A.ingens* in COI (15.91%) and *A.thachi* in 16S (10.68%) (Table 2).

##### Description.

***Shell*** medium to large (height 36.5–46.5 mm, width 20.9–27.2 mm), chirally dimorphic, elongate conical, rather thick and glossy. Spire elongate conical to ovate conical; apex acute, without black spot on tip, and earlier whorls whitish to tinted pink. Whorls 6–7 convex to smooth; suture wide and shallow; last whorl well rounded. Periostracum thin corneous; varix usually absent. Shell ground colour varying from whitish, yellowish to reddish brown; banding pattern variable from non-banded (monochrome colour) to narrow to wide multiple reddish brown spiral bands on whitish or yellowish ground colour. Parietal callus slightly thickened, whitish or transparent. Aperture ovate; peristome expanded and not reflected; lip whitish. Columella straight, thick or thin. Umbilicus imperforate.

***Radula*.** Teeth arranged in anteriorly pointed V-shaped rows. Central tooth monocuspid and short spatulate with truncated cusp. Lateral teeth bicuspid; endocone small, with wide notch and blunt cusp; ectocone large with blunt cusp. Lateral teeth gradually transformed to asymmetric tricuspid marginal teeth (Fig. 22A).

***Genital organs*.** Atrium relatively short. Penis slender, and short ~ ¼ of vaginal length. Penial retractor muscle thin, long and inserting on epiphallus close to penis. Epiphallus long, slender tube, and almost same diameter as penis. Flagellum short, extending from epiphallus and terminating in slightly enlarged folded coil. Appendix long, slender tube, ~ 3× longer than flagellum, and approximately as long as epiphallus. Vas deferens slender tube passing from free oviduct and terminating at epiphallus-flagellum junction (Fig. 18E). Internal wall of penis corrugated, exhibiting series of swollen and smooth surfaced longitudinal penial pilasters forming fringe around entire penial wall. Penial verge very short conical with smooth surface (Fig. 18F).

Vagina slender, long cylindrical, and ~ 4× longer than penis. Gametolytic duct very long cylindrical tube then abruptly tapering to slender tube terminally and connected to globular gametolytic sac. Free oviduct short; oviduct compact, forming lobule alveoli (Fig. 18E). Internal wall of vagina possessing corrugated ridges with wide crenelations on its entire vagina wall; ridges becoming stronger corrugated close to free oviduct opening (Fig. 18G).

##### Haplotype network.

There were 12 COI haplotypes (Fig. 23A) and six 16S haplotypes (Fig. 23B) of *A.metabletus* in this study, and the highest numbers of mutational steps in the COI and 16S minimum spanning networks are seven and two, respectively.

##### Distribution.

This species is found in Khanh Hoa Province, Vietnam.

##### Remarks.

One species that was described earlier, *A.cochinchinensis* was originally described from “Cochin China”, the old geographic usage which is now interpreted as southern Vietnam, and this species was known only from the type materials (Sutcharit et al. 2015). This species is similar to *A.metabletus*, which is described from the same vicinity. The further inclusion of *A.cochinchinensis*-like specimens from southern Vietnam into the phylogenetic analyses will help clarify the taxonomic statuses of these two species.

Möllendorff (1901) introduced several subspecies and shell forms. However, these forms could not be differentiated by mtDNA (COI and 16S rRNA) and some shell morphs with different colours and patterns belong to the same mtDNA haplotype (Fig. 23).

#### ﻿﻿ Amphidromus haematostoma

Taxon classificationAnimaliaStylommatophoraCamaenidae

Möllendorff, 1898

3BD55284-A262-5A0F-AC51-C55398B2E715

**Figure 24.**
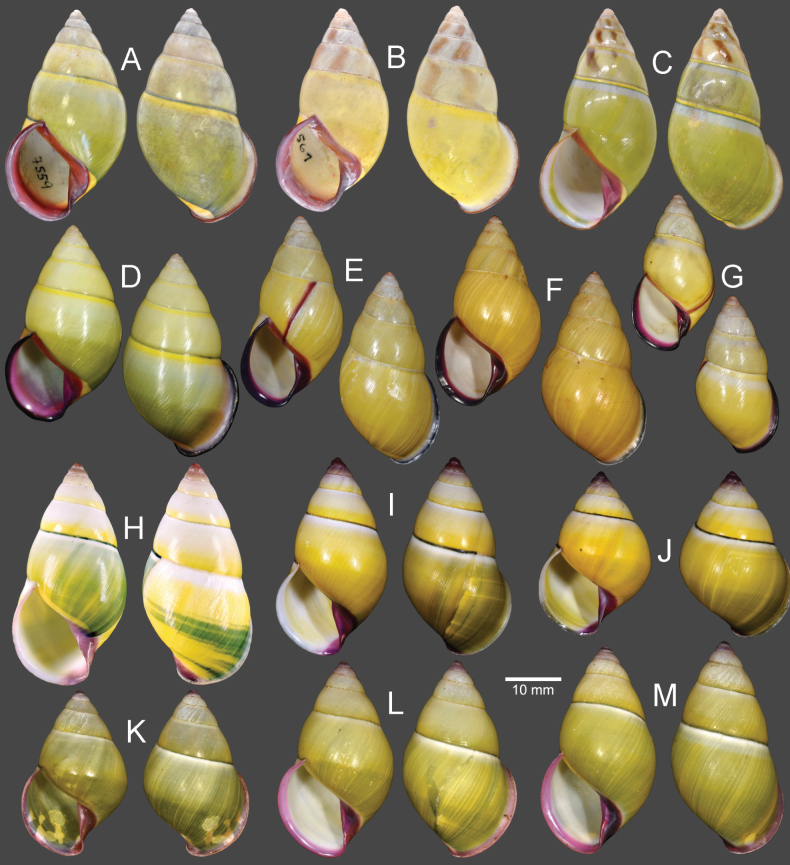
Shells of *Amphidromus* spp **A–G***Amphidromushaematostoma* Möllendorff, 1898 **A** Lectotype of “Amphidromushaematostomavar.viridis” (SMF 7559) **B** lectotype of “Amphidromushaematostomavar.varians” (SMF 7561) **C** holotype of “*Amphidromusattapeuensis*” (NHMUK 20170278) **D** specimen from Samphanh, Phongsali, Laos (NMNS-8764-056) **E, F** specimens from Ba Chien, Pakse, Champasak, Laos (NMNS-8764-064, NMNS-8764-076) **G** specimen from Kbang, Gia Lai, Vietnam (NMNS-8764-080) **H–M***Amphidromusmadelineae* Thach, 2020 **H** holotype of “*Amphidromusmadelineae*” (MNHN-IM-2000-35566) **I, J** Specimens from Duy Xuyen, Quang Nam, Vietnam (NMNS-8764-112, NMNS-8764-108) **K–M** specimens from Za Hung, Dong Giang, Quang Nam, Vietnam (NMNS-8764-114, NMNS-8764-118, NMNS-8764-122).

**Figure 25.**
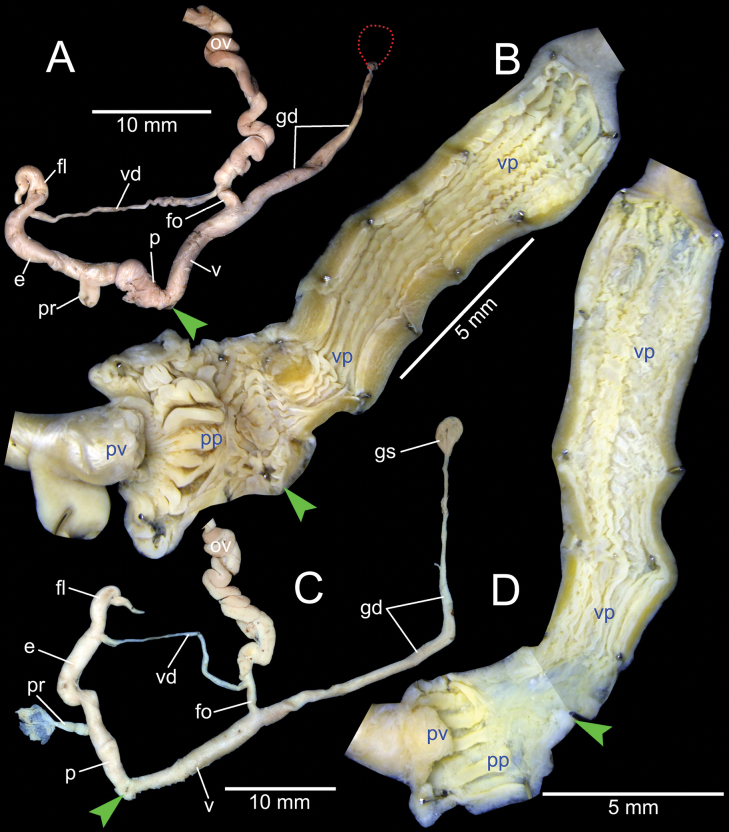
Genitalia of *Amphidromus* spp **A, B***Amphidromushaematostoma* Möllendorff, 1898 from Ba Chien, Pakse, Champasak, Laos (NMNS-8764-061), showing **A** general view of genitalia **B** interior structures of penis and vagina chamber **C, D***Amphidromusmadelineae* Thach, 2020 from Duy Xuyen, Quang Nam, Vietnam (NMNS-8764-110), showing **C** general view of genitalia **D** interior structures of penis and vagina chamber. Red dots indicate the shape of the missing gametolytic sac. Green arrows indicate the genital openings. Abbreviations: e, epiphallus; fl, ﬂagellum; fo, free oviduct; gd, gametolytic duct; gs, gametolytic sac; ov, oviduct; p, penis; pp, penial pilaster; pr, penial retractor muscle; pv, penial verge; v, vagina; vd, vas deferens; vp, vaginal pilaster.

**Figure 26.**
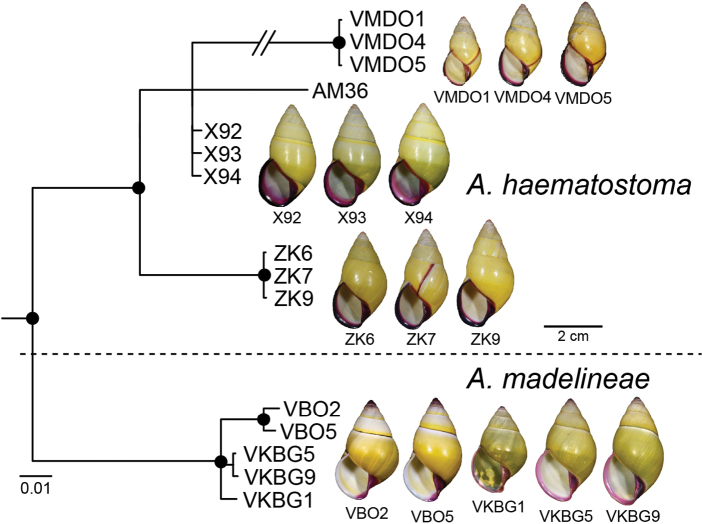
Bayesian phylogeny of *Amphidromushaematostoma* Möllendorff, 1898 and *Amphidromusmadelineae* Thach, 2020 based on mitochondrial COI and 16S genes. Nodal support values are given as SH-aLRT/aBayes/ultra-fast bootstrap (IQ-TREE, ML)/posterior probability (MrBayes, BI). An asterisk on each branch indicates a clade with all well-supported values (SH-aLRT ≥ 80%, aBayes ≥ 0.95, BS ≥ 95%, PP ≥ 0.95).

Figs 24A–G , 25A, B , 26

Amphidromus haematostoma Möllendorff, 1898: 74–75. Type locality: Boloven [Boloven Plateau, Champasak, Laos]. Pilsbry 1900: 182–183. Möllendorff 1901: 50. Pilsbry 1901: 169. Fischer and Dautzenberg 1904: 406. Richardson 1985: 19. Schileyko 2011: 51. Inkhavilay et al. 2019: 91, figs 43f, 44a–c.
Amphidromus haematostoma var. viridis Möllendorff, 1898: 75. Type locality: Boloven. Pilsbry 1900: 183. Fischer and Dautzenberg 1904: 406.
Amphidromus haematostoma var. varians Möllendorff, 1898: 75. Type locality: Boloven. Pilsbry 1900: 183. Fischer and Dautzenberg 1904: 406.
Amphidromus (Syndromus) haematostoma. Zilch 1953: 132, pl. 22, figs 4, 5. Inkhavilay et al. 2017: 34–35, figs 13o–r.
Amphidromus haematostomus [sic]. Laidlaw and Solem 1961: 527, 625.
Amphidromus haematostomus [sic] varians. Laidlaw and Solem 1961: 668.
Amphidromus haematostomus [sic] viridis. Laidlaw and Solem 1961: 670.
Amphidromus haematostoma varians . Richardson 1985: 19.
Amphidromus haematostoma viridis . Richardson 1985: 19.
Amphidromus (Syndromus) haematostomus [sic]. Lehmann and Maassen 2004: 20.
Amphidromus attapeuensis Thach & Huber in Thach, 2017: 37–38, figs 573–578. Type locality: Attapeu Province, southeast of Laos, close to Vietnam border. Thach 2020a: 51, 52. Thach 2021: 55.

##### Material examined.

Laos: Sinistral, ***lectotype*** of “Amphidromushaematostomavar.viridis”, SMF 7559/1 (Fig. 24A); sinistral, ***lectotype*** of “Amphidromushaematostomavar.varians”, SMF 7561/1 (Fig. 24B); sinistral, ***holotype*** of “*Amphidromusattapeuensis*”, NHMUK 20170278 (Fig. 24C).

##### Other material examined.

Laos: 5S specimens, Xe Pian village, Paksong District, Champasak Province, CUMZ 10217 (Inkhavilay *et al.* 2019: fig. 44a); two lots in W.J.M. Maassen Collection (8S specimens and 14S specimens), Boloven Plateau, Paksong District, Champasak; 4S specimens, Samphanh District, Phongsali Province, NMNS-8764-053–NMNS-8764-056 (Fig. 24D); 20S specimens, Ba Chien, Pakse District, Champasak Province, NMNS-8764-057–NMNS-8764-076 (Fig. 24E, F).

Vietnam: 5S specimens, Kbang District, Gia Lai Province, NMNS-8764-077NMNS-8764-081 (Fig. 24G).

##### Diagnosis.

Shell medium and sinistral. Parietal callus, lip and columella with bright to dark rose-pink. Varix sometimes present. Genitalia without appendix.

##### Differential diagnosis.

*Amphidromushaematostoma* differs from the similar sinistral species *A.madelineae* in having a whitish apex, slightly thickened parietal callus with pale to dark rose-pink colouration, while *A.madelineae* has tinted pink ~ 1–2 whorls from apex, and thin transparent parietal callus. This species also differs from the similar *A.roseolabiatus* in that the latter has a chirally dimorphic shell, a whitish apex and the genitalia with a very long appendix. The molecular phylogeny in this study reveals that *A.haematostoma* is a distinct clade from its sister *A.madelineae* (Fig. 2). The COI and 16S *p*-distances between *A.haematostoma* and *A.madelineae* are 13.93% and 6.04%, respectively (Table 2).

##### Description.

***Shell*** medium (height 23.8–35.4 mm, width 13.3–21.0 mm), sinistral, ovate conical, rather thin and glossy. Spire elongate conical; apex acute, without black spot on tip, and earlier whorls whitish. Whorls 6–7 convex to smooth; suture wide and depressed; last whorl well rounded. Periostracum thick corneous or with green to greenish yellow colour; varix occasionally present. Shell ground colour white or yellowish colour (without periostracum); dark yellow subsutural band and a band at around umbilicus usually present (rarely indistinguishable). Parietal callus thickened with bright to dark rose-pink colour. Aperture broadly ovate and inner side of outer wall whitish; peristome little thickened, expanded, and weakly reflexed but not attached to last whorl; lip bright to dark rose-pink colour and with little darker colour at the edge. Columella bright to dark rose-pink colour, straight, or little twisted. Umbilicus imperforate.

***Genital organs*.** Atrium relatively short. Penis slender, conical, and short ~ 1/3 of vaginal length. Penial retractor muscle thickened, long and inserting on epiphallus close to penis. Epiphallus stout tube and approximately as long as vagina. Flagellum short, extending from epiphallus and terminating in curved tip; appendix absent. Vas deferens slender tube passing from free oviduct and terminating at epiphallus-flagellum junction (Fig. 25A). Internal wall of penis corrugated, exhibiting prominent series of thickened and swollen longitudinal penial pilasters forming fringe around penial wall, and with fine and weak folds around base of penial verge. Penial verge very short, with smooth surface, and opening at the tip (Fig. 25B).

Vagina slender, long cylindrical, and ~ 2× longer than penis. Gametolytic duct cylindrical tube then gradually tapering to slender tube terminally and connected to gametolytic sac (missing during dissection). Free oviduct short; oviduct compact, forming lobule alveoli (Fig. 25A). Internal wall of vagina possessing corrugated ridges near genital orifice; ridges becoming smooth longitudinal vaginal pilasters in middle, swollen with irregularly shaped deep crenelations close to free oviduct opening (Fig. 25B).

##### Distribution.

This species has a wide distribution range covering Attapeu, Champasak, and Phongsali provinces, Laos, and Gia Lai Province, Vietnam.

##### Remarks.

A degree of shell colour variation occurs in the specimens from Pakse, Champasak, Laos (Fig. 24E, F) in having yellowish to golden-yellow periostracum, indistinct subsutural band and a dark rose-pink apertural lip. In addition, the type specimens and recently collected specimens from Kbang, Gia Lai, Vietnam (Fig. 24B, C, G) tend to have broad brownish radial bands on the earlier spire whorls.

This species also exhibits a prominent population genetic structure, where some clades constitute only the specimens from the same collecting locality (Fig. 26). The COI intraspecific distance among all *A.haematostoma* specimens is 10.03%, which is the highest distance of all *Amphidromus* species in this study. This value is higher than twice the optimum intra/interspecific threshold value of 4% for stylommatophoran land snails (Davison et al. 2009). However, as all specimens have congruent morphology as stated above, we refrain from treating each pool of samples from the same collecting locality as a distinct taxon, before more specimens from each locality are critically examined.

#### ﻿﻿ Amphidromus madelineae

Taxon classificationAnimaliaStylommatophoraCamaenidae

Thach, 2020

164E7B26-13FD-5EB7-9DCA-0E1DD0C325FB

Figs 22B , 24H–M , 25C, D , 26

Amphidromus madelineae Thach, 2020a: 68–69, pl. 48, figs 592, 593; pl. 49 figs 594–596. Type locality: Quang Nam Province, Central Vietnam. Thach 2021: 68.

##### Material examined.

Vietnam: Sinistral, ***holotype*** of “*Amphidromusmadelineae*”, MNHN-IM-2000-35566 (Fig. 24H).

##### Other material examined.

Vietnam: 5S specimens, Duy Xuyen District, Quang Nam Province, NMNS-8764-108–NMNS-8764-112 (Fig. 24I, J); 10S specimens, Za Hung, Dong Giang District, Quang Nam Province, NMNS-8764-113–NMNS-8764-122 (Fig. 24K–M).

##### Diagnosis.

Shell small to medium, sinistral; apex tinted pink to purplish pink. Parietal callus transparent; lip whitish to purplish pink; columella and inner side of outer wall around columella purplish pink. Genitalia without appendix.

##### Differential diagnosis.

*Amphidromusmadelineae* differs from the similar sinistral species *A.haematostoma* in having tinted-pink colour ~ 1–2 whorls from apex, and thin and transparent parietal callus, while *A.haematostoma* has a whitish apex, slightly thickened parietal callus with pale to dark rose-pink colour. This species also differs from the similar *A.roseolabiatus* in that the latter has a chirally dimorphic shell, a whitish apex and the genitalia with a very long appendix. The molecular phylogeny in this study reveals that *A.madelineae* is a distinct clade from its sister *A.haematostoma* (Figs 2, 26). The COI and 16S *p*-distances between *A.madelineae* and *A.haematostoma* are 13.93% and 6.04%, respectively (Table 2).

##### Description.

***Shell*** small to medium (height 27.7–38.0 mm, width 16.2–20.2 mm), sinistral, elongate to ovate conical, rather thin and glossy. Spire conical; apex acute, tinted pink to purplish pink and without black spot on tip. Whorls 5–6 nearly smooth; suture wide and shallow; last whorl rounded to nearly globose. Periostracum usually deciduous to yellowish green radial streaks, more conspicuous on last whorl and faded in earlier whorls. Last whorl with thin, dark green subsutural band, sometimes with irregular greenish spiral blotched bands below periphery; varix sometimes present. Parietal callus thin and transparent. Aperture ovate to elongate; peristome little thickened and expanded; lip generally whitish to purplish pink; inner side of outer wall whitish around columella with purplish pink colour. Columella straight, thickened and pale to dark purplish pink. Umbilicus imperforate.

***Radula*.** Teeth arranged in anteriorly pointed V-shaped rows. Central tooth monocuspid and spatulate with truncated cusp. Lateral teeth bicuspid; endocone small, slightly elongate, with wide and deep notch, and dull cusp; ectocone large with slightly blunt to dull cusp. Lateral teeth gradually transformed to asymmetric tricuspid marginal teeth. Outermost teeth with small and multicuspid (Fig. 22B).

***Genital organs*.** Atrium relatively short. Penis stout, cylindrical, and short, ~ 1/2 as long as vagina. Penial retractor muscle thickened, short and inserting on epiphallus close to penis. Epiphallus stout tube and approximately as long as vagina. Flagellum short, extending from epiphallus and terminating in slightly curved tip; appendix absent. Vas deferens slender tube passing from free oviduct and terminating at epiphallus-flagellum junction (Fig. 25C). Internal wall of penis corrugated, exhibiting prominent series of thickened and smooth surfaced longitudinal penial pilasters forming fringe around penial wall. Penial verge very short and with smooth surface (Fig. 25D).

Vagina long cylindrical, and ~ 2× longer than penis. Gametolytic duct long cylindrical tube then gradually tapering to slender tube terminally and connected to bulbus gametolytic sac. Free oviduct short; oviduct compact, forming lobule alveoli (Fig. 25C). Internal wall of vagina possessing slightly corrugated ridges near genital orifice; ridges becoming roughly irregular vaginal pilasters in middle and close to free oviduct opening (Fig. 25D).

##### Distribution.

This species is found in Quang Nam Province, Vietnam.

##### Remarks.

Specimens from Za Hung, Dong Giang, Quang Nam, Vietnam (Fig. 24L, M) are superficially similar to *A.haematostoma* in having greenish shell colour and a purplish pink lip.

#### ﻿﻿ Amphidromus costifer

Taxon classificationAnimaliaStylommatophoraCamaenidae

Smith, 1893

C7720456-EFEB-58BE-A5DD-50F100978EF5

**Figure 27.**
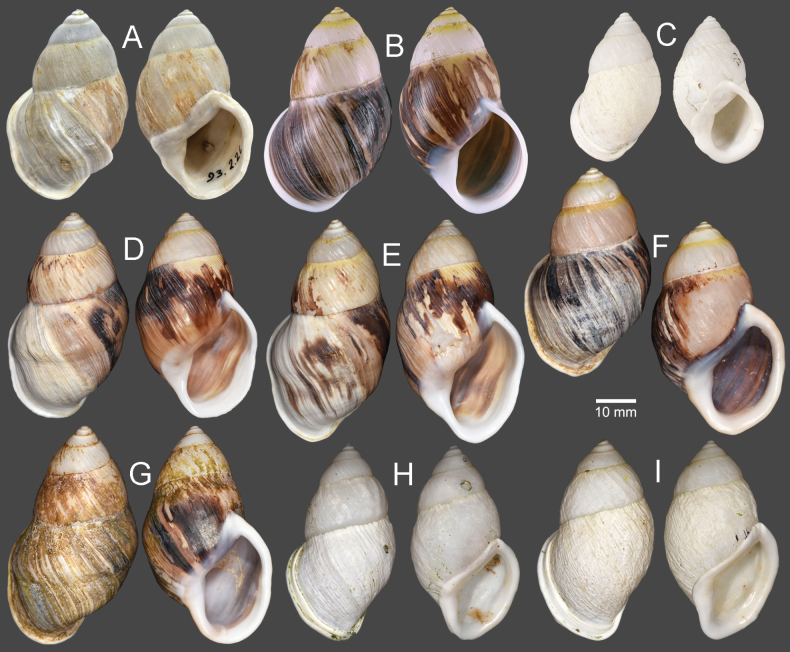
Shells of *Amphidromuscostifer* Smith, 1893 **A** lectotype of “*Amphidromuscostifer*” (NHMUK 1893.2.26.4) **B** holotype of “*Amphidromuscostifergemmalimae*” (MNHN-IM-2000-35550) **C** holotype of “*Amphidromusnguyenkhoai*” (MNHN-IM-2000-35569) **D, E** specimens from Tay Son, Binh Dinh, Vietnam (NMNS-8764-035, NMNS-8764-040) **F, G** specimens from Ea Sup, Dak Lak, Vietnam (NMNS-8764-047, NMNS-8764-048) **H, I** specimens from An Lao, Binh Dinh, Vietnam (NMNS-8764-051, NMNS-8764-052). Credit: H. Taylor, NHM (**A**), P. Bourguignon, MNHN (**B**), A. Lardeur (**C**).

**Figure 28.**
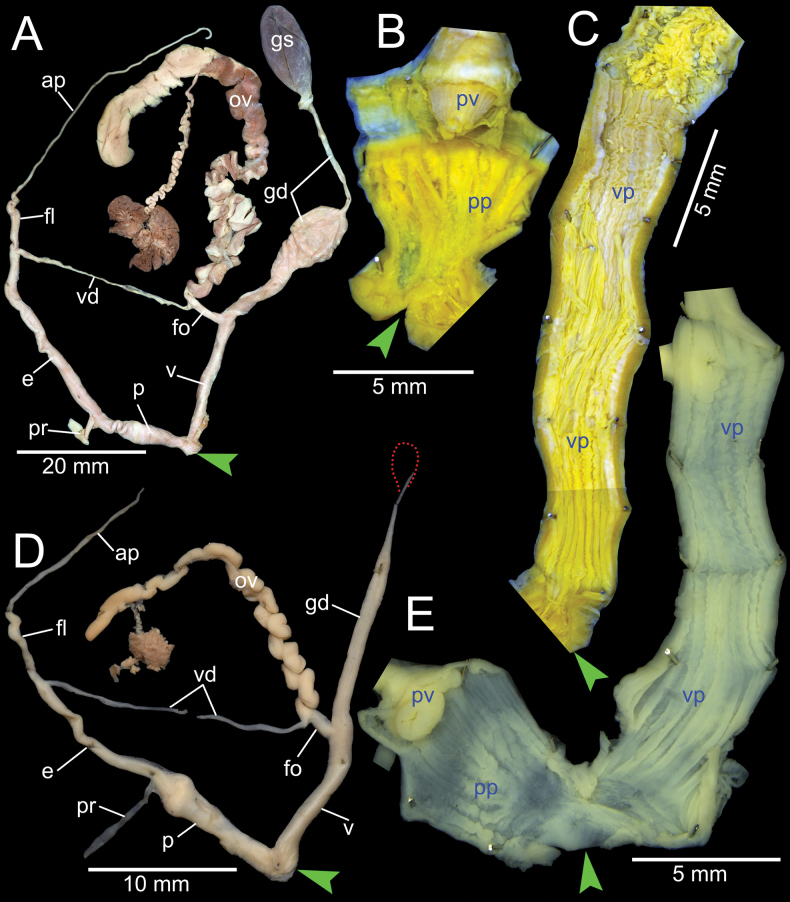
Genitalia of *Amphidromus* spp **A–C***Amphidromuscostifer* Smith, 1893 from Ea Sup, Dak Lak, Vietnam (NMNS-8764-048), showing **A** general view of genitalia **B** interior structures of penis **C** interior structures of vagina chamber **D, E***Amphidromuspankowskianus* Thach, 2020 from Khammouan Province, Laos, near Minh Hoa District, Quang Binh Province, Vietnam (NMNS-8764-152), showing **D** general view of genitalia **E** interior structures of penis and vagina chamber. Red dots indicate the shape of the missing gametolytic sac. Green arrows indicate the genital openings. Abbreviations: ap, appendix; e, epiphallus; fl, ﬂagellum; fo, free oviduct; gd, gametolytic duct; gs, gametolytic sac; ov, oviduct; p, penis; pp, penial pilaster; pr, penial retractor muscle; pv, penial verge; v, vagina; vd, vas deferens; vp, vaginal pilaster.

**Figure 29.**
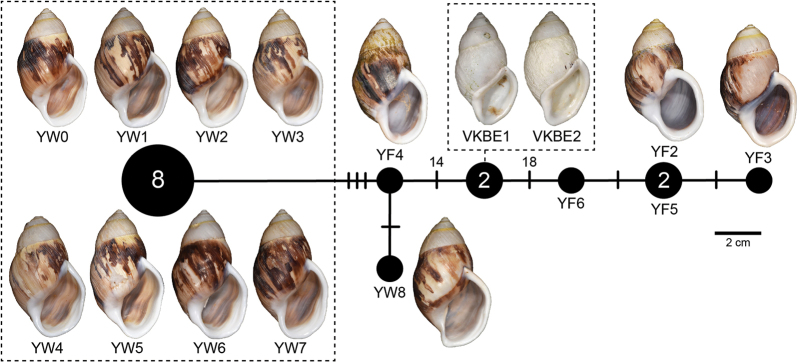
Mitochondrial 16S haplotype minimum spanning networks of *Amphidromuscostifer* Smith, 1893. The size of each circle corresponds to the frequency of that haplotype, also shown as the number in that circle. The bars on the branches indicate the number of mutational steps between haplotypes. Specimen codes correspond to those in Table 1.

Figs 22C , 27 , 28A–C , 29

Amphidromus costifer Smith, 1893: 12, text fig. Type locality: dans les Montagnes boitées du Huyen de Tri-phuoc, Province Binh-dinh, An-nam [in the Huyen Mountains of Tri-phuoc, Binh-dinh Province, An-nam]. Fulton 1896: 91, pl. 7, fig. 6, 6a. Möllendorff 1898: 75. Pilsbry 1900: 176–177, pl. 59, figs 22, 23. Fischer and Dautzenberg 1904: 405. Laidlaw and Solem 1961: 590, 592, 613, fig. 40a, b. Schileyko 2011: 50. Sutcharit et al. 2015: 65, fig. 6c. Thach 2020a: pl. 46, figs 560, 561.
Amphidromus costifer gemmalimae Thach, 2020a: 55, pl. 45, figs 551–557. Type locality: Krong Nang, Dak Lak Province, Central Vietnam. Thach 2021: 58. syn. nov.
Amphidromus nguyenkhoai Thach, 2020a: 71, pl. 64, figs 776–784. Type locality: Krong Pa District, Gia Lai, Central Vietnam. Thach 2021: 71. syn. nov.

##### Material examined.

Vietnam: Dextral, ***lectotype*** of “*Amphidromuscostifer*”, NHMUK 1893.2.26.4 (Fig. 27A); dextral, ***holotype*** of “*Amphidromuscostifergemmalimae*”, MNHN-IM-2000-35550 (Fig. 27B); dextral, ***holotype*** of “*Amphidromusnguyenkhoai*”, MNHN-IM-2000-35569 (Fig. 27C).

##### Other material examined.

Vietnam: 10D specimens, Tay Son District, Binh Dinh Province, NMNS-8764-035–NMNS-8764-044 (Fig. 27D, E); 6D specimens, Ea Sup District, Dak Lak Province, NMNS-8764-045–NMNS-8764-050 (Fig. 27F, G); 2D specimens, An Lao District, Binh Dinh Province, NMNS-8764-051, NMNS-8764-052 (Fig. 27H, I).

##### Diagnosis.

Shell large, dextral, and spire ovate conical. Shell surface with prominent irregular growth lines or prominent crests of expanded lip. Aperture broadly ovate or truncate. Genitalia with appendix.

##### Differential diagnosis.

*Amphidromuscostifer* is unique among all reported Vietnamese species (Schileyko 2011) in having a large, dextral shell with an ovate conical spire, and the shell surface with prominent irregular growth lines or prominent crests of expanded lip. *Amphidromuscostifer* is also recognised by a distinct clade in the molecular phylogeny (Fig. 2), with the closest *p*-distance to *A.metabletus* in COI (16.63%) and *A.buelowi* in 16S (13.44%) (Table 2).

##### Description.

***Shell*** large (height 48.9–59.7 mm, width 27.3–34.8 mm), dextral, solid, and ovate conical shape. Spire ovate conical; apex acute without black spot on tip. Whorls 5–7 little convex; suture wide and shallow; last whorl large, rounded to slightly ovate. Periostracum brownish to thin corneous; strong varix usually absent. Shell surface: spire generally with prominent irregular growth lines or with weak radial streak; last whorl with strong irregular growth lines, coarse or with prominent radial ridges, and usually prominent crest of expanded lip present. Shell colour highly variable: spire generally uniform whitish to yellowish (pale yellowish subsutural band detectable); last whorl has no pattern but usually stained with dark to dark brown blotches, smear or radial streaks. Parietal callus thickened and white, and broadly dilated at umbilical area. Aperture broadly ovate or truncate (sometimes irregular); inner side of outer wall generally whitish to yellowish. Peristome thickened, expanded, and slightly reflexed; lip whitish. Columella white and straight. Umbilicus imperforate.

***Radula*.** Teeth arranged in anteriorly pointed V-shaped rows. Central tooth monocuspid and spatulate with truncated cusp. Lateral teeth bicuspid; endocone slightly curved with wide notch and curved cusp; ectocone large with truncated to blunt cusp. Lateral teeth gradually transformed to asymmetric tricuspid marginal teeth. Outermost teeth with tiny ectocone; endocone and mesocone large with curved cusps (Fig. 22C).

***Genital organs*.** Atrium relatively short. Penis enlarged, conical, and nearly 1/2 as long as vagina. Penial retractor muscle thickened and inserting on epiphallus close to penis. Epiphallus long and slender tube. Flagellum short, extending from epiphallus, approximately as long as penis, and terminating in slightly enlarged coil. Appendix short, slender tube, 3×longer than flagellum and approximately as long as epiphallus. Vas deferens slender tube passing from free oviduct and terminating at epiphallus-flagellum junction (Fig. 28A). Internal wall of penis corrugated, exhibiting series of thickened longitudinal penial pilasters forming fringe around penial wall, and with smooth wall around base of penial verge. Penial verge short conical with thin longitudinal ridges surface, and with opening at the tip (Fig. 28B).

Vagina slender, long cylindrical, and ~ 2× longer than penis. Gametolytic duct cylindrical tube, extremely enlarged then abruptly tapering to slender tube terminally and connected to enlarged elliptical gametolytic sac. Free oviduct short; oviduct compact, enlarged to form lobule alveoli (Fig. 28A). Internal wall of vagina possessing corrugated ridges near genital orifice; ridges becoming swollen and smooth longitudinal vaginal pilasters in middle, and irregular shaped and deep crenelations close to free oviduct opening (Fig. 28C).

##### Haplotype network.

There was a total of seven 16S haplotypes (Fig. 29) of *A.costifer* in this study, and the highest numbers of mutational steps in the 16S minimum spanning networks are 18.

##### Distribution.

The distribution range of the species covers Binh Dinh, Dak Lak, and Gia Lai provinces, Vietnam.

##### Remarks.

As Smith (1893) did not explicitly designate a type, and stated that a total of seven specimens were examined, the indication of the holotype in Sutcharit et al. (2015) is thus incorrect. Therefore, the syntype of “*Amphidromuscostifer*” NHMUK 1893.2.26.4 is hereby designated as the lectotype.

Our recent specimens with a monochrome whitish shell identical to the holotype of *A.nguyenkhoai* were found to belong to the same clade as the typical *A.costifer*, with 14–18 mutational steps to the other specimens in the 16S haplotype network (Fig. 29). In addition, upon examining the type specimens of *A.costifer* and *A.nguyenkhoai*, except for the shell colour, the holotype of *A.nguyenkhoai* agrees well with the lectotype of *A.costifer* in terms of shell shape, shell surface, peristome, and apertural shape. Thus, *A.nguyenkhoai* is regarded herein as a junior subjective synonym of *A.costifer*.

The subspecies *A.costifergemmalimae* was described as distinct from the nominotypical subspecies in having a stouter shell shape, smoother, not well-defined and not strongly calloused parietal wall, axial ribs with regular strength, a regularly convex outer rib, a completely closed umbilicus, and a columella not widening laterally (Thach 2020a). However, these characters fall within the intraspecific variations shown in *A.costifer* clade. Thus, *A.costifergemmalimae* is also regarded herein as a junior subjective synonym of *A.costifer*.

Additional shell variations, which occur in the monochrome whitish specimens from An Lao, Binh Dinh, Vietnam (Fig. 27C, H, I), are the occurrence of strongly thickened parietal callus, a thickened, multi-layered and broadly expanded apertural lip, and the shell surface much coarser with irregular growth lines and malleated pits.

The COI intraspecific distance among all *A.costifer* specimens is 7.84%, which is the second highest distance of all *Amphidromus* species in this study. This value is higher than the optimum intra/interspecific threshold value of 4% for stylommatophoran land snails (Davison et al. 2009). In addition, the 16S intraspecific distance among all *A.costifer* specimens is 3.39%, which is the highest distance of all *Amphidromus* species in this study, and the 16S haplotype network also exhibits a prominent population genetic structure (Fig. 29). However, as all specimens have congruent morphology as stated above, we refrain from treating each pool of samples from the same collecting locality as a distinct taxon, before more specimens from each locality are critically examined.

#### ﻿﻿ Amphidromus roseolabiatus

Taxon classificationAnimaliaStylommatophoraCamaenidae

Fulton, 1896

EE030428-5DA7-51C6-BCD9-1642F7D44CBB

Figs 30A–F , 31

Amphidromus roseolabiatus Fulton, 1896: 89, pl. 6, fig. 8. Type locality: Siam [Thailand]. Pilsbry 1900: 188, pl. 60, fig. 36. Fischer and Dautzenberg 1904: 407. Laidlaw and Solem 1961: 527, 655. Richardson 1985: 42. Schileyko 2011: 51. Sutcharit et al. 2015: 88, fig. 13j, k. Inkhavilay et al. 2019: 94, figs 45d–f, 58a. Páll-Gergely et al. 2020: 54. Thach 2020b: 360, fig. 7.
Amphidromus (Amphidromus) roseolabiatus. Inkhavilay et al. 2017: 3, 6, 9, 10, figs 2a, b, 3a, b, 4a−f, 6a, b, 7a−c.
Amphidromus phuonglinhae Thach, 2017: 45, pl. 46, figs 581–584. Type locality: Bo Trach District, Quang Binh Province, Central Vietnam. Thach 2021: 76.

##### Material examined.

**Figure 30.**
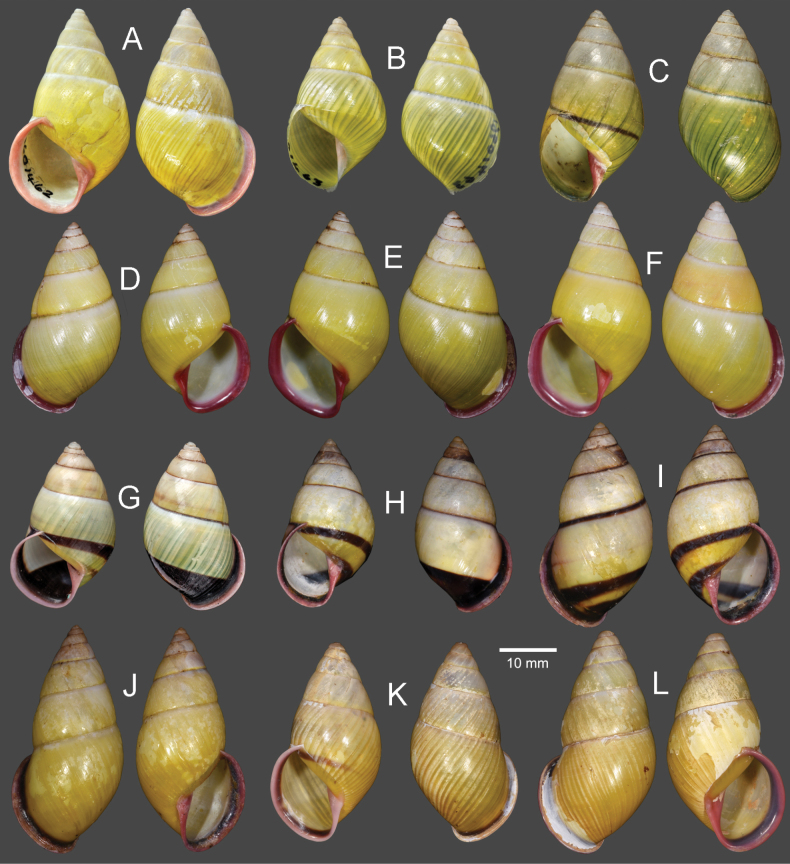
Shells of *Amphidromus* spp **A–F***Amphidromusroseolabiatus* Fulton, 1896 **A** lectotype of “*Amphidromusroseolabiatus*” (NHMUK 19601462) **B** paralectotype of “*Amphidromusroseolabiatus*” (NHMUK 19601463) **C** holotype of “*Amphidromusphuonglinhae*” (MNHN-IM-2000-33200) **D–F** specimens from Kampong Siem, Kampong Cham, Cambodia (NMNS-8764-254, NMNS-8764-258, NMNS-8764-260) **G–L***Amphidromuspankowskianus* Thach, 2020 **G** holotype (NHMUK 20200213) **H–J** specimens from Lak Sao, Khamkeut, Bolikhamsai, Laos (NMNS-8764-154, NMNS-8764-170, NMNS-8764-192) **K, L** specimens from Khammouan Province, Laos, near Minh Hoa District, Quang Binh Province, Vietnam (NMNS-8764-150, NMNS-8764-151). Credit: M. Caballer (**C**), K. Webb (**G**).

Thailand: Sinistral, ***lectotype*** of “*Amphidromusroseolabiatus*”, NHMUK 19601462 (Fig. 30A); 1S, paralectotype of “*Amphidromusroseolabiatus*”, NHMUK 19601463 (Fig. 30B).

Vietnam: Sinistral, ***holotype*** of “*Amphidromusphuonglinhae*”, MNHN-IM-2000-33200 (Fig. 30C).

##### Other material examined.

Cambodia: 4D + 6S specimens, Kampong Siem District, Kampong Cham Province, NMNS-8764-254–NMNS-8764-263 (Fig. 30D–F).

##### Diagnosis.

Shell medium and chirally dimorphic. Parietal callus transparent; lip and columella purplish pink. Genitalia with appendix.

##### Differential diagnosis.

This species is very closely similar to *A.pankowskianus* in terms of shell morphology and colour pattern. However, this species lacks a dark radial band behind the reflected lip which is also visible in the inner side of the shell, and a dark spiral band below periphery, both of which are present in *A.pankowskianus*. *Amphidromusroseolabiatus* differs from the similar species *A.madelineae* and *A.haematostoma* in having a chirally dimorphic shell, and genitalia with a very long appendix, while both *A.madelineae* and *A.haematostoma* are exclusively sinistral, and the genitalia lacks an appendix. *Amphidromusroseolabiatus* also differs from both *A.smithi* Fulton, 1896 and *A.ventrosulus* Möllendorff, 1900 from Vietnam (Zilch 1953; Sutcharit et al. 2015) in having a chirally dimorphic shell, a purplish pink lip and fine green streaks. In contrast, *A.smithi* has a sinistral shell, a dark red to brownish lip with dark spot on the apex, and *A.ventrosulus* has a sinistral shell, uniform green colour, elongate spire and more depressed suture. The molecular phylogeny in this study reveals that *A.roseolabiatus* is a distinct clade from its sister *A.pankowskianus* (Fig. 2). The COI and 16S *p*-distances between *A.roseolabiatus* and *A.pankowskianus* are 13.02% and 6.14%, respectively (Table 2).

##### Description.

***Shell*** medium (height 33.1–38.6 mm, width 19.2–21.6 mm), chirally dimorphic, elongate to ovate conical, rather thin and glossy. Spire conical; apex acute, light brown and without black spot on tip. Whorls 6–7 nearly smooth; suture wide and depressed; last whorl rounded. Periostracum usually deciduous to yellowish green radial streaks, more conspicuous on last whorl and faded in earlier whorls. Last whorl with thin, whitish subsutural band; with or without reddish brown spiral band on periphery but usually not reaching apertural lip; varix absent. Parietal callus thin and transparent. Aperture ovate to elongate; peristome expanded and not reflected; lip usually purplish pink. Columella straight, thickened, purplish pink. Umbilicus narrowly opened.

##### Haplotype network.

**Figure 31.**
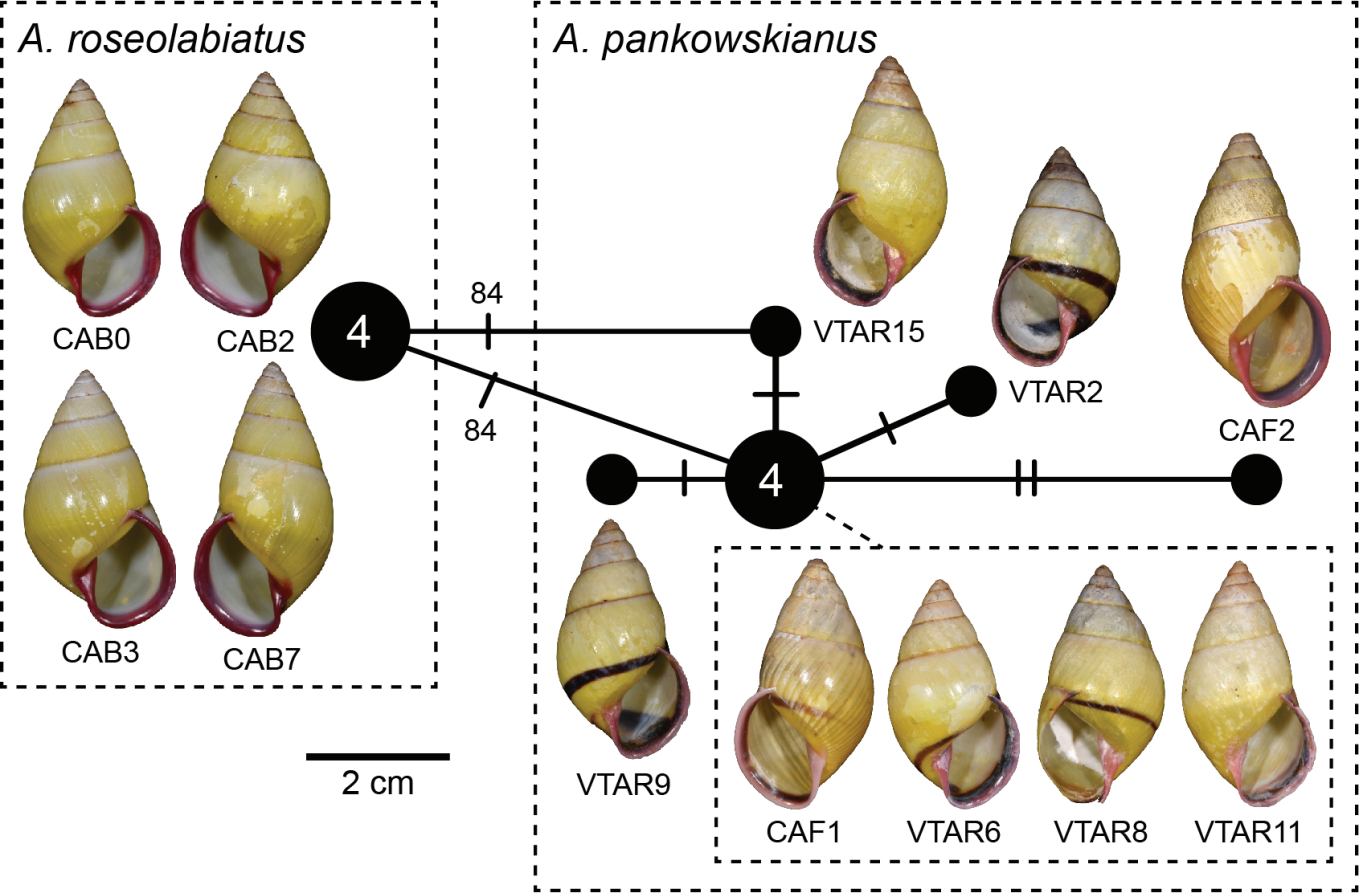
Mitochondrial COI haplotype minimum spanning networks of *Amphidromusroseolabiatus* Fulton, 1896 and *Amphidromuspankowskianus* Thach, 2020. The size of each circle corresponds to the frequency of that haplotype, also shown as the number in that circle. The bars on the branches indicate the number of mutational steps between haplotypes. Specimen codes correspond to those in Table 1.

There was one COI haplotype of *A.roseolabiatus* in this study (Fig. 31).

##### Distribution.

This species is found in Khammouan Province, Laos, Kampong Cham Province, Cambodia, and Quang Binh Province, Vietnam. The distribution of this species in Bolikhamxay, Laos according to Inkhavilay et al. (2017) is dubious (see below).

##### Remarks.

Inkhavilay et al. (2017) also included the white-lipped morph from Bolikhamxay, Laos in *A.roseolabiatus*. However, in this study we only incorporated the typical purplish pink-lipped morph in the phylogenetic analyses. Therefore, the identification of the white-lipped morph from Bolikhamxay, Laos as *A.roseolabiatus* or another distinct species remains to be further investigated.

#### ﻿﻿ Amphidromus pankowskianus

Taxon classificationAnimaliaStylommatophoraCamaenidae

Thach, 2020

512D5B0C-D3D7-508F-B535-F75DFD18BCA4

Figs 28D, E , 30G–L , 31

Amphidromus pankowskiana [sic] Thach, 2020a: 72–73, pl. 48, figs 582–586. Type locality: Northwestern District of Khanh Hoa Province, Central Vietnam.
Amphidromus pankowskianus. Thach 2021: 72.

##### Material examined.

Vietnam: ***Holotype***, NHMUK 20200213 (Fig. 30G).

##### Other material examined.

Laos: 2D + 1S specimens, Khammouan Province, near Minh Hoa District, Quang Binh Province, Vietnam, NMNS-8764-150–NMNS-8764-152 (Fig. 30K, L); 23D + 17S specimens, Lak Sao, Khamkeut District, Bolikhamsai Province, NMNS-8764-153–NMNS-8764-191, NMNS-8764-212 (Fig. 30H–J).

##### Diagnosis.

Shell medium and chirally dimorphic. Last whorl without or with narrow to spiral band on periphery. Parietal callus transparent; lip and columella pale purplish pink; dark radial band on palatal wall. Genitalia with appendix.

##### Differential diagnosis.

This species is very closely similar to *A.roseolabiatus* in terms of shell morphology and colour pattern. However, this species is distinct in having a dark radial band behind the reflected lip which is also visible in the inner side of the shell and sometimes with a dark spiral band below periphery. In addition, this species also differs from *A.haematostoma* and *A.madelineae* in having a chirally dimorphic shell, with dark radial bands behind the expanded lip, and the genitalia with a long flagellum. The molecular phylogeny in this study reveals that *A.pankowskianus* constitutes its own distinct clade which is sister to *A.roseolabiatus* (Fig. 2). The COI and 16S *p*-distances between *A.pankowskianus* and *A.roseolabiatus* are 13.02% and 6.14%, respectively (Table 2).

##### Description.

***Shell*** medium (height 30.8–39.9 mm, width 17.2–19.2 mm), chirally dimorphic, elongate to ovate conical, rather thin and glossy. Spire conical; apex acute, light brown and without black spot on tip. Whorls 6–7 nearly smooth; suture wide and depressed; last whorl rounded. Periostracum usually deciduous to yellowish green radial streaks, more conspicuous on last whorl and faded in earlier whorls. Last whorl without or with narrow to wide brownish spiral band on periphery; varix absent. Parietal callus thin and transparent. Aperture ovate; peristome expanded and not reflected; lip pale purplish pink. Outer palatal wall with dark radial band just next to expanded lip (also visible on inner wall) and brownish radial band encircled umbilicus present (sometimes absent). Umbilicus narrowly opened.

***Genital organs*.** Atrium relatively short. Penis enlarged, conical, and almost as long as vagina. Penial retractor muscle thin and inserting on epiphallus close to penis. Epiphallus thin and long slender tube, and approximately as long as penis. Flagellum short, extending from epiphallus, ~ 1/2 of penis length, and terminating in slightly enlarged coil. Appendix short, slender tube, nearly as long as epiphallus. Vas deferens slender tube passing from free oviduct and terminating at epiphallus-flagellum junction (Fig. 28D). Internal wall of penis corrugated, exhibiting series of weak longitudinal penial pilasters forming fringe around penial wall, and with smooth wall around base of penial verge. Penial verge very short conical with opening at the tip (Fig. 28E).

Vagina slender, cylindrical, and approximately as long as penis. Gametolytic duct cylindrical tube, similar diameter as vagina then tapering to slender tube terminally and connected to enlarged elliptical gametolytic sac (missing during dissection). Free oviduct short; oviduct forming lobule alveoli (Fig. 28D). Internal wall of vagina possessing smooth ridges near genital orifice; ridges becoming swollen and corrugated longitudinal vaginal pilasters in middle, and with deep crenelations close to free oviduct opening (Fig. 28E).

##### Haplotype network.

There was a total of five COI haplotypes of *A.pankowskianus* in this study, and the highest number of mutational steps in the COI minimum spanning network is two (Fig. 31).

##### Distribution.

This species is found in Bolikhamsai and Khammouan provinces, Laos, and Khanh Hoa Province, Vietnam.

##### Remarks.

Empty shells from Phong Nha National Park, Quang Binh Province, Vietnam, identified as ‘*A.roseolabiatus*’ in Inkhavilay et al (2017: CUMZ 7053; 2D+3S shells) possess a transparent parietal callus with a dark radial band on the palatal wall just next to the lip. This specimen lot could probably be assigned to *A.pankowskianus* instead. Future molecular evidence is needed to shed light on the systematic status of this population.

## ﻿﻿Discussion

Arboreal snails in the genus *Amphidromus* exhibit high levels of variation in intraspecific shell colour and pattern (Haniel 1921; Lee et al. 2022), while shells of different species may be similar due to shared arboreal adaptations (Jirapatrasilp et al. 2022; Lee et al. 2022). Although conchological characters can be used to diagnose different *Amphidromus* species to some extent (Laidlaw and Solem 1961; Sutcharit and Panha 2006; Inkhavilay et al. 2019), the amount of intraspecific shell variability is most often not, or poorly, known. In this regard, DNA sequence data, especially the mitochondrial gene fragments referred to as “DNA barcodes,” provide additional and solid evidence to delimit species and help to distinguish between intra- and interspecific shell differentiation (Pholyotha et al. 2021; Jirapatrasilp et al. 2022; Lee et al. 2022).

Apart from examining the reciprocal monophyly of each species, the use of interspecific genetic distances is another means to set the preliminary cut-off for each clade to become putative species, although the use of interspecific genetic distances has been discussed as an unfavourable way to delimit species (Ferguson 2002). Davison et al. (2009) reported an optimal COI intra/interspecific threshold value for stylommatophoran land snails as 4%, although this value was associated with an overall false negative error (interspecific variation misdiagnosed as same species) of 32% and 44% for the longer (381 bp) and shorter (228 bp) sequences, respectively. In our study, the species retrieved from the reciprocal monophyly validated by this cut-off value are in accordance with the preliminarily retrieved morphospecies, and we identify a range of 9–12% as the COI interspecific threshold value for *Amphidromus* species in this study. Although Davison et al. (2009) did not conclusively identify a barcode gap in stylommatophoran land snails, our intraspecific distances of *Amphidromus* species typically fall below or hover around 5%. The notable exceptions are *A.haematostoma* (10.03%) and *A.costifer* (7.84%), in which these two species would be flagged for further examination of possible cryptic species. Therefore, we propose a COI barcode gap for *Amphidromus* of 5–9%, which could be used to delimit more *Amphidromus* species in further studies.

Although there is still no general 16S intra/interspecific threshold value for stylommatophoran land snails, we could estimate the threshold for the Camaenidae to some extent. In this study, we identify a range of 3–5% as the 16S interspecific threshold value for *Amphidromus*. This range is comparable to the lower boundary of 16S interspecific distances reported in other genera in the Camaenidae, e.g., *Aegistohadra* from China and Vietnam (5.97–11.86%; Jirapatrasilp et al. 2022), *Camaena* from China (5–15%; Ding et al. 2016), *Euhadra* from Bonin Islands, Japan (5.8–16.5%; Chiba 1999), and *Acusta* from East Asia (5.3–18.8% Hwang et al. 2021). The 16S barcode gap for *Amphidromus* in this study is less conspicuous, as the 16S intraspecific distances typically fall below or hover around 3%. Therefore, we suggest a 16S interspecific threshold range of 5–6% for the Camaenidae, which could be implemented to support the species delimited by the COI barcode gap. The phylogenetic tree constructed from 16S also yielded identical clades to those from the COI phylogeny.

Internal morphological characters, especially those of reproductive system such as penis and vagina, are often species-specific and therefore are interpreted as the prime species recognition characters (Gómez 2001). The differentiation of these characters has been assumed to promote speciation in land snails (Kameda et al. 2009; Sauer and Hausdorf 2009). In the present study we observed some modest differences among *Amphidromus* species with respect to the size, shape, and surface of the penial verge, and the inner wall sculpture of the penis and vagina. Further morphometric analyses of several parts of genitalia (Kameda et al. 2009; Sauer and Hausdorf 2009) will shed light on the extent of divergence in genital morphology of these *Amphidromus* species.

The non-monophyly of *Amphidromus* species exhibiting the same chirality state (exclusively sinistral or dextral, or chirally dimorphic) illustrates that the multiple origins of left–right coiling reversal are common in terrestrial snails (Schilthuizen and Davison 2005; Gittenberger et al. 2012). Within the family Camaenidae, shell coiling reversal has also been reported in *Satsuma* Adams, 1868 (Hoso et al. 2010) and *Aegistohadra* Wu, 2004 (Jirapatrasilp et al. 2022). *Amphidromus* is also well-known for a high number of species exhibiting dimorphic chirality (Schilthuizen et al. 2005; Sutcharit et al. 2007, 2013), and the coexistence of both shell coiling directions in the same population has been assumed to be maintained by sexual selection (Schilthuizen et al. 2007, 2012). *Amphidromus* thus has an important role in chirality research, which would further support the importance of the systematic revision of this snail group.

## Supplementary Material

XML Treatment for Amphidromus

XML Treatment for Amphidromus ingens

XML Treatment for Amphidromus asperoides

XML Treatment for Amphidromus bozhii

XML Treatment for Amphidromus placostylus

XML Treatment for Amphidromus ingensoides

XML Treatment for Amphidromus buelowi

XML Treatment for Amphidromus thachi

XML Treatment for Amphidromus metabletus

XML Treatment for Amphidromus haematostoma

XML Treatment for Amphidromus madelineae

XML Treatment for Amphidromus costifer

XML Treatment for Amphidromus roseolabiatus

XML Treatment for Amphidromus pankowskianus

## ﻿Additional information

### Author ORCIDs

Parin Jirapatrasilp https://orcid.org/0000-0002-5591-6724

Chih-Wei Huang https://orcid.org/0000-0002-2921-4294

Chirasak Sutcharit https://orcid.org/0000-0001-7670-9540

Chi-Tse Lee https://orcid.org/0000-0003-2695-0680

### Data availability

All of the data that support the findings of this study are available in the main text or Supplementary Information.

## Supplementary materials

Supplementary material 1 Bayesian phylogenetic tree of *Amphidromus* spp. Data type jpg https://binary.pensoft.net/file/1007850This dataset is made available under the Open Database License (http://opendatacommons.org/licenses/odbl/1.0/). The Open Database License (ODbL) is a license agreement intended to allow users to freely share, modify, and use this Dataset while maintaining this same freedom for others, provided that the original source and author(s) are credited.Parin Jirapatrasilp, Chih-Wei Huang, Chirasak Sutcharit, Chi-Tse Lee
