# ﻿Corrigendum: Addendum to the Acknowledgements: Jirapatrasilp P, Huang C-W, Sutcharit C, Lee C-T (2024) The arboreal snail genus *Amphidromus* Albers, 1850 (Eupulmonata, Camaenidae) of Southeast Asia: 1. Molecular systematics of some Vietnamese species and related species from Cambodia, Indonesia, and Laos. ZooKeys 1196: 15–78. doi: 10.3897/zookeys.1196.112146

**DOI:** 10.3897/zookeys.1209.130464

**Published:** 2024-08-14

**Authors:** Parin Jirapatrasilp, Chih-Wei Huang, Chirasak Sutcharit, Chi-Tse Lee

**Affiliations:** 1 Animal Systematics Research Unit, Department of Biology, Faculty of Science, Chulalongkorn University, Bangkok, Thailand Chulalongkorn University Bangkok Thailand; 2 Leibniz Institute for the Analysis of Biodiversity Change, Zoological Museum, Martin-Luther-King-Platz 3, Hamburg, Germany Leibniz Institute for the Analysis of Biodiversity Change, Zoological Museum Hamburg Germany; 3 School of Life Science, National Taiwan Normal University, Taipei, Taiwan National Taiwan Normal University Taipei Taiwan; 4 Department of Life Sciences, National Chung Hsing University, Taichung, Taiwan National Chung Hsing University Taichung Taiwan

The authors of “The arboreal snail genus *Amphidromus* Albers, 1850 (Eupulmonata, Camaenidae) of Southeast Asia: 1. Molecular systematics of some Vietnamese species and related species from Cambodia, Indonesia, and Laos” missed an acknowledgement to important contributors who collected specimens for this study, and credits to images of living *Amphidromus* specimens in Figure 6. The authors therefore wish to add the following sentence to the Acknowledgements:

We thank Truong Khoa Nguyen, Minh Tuan Nguyen, Ngoc Anh Pham, and K. Vuong for assistance in specimen collection.

The authors also wish to add the credits in the caption of Figure 6 as follows:

Credits: N. A. Pham (**A, C, E–G**), M. T. Nguyen (**B, H**), T. K. Nguyen (**D, I–J**).

We apologize for the previous omission of these important contributors in the Acknowledgements section and the credits in the figure caption.

## ﻿Additional information

### Author ORCIDs

Parin Jirapatrasilp https://orcid.org/0000-0002-5591-6724

Chih-Wei Huang https://orcid.org/0000-0002-2921-4294

Chirasak Sutcharit https://orcid.org/0000-0001-7670-9540

Chi-Tse Lee https://orcid.org/0000-0003-2695-0680

### Data availability

All of the data that support the findings of this study are available in the main text.

