# Supplementary figures and images for: ﻿The arboreal snail genus Amphidromus Albers, 1850 (Eupulmonata, Camaenidae) of Southeast Asia: 1. Molecular systematics of some Vietnamese species and related species from Cambodia, Indonesia, and Laos
Source: Zookeys. 2024 Mar 22;1196:15–78. doi: 10.3897/zookeys.1196.112146 (PMC10980882; doi:10.3897/zookeys.1196.112146)

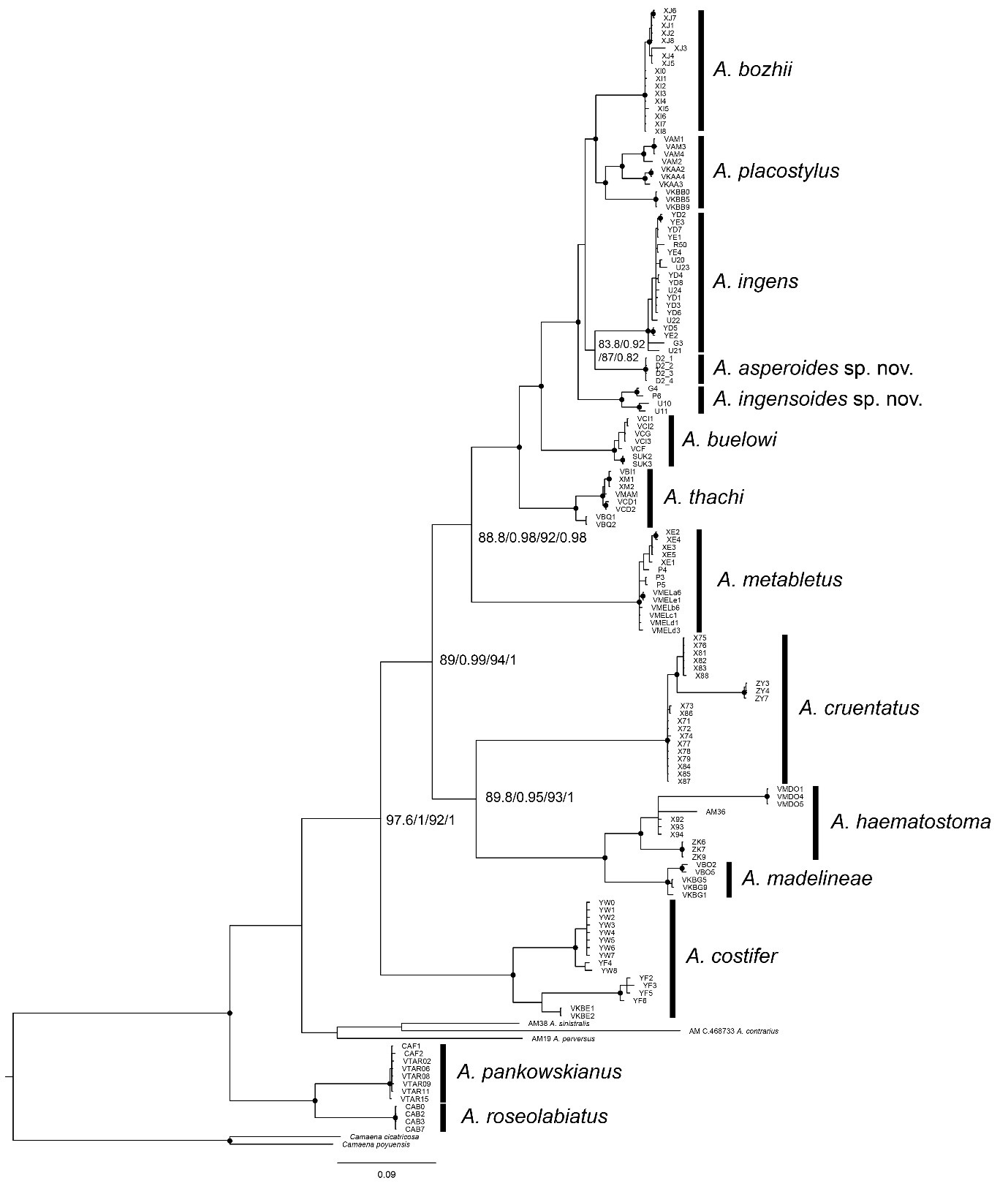

Supplement: Supplementary material 1 — Bayesian phylogenetic tree of Amphidromus spp. [file zookeys-1196-015_article-112146__-s001.jpg]
